# Harnessing the potential of hydrogels for advanced therapeutic applications: current achievements and future directions

**DOI:** 10.1038/s41392-024-01852-x

**Published:** 2024-07-01

**Authors:** Peilin Lu, Dongxue Ruan, Meiqi Huang, Mi Tian, Kangshun Zhu, Ziqi Gan, Zecong Xiao

**Affiliations:** 1 Nanomedicine Research Center, The Third Affiliated Hospital of Sun Yat-sen University, Guangzhou, 510630 PR China; 2 Department of Minimally Invasive Interventional Radiology, and Laboratory of Interventional Radiology, The Second Affiliated Hospital of Guangzhou Medical University, Guangzhou, 510260 PR China; 3 State Key Laboratory of Respiratory Disease, National Clinical Research Center for Respiratory Disease, National Center for Respiratory Medicine, Department of Respiratory and Critical Care Medicine, Guangzhou Institute for Respiratory Health, The First Affiliated Hospital of Guangzhou Medical University, Guangzhou, 510120 PR China; 4 Department of Stomatology, Chengdu Second People’s Hospital, Chengdu, 610021 PR China; 5 Hospital of Stomatology, Guangdong Provincial Key Laboratory of Stomatology, Guanghua School of Stomatology, Sun Yat-sen University, Guangzhou, 510055 PR China

**Keywords:** Cell delivery, Stem cells

## Abstract

The applications of hydrogels have expanded significantly due to their versatile, highly tunable properties and breakthroughs in biomaterial technologies. In this review, we cover the major achievements and the potential of hydrogels in therapeutic applications, focusing primarily on two areas: emerging cell-based therapies and promising non-cell therapeutic modalities. Within the context of cell therapy, we discuss the capacity of hydrogels to overcome the existing translational challenges faced by mainstream cell therapy paradigms, provide a detailed discussion on the advantages and principal design considerations of hydrogels for boosting the efficacy of cell therapy, as well as list specific examples of their applications in different disease scenarios. We then explore the potential of hydrogels in drug delivery, physical intervention therapies, and other non-cell therapeutic areas (e.g., bioadhesives, artificial tissues, and biosensors), emphasizing their utility beyond mere delivery vehicles. Additionally, we complement our discussion on the latest progress and challenges in the clinical application of hydrogels and outline future research directions, particularly in terms of integration with advanced biomanufacturing technologies. This review aims to present a comprehensive view and critical insights into the design and selection of hydrogels for both cell therapy and non-cell therapies, tailored to meet the therapeutic requirements of diverse diseases and situations.

## Introduction

The forefront of modern medical research has witnessed the emergence of innovative cell-based therapies (i.e., utilizing living cells as bioactive agents for disease treatment)^1–3^ and various promising non-cell therapeutic modalities.^4–6^ Despite their potential, these advanced therapeutic strategies still face significant hurdles in clinical translation.^1,7,8^ In cell-based therapies, transplanted cells are particularly vulnerable to variations in the physiological and pathological conditions of the host, such as oxygen tension, pH levels, osmolality, nutritional availability, and intercellular signaling.^1,9^ These environmental fluctuations may reduce cell survival and compromise their therapeutic functionalities, thereby diluting the efficacy of cell therapy.^10^ Moreover, the effective delivery of therapeutic cells presents another significant challenge, as illustrated by the frustrated homing and trafficking capabilities of natural killer (NK) cells.^11^ Influenced by the circulatory and lymphatic systems as well as endogenous signaling, these cells often fail to sufficiently penetrate solid tumors, leading to suboptimal therapeutic outcomes.^11^ Additionally, therapeutic cells may suffer from severe immune rejection and rapid clearance by the host’s immune systems, resulting in unsuccessful engraftment and failure to achieve desired therapeutic effects.

Non-cell therapeutic modalities, such as small molecule drug therapies, although less susceptible to environmental factors compared to living cell agents, still encounter many obstacles in clinical applications. These include unfavorable pharmacokinetics and low bioavailability with only a minor fraction of administered drugs reaching the bloodstream and effectively targeting the intended tissues or organs.^8^ Furthermore, systemic administration is often accompanied by undesirable adverse reactions,^12^ possibly dampening the compliance of patients. Bioactive agents, like proteins or genes, are prone to inactivation, degradation, and rapid clearance in the complex and variable in vivo microenvironments,^13–15^ substantially diminishing their therapeutic potential. Other non-cell therapeutic approaches, including physical intervention therapies, also face challenges in achieving selective targeting and effective treatment of deep tissues. To overcome these challenges, integration with other advanced technologies, particularly rapidly developed biomaterial technologies, appears to be a rational and feasible strategy for both cell- and non-cell therapeutics.

Hydrogels, which are highly hydrated three-dimensional (3D) polymeric matrices, hold substantial promise in medical and biomedical fields, owing to their excellent biocompatibility, chemical modifiability, and physical tunability, along with relatively straightforward processing procedures. These features position hydrogels as ideal platforms for both cell and non-cell therapy applications by fulfilling diverse requirements and significantly boosting therapeutic efficacy. Hydrogels have demonstrated potential in supporting cell viability and functionalities^16^ and in facilitating targeted delivery^17^ and controlled release of therapeutic agents.^18^ Therefore, the combination of hydrogels into cell- and non-cell therapeutics can not only ensure their therapeutic effectiveness and efficacy in vivo but also minimize systematic adverse effects,^19^ probably widening the therapeutic windows of these modalities.

Although early-generation hydrogels may exhibit limited flexibility due to their simplistic structures, restricting their applicability in complex therapeutic environments^20^ and dynamic release-based therapeutic strategies,^21^ current synthesis and modification technologies have matured enough to advance this material significantly. For instance, a variety of responsive hydrogels has been developed to react to specific biological and pathological stimuli (e.g., pH,^22^ temperature,^23^ reactive oxygen species (ROS),^24^ and other exogenous stimuli) to meet the intricate requirements of specific diseases and escalating clinical demands. By tailoring their chemical compositions, crosslinking strategies, and physical structures,^25–27^ newly developed hydrogels are equipped with versatile properties that allow them to directly regulate cellular behaviors, elicit specific cell phenotypes, and achieve controlled release and disease-specific targeting. As biomedical engineering technologies continue to evolve rapidly, driven by advances in cell therapy, immunotherapy, gene therapy, regenerative medicine, and a shift towards precision medicine, the applications of hydrogels are poised for further expansion.^28,29^

**Fig. 1:**
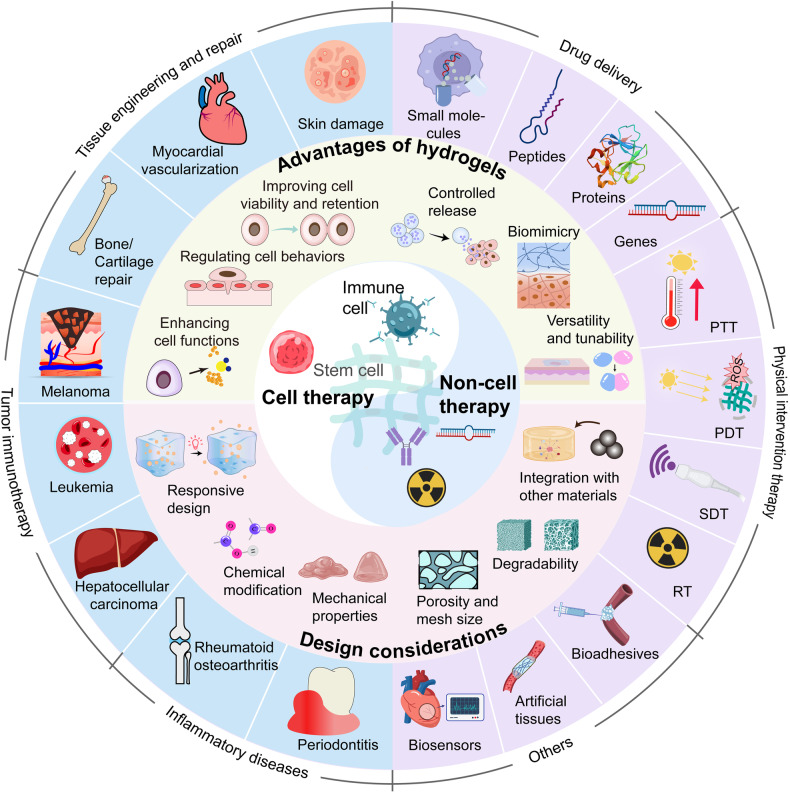
Schematic for applications of hydrogels in cell therapies and non-cell therapies, summarizing the advantages of hydrogels in both cell therapy and non-cell therapy contexts, discussing various design considerations for hydrogels in different scenarios, including responsive design, chemical modification, and modulation of mechanical properties, and concluding with an overview of the applications of hydrogels in cell therapies such as tissue engineering, tumor immunotherapy, and treatment of inflammatory diseases, as well as in non-cell therapies such as drug delivery and physiotherapy-mediated treatments. Adobe Illustrator was used to generate this figure

**Fig. 2:**
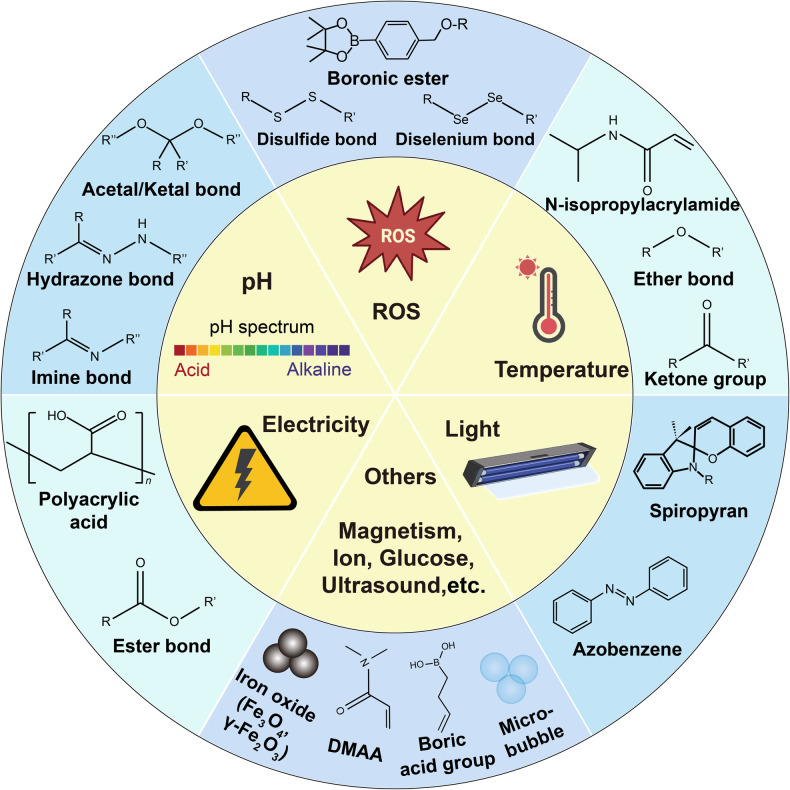
Sensitive groups or structures in stimuli-responsive hydrogel. This schematic summarizes the current responsive designs used in hydrogels for advanced therapies, including ROS-responsive, pH-responsive, and thermo-responsive designs, among others, and it also lists representative groups (e.g., boronic esters used in ROS-responsive designs) and structures (e.g., microbubbles used in ultrasound-responsive designs) used in these respective responsive designs. (Abbreviation: DMAA N,N-dimethyl acrylamide). Information is collected from published work.^23,40,43,46,47,819–822^ Adobe Illustrator was used to generate this figure

This review aims to provide a comprehensive understanding of the advancements and clinical applications of hydrogels in the context of cell and non-cell therapies (Fig. 1). We begin with an overview of hydrogels and the commonly used responsive design modes, including temperature, pH, ROS, light, electric and magnetic fields (Fig. 2). Subsequently, we outline mainstream cell therapies (e.g., stem cell therapy and adoptive cell transfer (ACT) therapy), summarizing their current translational application challenges and exploring hydrogels’ role in overcoming these. We then discuss the advantages and design considerations of hydrogels tailored for cell-based therapeutics, with a focus on the factors influencing cell therapy’s efficacy, the pivotal role of hydrogels, and the design principles on their physicochemical properties. For non-cell therapies, we also explore the advantages of incorporating hydrogels and their design preferences in this context. Then, we examine hydrogels’ efficacy in drug delivery, including small molecules, peptides, proteins, and genes, and in physical intervention therapies such as photothermal (PTT), photodynamic (PDT), sonodynamic (SDT), and radiation (RT) therapy, as well as their utilities in other non-cell therapy domains to serve as adhesives, artificial tissues, and biosensors. Additionally, we also review the current applications of hydrogel-mediated cell and non-cell therapy in clinical trials and discuss ongoing challenges, intending to provide a snapshot for future clinical translational applications of hydrogels. Finally, we conclude by outlining emerging developments that leverage advanced biomanufacturing technologies with hydrogels to highlight their prospective research directions and challenges.

## Overview of hydrogels and their responsive design

### Overview of hydrogels

Hydrogels, as 3D hydrophilic polymers, offer high water affinity, excellent biocompatibility, and versatile physical and chemical properties. Their applications span drug or cell delivery systems, biosensors, and regenerative medicine.^30^ Upon water absorption, the hydrophilic groups within hydrogels expand, establishing a stable network structure suitable for serving as a delivery platform for bioactive substances or pharmaceuticals. Remarkably, the 3D network of hydrogels can mimic the extracellular matrix (ECM) environment, promoting the growth and survival of encapsulated cells.^31–33^ Concurrently, biodegradable variants of these hydrogels can gradually degrade over time or under specific stimuli, releasing their contents without eliciting toxic side effects to the surrounding tissues.^34–36^ Furthermore, hydrogels can be engineered to undergo in situ gelation *via* chemical or physical cross-linking and be designed for injectability, ensuring sustained and controlled drug release at the targeted site, thus realizing the minimal invasiveness of lesions. Such localized administration provided by injectable hydrogels shows the potential of minimizing the therapeutic drug dosage, significantly reducing the adverse reactions associated with systemic drug exposure.^37,38^

### Responsive design of hydrogels

The design of responsive hydrogels entails the strategic manipulation of their structure and properties to demonstrate controlled reactions to specific stimuli, including temperature, pH, ROS, light, and electrical signals. The design aims to provoke physical or chemical alterations in the hydrogels under varied environmental conditions, thereby enabling precise regulation of their behavior and applications (Fig. 2).

#### Temperature-responsive hydrogels

Hydrogels incorporating polymers such as poly(N-isopropylacrylamide) can be sensitive to temperature variations. These hydrogels are characterized by their hydrophilic and swelling nature below their lower critical solution temperature, ~33 °C, and conversely, they become hydrophobic and contract above this threshold, resulting in a condensed state.^23^ Another type of temperature-responsive hydrogel composed of polymers containing ester bonds, can undergo hydrolysis at higher temperatures. Therefore, temperature-induced transitions in these hydrogels can include swelling, contraction, and degradation,^39^ which support the gradual release of drugs facilitating gradual drug release.

#### pH-responsive hydrogels

pH-responsive hydrogels are designed to be sensitive to changes in environmental pH, leading to modifications in their structure, morphology, or properties.^22^ This characteristic is particularly advantageous in cancer therapy, where the tumor microenvironments (TMEs) are often acidic due to metabolic byproducts such as lactate and carbonate, and further exacerbated by inadequate blood supply and poor lymphatic drainage. By reacting to these acidic conditions, pH-responsive hydrogels can improve drug delivery effectiveness and reduce treatment side effects. Additionally, the structural adaptations of these hydrogels can be harnessed for the development of biosensors, which are useful in detecting specific biomarkers or pH fluctuations in biological molecules and environmental monitoring.

#### ROS-responsive hydrogels

ROS-responsive hydrogels are engineered to contain ROS-sensitive bonds, such as disulfide bonds or diselenium bonds,^40^ enabling them to respond to ROS including superoxide radicals, hydroxyl radicals, and hydrogen peroxide. These hydrogels are specifically designed for environments with elevated ROS levels, commonly found in conditions such as inflammation, TMEs, and neurodegenerative diseases. For example, cancer cells often exhibit increased ROS levels due to abnormal metabolic activity and mitochondrial dysfunction, a contrast to normal cells. This variance in ROS levels creates a distinct opportunity for targeted cancer therapy,^41^ allowing ROS-responsive hydrogels to focus specifically on tumor tissues while minimizing effects on healthy cells.^24^

#### Light-responsive hydrogels

Light-responsive hydrogels incorporate elements that absorb or are sensitive to light at distinct wavelengths, triggering photoisomerization when exposed to light. A prevalent example is azobenzene, which adopts a cis configuration under ultraviolet light (365 nm) and reversibly shifts to a stable trans configuration under visible light (445 nm). The energy and wavelength of the light are crucial for their application in specific contexts. Ultraviolet light, characterized by its short wavelength and high energy, facilitates rapid responsiveness in hydrogel designs. However, the limited penetration depth of shorter-wavelength ultraviolet light confines its utility largely to superficial biological tissue layers, typically only reaching the skin’s surface.^42^ Consequently, the utilization of visible or infrared light, known for its deeper penetration into biological tissues, has become an innovative alternative.

#### Electro-responsive hydrogels

Electro-responsive hydrogels are composed of charged ions, which, upon exposure to an external electric field, undergo electrostatic forces causing ion movement.^43^ Positive ions gravitate towards the cathode, while negative ions move in the opposite direction, leading to a charge build-up within the gel. This charge disparity alters the water molecule arrangement and affects the interactions between polymer chains, resulting in localized structural changes such as gel swelling and contraction. The degree of these changes is influenced by various factors, including the strength and direction of the electric field, ion types and concentrations, migration velocity, temperature, and environmental conditions. Differing from stimuli like temperature, pH, or chemical substances, electric fields offer a more versatile control mechanism. The application of an electric field leads to the hydrogel’s reversible deformation, allowing it to regain its original shape once the field is removed. The principal advantage of this method is the precise control over current magnitude, pulse duration, and pulse intervals, facilitating accurate adjustments of the hydrogel’s shape, size, and structure.

#### Other-responsive hydrogels

Beyond the stimuli already mentioned, hydrogels have been specifically engineered to address certain practical requirements. Magnetically responsive hydrogels, for instance, can stimulate directional cell growth,^44^ offering potential in the structured regeneration of tissues, such as in post-fracture healing scenarios. Ion-responsive hydrogels are ideal for electrolyte-rich environments like tear fluid in the eye.^45^ Glucose-responsive hydrogels^46^ are crucial for monitoring physiological blood glucose levels and in advanced insulin management systems. Ultrasound-responsive hydrogels^47^ transmit energy efficiently with minimal loss, allowing for precise temporal and spatial control. Metabolite-responsive hydrogels target specific metabolites, triggering a response to those stimuli.

The responsive design of hydrogels not only initiates content release but also significantly controls the release rate. A key factor in therapeutic efficacy is the sustained release of contents, which directly impacts the duration of substance delivery within the body. If the degradation rate or swelling of the hydrogel carrier is fine-tuned to match the rate of tissue repair or regeneration, optimal therapeutic outcomes can be achieved. For example, temperature, pH, and electro-responsive hydrogels can modulate the gel’s decomposition, swelling, or cross-linking within specific parameters. Highly cross-linked hydrogels in a contracted state may have reduced permeability, resulting in a slower release rate. This variability is instrumental in achieving controlled expansion and release of the hydrogel.

In conclusion, there is considerable scope for enhancing material selectivity or developing new bio-responsive materials to better suit specific application needs. A thorough understanding of the particular requirements of hydrogels in diverse applications, material behavior under specific conditions, and control modes in practical use is essential for the design and preparation of hydrogels.

## Hydrogels for cell therapy

Cell therapy, at the forefront of therapeutic modalities, involves leveraging living cells to promote healing and combat diseases within the body. This innovative approach includes the administration of viable cells via injection, transplantation, or implantation for therapeutic benefits. Intuitively, cell-based therapy possesses unique innate therapeutic advantages over traditional drug treatments. Upon entering the body, these biologically active cells can rapidly adapt to and dynamically respond to various physicochemical stimuli and biological signals, as well as interact with the body’s native cells to perform their therapeutic functions.^48^ Such adjustable characteristics endow them with inherent superiority in combating intractable diseases, enabling malignant tumor regression or damaged tissue repair and regeneration.^49^

Cell therapy can be roughly divided into three main categories: stem cell therapy, ACT therapy, and targeted cell replacement therapy. Current predominant stem cell therapy primarily involves the use of hematopoietic stem cells (HSCs), mesenchymal stem/stromal cells (MSCs), and the promising, actively investigated induced pluripotent stem cells (iPSCs). By leveraging their self-renewal, multilineage differentiation, immunomodulatory, and chemotaxis capabilities, this modality holds the potential for treating a broad variety of diseases, including blood disorders, inflammatory diseases, and degenerative disorders, and facilitating tissue repair and regeneration. Unlike the former, ACT therapy typically utilizes autologous or allogeneic immune cells, in particular T cells, to combat malignancies, demonstrating clinical efficacy against hematologic malignancies, lymphomas, and certain solid tumors.^50,51^ Beyond cancer immunotherapy, its applications are being expanded to other refractory diseases, such as autoimmune diseases,^52^ where conventional treatments often present limited and merely palliative effects. The third classification, targeted cell replacement therapy focuses on exploiting terminally differentiated somatic cells to treat diseases by directly replacing or repairing damaged tissues, offering another promising approach to disease treatment.

Despite its wide applications and distinct advantages, cell therapy remains limited by a series of intractable treatment-related problems in practical applications, including challenges in targeted delivery, low cell survival rates, and functional inactivity in-vivo, as well as safety concerns. Primarily, the efficacy of cell therapy is underpinned by the effective migration and retention of cells at specific sites. While certain types of lymphocytes and stem cells do present disease-specific targeting potential owing to their intrinsic chemotactic properties, their homing capability is heavily dependent on chemokine gradients at the lesion areas, which may lead to therapeutic failures.^53–55^ For instance, tumor-infiltrating lymphocytes (TILs), known for their natural tumor tropism, still fail to localize to certain solid tumors because of reduced chemokine expression in immunosuppressive TME.^56,57^ This suggests that localized administration routes for cell therapy may offer more advantages in facilitating targeted delivery and precise treatment of solid tissue diseases over systematical infusion.

Furthermore, maintaining cell survival and functionality in vivo is another significant challenge in cell therapy. To address this, the co-delivery of cytokines alongside therapeutic cells is often necessary in vivo context. For instance, the co-administration of high-dose interleukin-2 (IL-2) with TIL therapy is a standard practice to promote the growth and activity of infused TILs.^57^ Likewise, IL-15 is widely utilized in ACT modalities, either to maintain NK cell survival in vivo^58^ or to boost chimeric antigen receptor T (CAR-T) cell efficacy by increasing effector T cell subsets.^59,60^ However, the systemic toxicity associated with these cytokines also underscores the necessity of more manageable administration methods and potentially alternative strategies to enhance the viability and functions of transplanted cells in the body. Beyond these above, the safety risks can be more complex with genetically engineered cells involved, such as on-target/off-tumor toxicity (OTOT) and cytokine release syndrome (CRS) caused by CAR-T therapy,^61^ or immunogenicity and tumorigenicity concerns with induced iPSCs.^62^ Admittedly, the extensive application of gene editing in the development of next-generation cell therapy products is beyond doubt in the current -omics era, aiming at more precise and personalized treatment.^63,64^ Nevertheless, more sophisticated genetic modifications of cells would inevitably lead to increased safety risks and uncertainties, especially manifested in multiple rounds of gene editing. Therefore, exploring alternative approaches to improve the efficacy of transplanted cells while reducing or simplifying genetic manipulation remains a necessity and promising area for research.

To overcome these challenges, significant strides and advancements have been achieved, especially evident in biomaterials technologies over recent decades. Hydrogels, in particular, have emerged as a promising and feasible tool in cell therapy. These highly tunable platforms do not merely serve as effective cell carriers for controlled and targeted cell delivery while showing the potential of improving cell survival and prolonging their retention within pathological areas; they can also function as a pseudo-extracellular matrix. By tailoring the properties of the hydrogel matrix, it is possible to impose specific, designed physicochemical stimuli on therapeutic cells to regulate their functions or even alter their fate. Consequently, this could potentially obviate some of the need for genetic manipulation while still amplifying therapeutic effects and mitigating adverse reactions.

In this section, we begin with an overview of the mainstream paradigms in cell therapy and their existing translational application challenges. From there, we provide a general discussion on the potential solutions by leveraging hydrogel systems. In the following subsection, we highlight some crucial influencing factors affecting the efficacy of cell therapy and explore how hydrogels can participate in these processes and exert their roles. From these discussions, we try to present the intrinsic advantages of hydrogels in the context of cell therapy. We then detail the principal considerations for their design, including their chemical composition, modifications, crosslinking methods, matrix stiffness, porosity and mesh size, dimensionality, degradation behaviors, and the possibility of integration with other materials. Subsequently, we catalog specific applications of hydrogels in cell therapy, such as tissue engineering and repair, tumor immunotherapy, and inflammatory disease treatment. In this last subsection, we also discuss the strengths and weaknesses of cell therapy, as well as the considerations for selecting it in different situations. We hope this part of the discussions could offer a snapshot for future researchers, assisting in the design and selection of hydrogels for cell therapy tailored to the therapeutic needs of different diseases and situations.

### Addressing challenges in cell therapy: from mainstream paradigms to hydrogel solutions

#### Mainstream cell therapy paradigms and their translational application challenges

##### Stem cell therapy

Stem cell therapy has undergone several iterations, from the first-generation products—multipotent somatic stem cells (e.g., HSCs and MSCs), to the pluripotent stem cells (PSCs)-based second generation, and onto the next generation of engineered stem cells that are enhanced with genetic modifications to either improve therapeutic functions or act as “Trojan horses” for delivering therapeutic drugs.^65^ The inception of stem cell therapy dates back nearly 65 years with the first clinical attempt to transplant HSCs-contained bone marrow for combating hematological cancers, marking the dawn of stem cell-based treatments.^66^ In April 2023, the U.S. Food and Drug Administration (FDA) granted formal approval to the first stem cell product—Omidubicel (omidubicel-onlv; Omisirge^®^)—an allogeneic HSC source for treating hematological malignancies and hemoglobinopathies.^67,68^ In parallel, several MSC-based therapies have also secured approvals, such as Remestemcel-L (Prochymal^®^, Osiris in Canada, 2012) for addressing graft-versus-host disease (GVHD) and Darvadstrocel (Alofisel^®^, TiGenix/Takeda in Europe, 2018) for treating Crohn’s disease-associated fistulas.^69,70^ These developments undoubtedly highlight the significant potential and emerging prominence of stem cell therapy in anti-inflammation treatments, regenerative medicine, and the newly developed next-generation products for oncology.

The therapeutic efficacy of stem cell therapy primarily arises from three mechanisms: their differentiation and regenerative capabilities, secretion of therapeutic molecules, and innate homing abilities. Firstly, stem cells possess self-renewal and differentiation potential into various specialized cell lineages including, but not limited to, nerve cells, muscle cells, blood cells, endothelial cells, osteoblasts, adipocytes, and chondrocytes.^71^ These abilities allow them to replenish endogenous cells or replace damaged tissues for tissue repair and regeneration. Furthermore, stem cells secrete a wide range of therapeutic cytokines, growth factors, and chemokines to modulate the local microenvironment of lesions. This modulation can not only orchestrate tissue repair but also help regulate body immunity, thereby promoting the elimination of harmful substances at lesion sites.^72^ For example, joint intra-articular transplantation of stem cells has been demonstrated to provide therapeutic benefits for the treatment of osteoarthritis. These benefits are likely mediated by the paracrine effect of MSCs, through the release of cell factors that act on chondrocytes. The bone morphogenetic proteins (BMPs) and the Wnt/β-catenin pathway have been identified as key players in this process.^73^ Additionally, the natural homing capabilities of stem cells enable them to migrate directly to sites of inflammation, damage, or tumors post-administration, which has been gathering increased attention, especially in the design of next-generation products.^74^

Taking MSCs as an illustration, unlike HSCs which are primarily utilized for treating blood disorders, MSC-based therapies have been applied to a broader spectrum of disease applications including cardiovascular diseases, digestive disorders, liver diseases, and arthritis.^75^ This versatility is attributed not solely to their multipotent differentiation potential but also to their immunomodulatory, anti-inflammatory, angiogenic, trophic, and anti-apoptotic properties. Beyond the previously mentioned approved applications for GVHD and Crohn’s disease, MSC-based products have also been successively authorized for treating subcutaneous tissue defects and repetitive and/or traumatic cartilage degeneration (including degenerative osteoarthritis) by the Korea FDA.^76,77^ However, the challenges of maintaining the viability, differentiation capabilities, and in situ retention of MSCs remain a somewhat clichéd yet pending issue. The significance of these matters is underscored by the inconsistent and often paradoxical outcomes of MSC-based clinical trials for myocardial infarction over the past two decades.^78^ While initially reported results appeared promising, subsequent research indicated that most infused MSCs were trapped in the lungs and swiftly eliminated by the host immune system, instead of being successfully engrafted.^79,80^ The therapeutic benefits, if existed, might be attributed to the MSCs-secreted cytokines and other trophic factors—which can function in anti-inflammation, angiogenesis, and immune modulation—rather than the direct differentiation and regenerative capacities of the cells themselves.^81^ Therefore, it is increasingly clear that providing a supportive platform for transplanted stem cells to sustain their viability, improve their retention, and shield them from immune attacks is crucial for successful engraftment and leveraging their differentiation and regenerative potential.

More recently, the next-generation MSCs (as well as neural stem cells (NSCs)) in clinical and preclinical trials are being investigated as drug-delivery carriers for delivering chemotherapy drugs or prodrug-converting enzymes, oncolytic viruses, therapeutic genes, or other cytotoxic agents targeting tumor cells for antitumor therapy.^82,83^ Several experiments have elucidated the mechanisms underlying MSCs’ tumor tropism, demonstrating that the tumor microenvironment contains many of the same inflammatory mediators as wound sites, which recruit MSCs to the tumor sites.^84^ For example, tumor cells may emit the chemoattractant stromal cell-derived factor-1 (SDF-1), which binds to C-X-C chemokine receptor type 4 (CXCR4) on MSCs, inducing their tumor homing.^85,86^ Other well-studied molecules, such as tumor necrosis factor-α (TNF-α), IL-6, and hypoxia-inducible factor 1-alpha (HIF-1α), provide further insight into MSCs’ tumor-homing properties.^87^ However, it should also be borne in mind that the immunosuppressive and angiogenic properties of MSCs could, in turn, heighten tumorigenic risks,^88^ presenting significant challenges for the clinical translation of MSCs as antitumor therapy. Additionally, their migration and homing efficiency post-intravenous injection is insufficient, thereby necessitating the development of more effective strategies, such as the combination with click chemistry and bio-orthogonal reactions, to enhance their targeting to tumor sites.^89^

The second-generation therapy is based on PSCs, characterized by their infinite proliferation and pluripotent differentiation capabilities. Human embryonic stem cells (hESCs) and iPSCs are under active investigation in both fundamental research and clinical trials, spanning a range of diseases, such as Parkinson’s disease, retinitis pigmentosa, amyotrophic lateral sclerosis, and spinal cord injury.^90^ iPSCs, which circumvent the ethical issues surrounding hESCs, have emerged as a focal point of interest. An exemplary application involves iPSC-derived products for the replacement of pancreatic β-cell in the treatment of type I diabetes (NCT02239354). Moreover, since 2019, clinical trials for iPSCs-enabled “Off-the-Shelf” cell therapy have commenced, potentially heralding a new era of a one-cell-fits-all solution if the technique matures in the future.^91^ However, this promising approach is not without its challenges, primarily concerns over the potential for ectopic tissue growth or/and tumorigenicity, which may arise from residual iPSCs or immature progenitors in the final cell products. The risk of tumorigenicity is further heightened by changes in telomere length, chromosomal instability, and genetic mutations resulting from prolonged in vitro culturing, introducing safety uncertainties. In addition, addressing iPSCs’ immunogenicity to prevent immune rejection of iPSCs-derived cells is another barrier to their translational application, especially critical in the design of “Off-the-Shelf” products. Conceivably, advancements in genome and epigenome modification techniques, coupled with advanced biomaterial technologies, could provide viable solutions for overcoming these challenges in the next-generation development of iPSCs.^91^

##### Adoptive cell transfer therapy

ACT therapy involves utilizing ex-vivo expanded autologous or allogeneic immune cells, with or without genetic modifications, and reinfusing them back into patients to bolster antitumor responses and eliminate cancer cells. As a groundbreaking approach in cancer treatment, it has witnessed explosive growth in recent years. Remarkably, the volume of clinical trials for ACT, even those conducting T-cell trials individually, has now outnumbered all stem cell trials and significantly exceeded those involving tissue-specific cells.^49^ It is particularly heartening that the first TIL therapy, Iovance’s lifileucel (Amtagvi), received FDA approval for melanoma treatment just in February 2024.^92^ This marks a significant stride toward solid tumor treatment with ACT therapy, following the regulatory endorsement of CAR-T therapy for hematologic malignancies, such as acute lymphoblastic leukemia (tisagenlecleucel)^93^ and large B cell lymphoma (axicatagene ciloleucel).^94^ Additionally, the encouraging clinical results from natural killer (NK) cells suggest the imminent arrival of another cell therapy modality on the market.^95,96^ These developments greatly bolster hopes for future ACT therapy to further broaden its application spectrum.

However, the prohibitive costs of ACT therapy present a formidable barrier for many patients, and the lengthy manufacturing process, often spanning several weeks, poses an additional challenge for terminal-stage patients. For these individuals, their conditions may rapidly deteriorate, potentially leading to death during this waiting period. Consequently, ongoing research into “Off-the-shelf” third-party cell sources, along with the development of new engineering techniques for in vivo gene modifications, is so crucial and necessary.^50^ These efforts aim to streamline manufacturing processes, reduce the waiting time for patients, and cut down costs, ultimately making ACT therapy more accessible and feasible for a wider patient population. In this subsection, we list the currently predominant ACT therapies and their translational challenges. The advantages and limitations of each therapy are summarized in Table 1.

**Table 1:** Comparison of advantages and limitations between different types of adoptive cell therapy

ACT type	Advantages	Limitations
CAR-T cell therapy	1. Target specificity and HLA independence 2. Adjustable and customizable CAR structure enhancing flexibility 3. High response rates and long-term benefits	1. Limited antigen recognition (only surface antigens) 2. Adverse reactions: CRS, OTOT, off-target reactivity 3. Limited efficacy in solid tumors
TCR-T cell therapy	1. Potent target specificity 2. Recognition of both surface and intracellular antigens 3. Activity in multiple tumor types	1. MHC or HIL allele restriction 2. Adverse reactions: CRS, OTOT, off-target reactivity 3. Suffering from antigen loss
TIL therapy	1. Natural and personalized sources 2. Diverse TCR clonality and superior tumor tropism, advantageous in solid tumor treatment 3. Low off-target toxicity	1. Not accessible and feasible to all tumor types due to invasive process to acquire 2. Prone to a dysfunctional or exhausted states
Treg therapy	1. Immune homeostasis maintenance and autoimmunity control 2. Promising role in autoimmune diseases 3. Potential to induce transplant tolerance	1. Suppression of anti-tumor immune responses 2. Difficulty in controlling Treg dynamics
NK cell therapy	1. No need for HLA matching 2. Broad spectrum anti-tumor activity 3. Safe profile due to non-specific antigen recognition	1. Short survival time 2. Relatively weaker anti-tumor effects
DC therapy	1. Immunoregulation 2. Broad specificity 3. Strong plasticity	1. Short survival time 2. Suboptimal antigen presentation efficiency and viability
Macrophage therapy	1. Strong phagocytic capability 2. Inflammatory effect modulation	1. Insufficient adaptability to tumor cell variations 2. Ambiguous therapeutic efficacy due to uncertain cellular subtype ratios

Information in Table 1 is collected from published work^101,107,117,790–793^

**Fig. 3:**
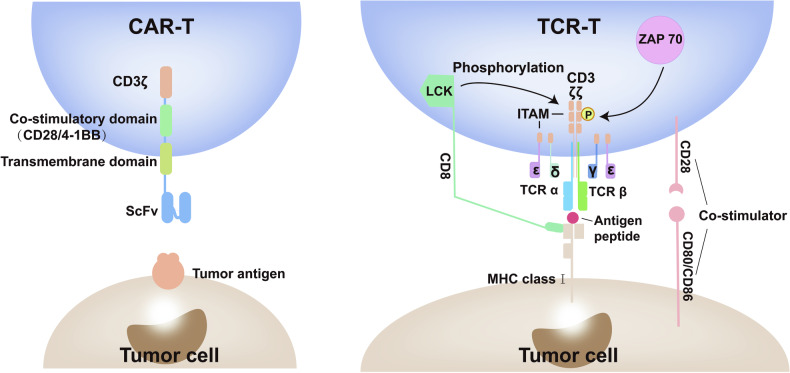
Structural comparison of CAR-T and TCR-T. The CAR structure of CAR-T cell includes an outward-facing antigen recognition domain that identifies specific antigens on cancer cell surfaces, a transmembrane domain anchoring the CAR to the T cell membrane, a co-stimulatory domain (e.g., CD28 or 4-1BB) and an activation domain (typically the CD3ζ chain), both of which work together to transmit signals that activate the T cell. TCR-T cells comprise two distinct protein chains (α and β chains) that interact with peptide-MHC complexes across several regions. Activation of the TCR depends on the CD3 complex and additional costimulatory signals (e.g., CD28). Information is collected from published work.^790,823,824^ Adobe Illustrator was used to generate this figure

*CAR-T cell therapy*: CAR T-cell therapy represents a pivotal branch in adoptive T-cell therapy. It involves extracting the patient’s T cells and genetically engineering them by introducing chimeric antigen receptors, enabling these T cells to identify and eliminate cells expressing the homologous target antigen more effectively.^97^ Unlike traditional therapies, CAR-T cells can directly recognize tumor antigens, independent of human leukocyte antigen (HLA) restriction and tumor main histocompatibility complex (MHC) expression (Fig. 3). Currently, CAR-T cell therapy has demonstrated significant success in treating hematologic malignancies.^98^ However, its clinical effectiveness varies significantly among patients with solid tumors. This variation is attributed to multifaceted factors, such as limited tumor antigen specificity, inadequate trafficking and infiltration, short-lived persistence, and reduced efficacy of CAR-T cells in immunosuppressive TMEs.^2^ Besides its limited efficacy in solid tumors, safety issues with CAR-T therapy are another significant challenge in its clinical translation. The most common serious adverse effect is CRS, occurring in over 70% of patients, which can further lead to severe neurotoxicity. This is mainly caused by a rapid and uncontrolled proliferation of effector T cells in the bloodstream post-administration of CAR-T products.^99^ Furthermore, on-target/off-tumor (OTOT) toxic effects may cause CAR-T cells to mistakenly attack normal tissues and healthy cells, leading to severe, potentially fatal outcomes.^100^ Addressing these concerns may benefit from strategies that allow more controllable and localized release of effector cells as well as administering anti-inflammatory cytokines targeted to minimize unwanted systemic effects.

*TCR-T cell therapy*: TCR-T, another significant branch of adoptive T-cell therapy, utilizes T-cell receptors (TCRs), which are membrane proteins crucial for antigen recognition on T-cell surfaces. By introducing modified TCRs, TCR-T cells gain the ability to specifically recognize and target antigens, identifying abnormal cells through peptide-antigen complexes bound to MHC molecules (Fig. 3). Distinct from CAR-T cells that mainly target surface antigens on tumor cells, TCR-T cells can detect intracellular antigens, offering a strategic advantage in solid tumor treatment.^101^ However, the HLA restriction of TCR-T cells limits their broad applicability in clinical settings due to the vast diversity of HLA types among patients. The off-target adverse effects of TCR-T cells, caused by receptor cross-reactivity with healthy cells, represent another challenging hurdle for their clinical adoption.^102^

In addition to CAR-T and TCR-T cells, ongoing research is delving into γδ T cell subsets, which present unique roles in immune responses and show promise as cancer immunotherapies. The TCRs of γδ T cells, composed of distinct γ and δ chains, differ markedly from the αβ TCRs of typical T cells. These γδ T cells recognize antigens in a non-MHC-restricted manner, directly identifying unique antigens like lipids and phosphoric components. Their streamlined TCR structure and shorter signal transduction pathways enable rapid targeting and destruction of cancer cells, playing an immediate role in immune defense. Given γδ T cells’ tumor recognition abilities, research is exploring the potential of CAR-γδ T cells as a new cellular therapy in tumor adoptive immunotherapy, akin to CAR-T cells.^103^ Clinical trials involving CAR-γδ T cell therapy have primarily focused on hematologic malignancies (NCT05388305, NCT02656147, NCT06056752). Moreover, these cells can be easily isolated, expanded, and activated ex vivo from patients’ peripheral blood, making their clinical application more feasible.

*TIL therapy*: TIL therapy, a significant advancement in solid cancer treatment, involves isolating lymphocytes from tumor tissues, culturing them to increase their numbers dramatically in vitro, and then reintroducing them into the patient. Unlike CAR-T and TCR-T cells, TILs, sourced from deep within tumor tissues, do not require genetic modification. Stimulating these isolated cells with IL-2 can result in an expansion of up to 1000-fold, yielding a substantial number of TILs. This therapy’s key advantage is the reintroduction of a large volume of TILs, which, originating from the tumor itself, demonstrate high tumor recognition and exhibit broad-spectrum cytotoxicity against various solid tumors in clinical practice. However, in TMEs, the efficacy of TILs might be hindered by immune checkpoint pathways, particularly the programmed cell death protein-1 (PD-1)/programmed cell death-ligand 1 (PD-L1) pathway.^104^ The immunosuppressive milieu fostered by tumor cells often leaves TIL-T cells (TIL-Ts) ineffective, exhausted or even leads to their death due to the deficiency of essential survival cytokines.^105^ Therefore, it is of paramount importance to establish an integrated training court to unleash the full potential of TIL-Ts and enhance their intrinsic antitumor capabilities to effectively target and eradicate tumor cells.^105^ This includes reinvigorating TIL-Ts from the states of incompetence or dormancy and safeguarding them from cellular death.^105^

*Treg therapy*: Regulatory T cells (Tregs), essential for moderating immune responses, play a pivotal role in maintaining immune system balance and controlling hyperactive immune reactions and are crucial in autoimmunity, immune tolerance, and preventing excessive inflammation. Tregs therapy, primarily through autologous Tregs infusion and to a lesser extent CAR-Tregs infusion, is emerging as a promising treatment. It is particularly relevant in preventing graft-versus-host disease (GVHD) in organ transplantation, focusing on ex vivo expanded Tregs. Research indicates that these cells are effective in preventing GVHD.^106^ Tregs have become increasingly significant in autoimmune disease research, including rheumatoid arthritis, systemic lupus erythematosus, and inflammatory bowel disease.^107^ Additionally, Tregs infusions can induce transplant tolerance, reducing the need for long-term immunosuppressive drugs, thus lowering associated morbidity/mortality risks.^108^ Despite this progress, maintaining the viability and stability of Treg cells at lesion sites remains a formidable challenge in Treg-based immunotherapy.^109^ For instance, while Treg cells show promise in treating ocular inflammatory disorders, the survival rates of these transplanted cells within the ocular microenvironment are often unsatisfactory. This may be explained by the complexities of the ocular microenvironment where pro-inflammatory cytokines can induce Tregs to differentiate towards the pro-inflammatory Th17 subtype, worsening inflammation.^110^ Consequently, to achieve clinical success in cellular therapy, endeavors must be made during the cellular treatment process to shield the transplanted cells from the adverse effects of the local microenvironment.^109^

*NK cell therapy*: NK cell therapy, which includes both autologous and allogeneic types, predominantly employs autologous NK cells to mitigate immune rejection risks. NK cells are characterized by their non-specific antigen recognition and lower incidence of adverse events, making them viable for treating various cancers and certain infectious diseases. Nonetheless, their non-specific mode of action may lead to less potent cytotoxicity compared to CAR-T and TCR-T cells.^111^ Additionally, the significant variability between individuals can affect the consistency of therapeutic outcomes. These limitations have spurred the exploration of CAR modification in NK cells, with some research applying advanced CAR structures from CAR-T therapy to create enhanced CAR-NK cells.^112^ However, adoptive NK cell therapy remains restricted in certain therapeutic domains. The frequency and functionality of NK cells within both the circulation and TMEs have been observed to decline across various cancers (e.g., hepatocellular carcinoma), which is strongly linked to postoperative tumor recurrence and adverse outcomes.^113^ Specifically, in the setting of triple-negative breast cancer, the efficacy of adoptive NK cell therapy is severely reduced due to immune evasion tactics employed by the cancer.^114^

*APC therapy*: Dendritic cells (DCs) and macrophages, as primary antigen-presenting cells (APCs) within the body, play pivotal roles in adoptive cell immunotherapy. Therapies including DCs involve the collection of patient’s own DCs, binding them with tumor-associated antigens, and subsequently culturing and activating them ex vivo. The activated DCs are then reinfused into the patient to stimulate the immune system’s response to the tumor. Such DCs are referred to as “immunomodulatory DCs” or “DC vaccines”^115^ and are reintroduced into the patient, thereby enhancing the immune system’s tumor response. Such DCs can also facilitate the development of vaccines against viruses, bacteria, and other pathogens by loading antigens onto DCs to provoke immune responses, including antibody production and immune memory, thus bolstering resistance to infections.^116^ Macrophages can adapt to their environment both morphologically and functionally, with M1-type macrophages exhibiting pro-inflammatory functions and M2-type macrophages showing anti-inflammatory activities. Research has demonstrated that M1 macrophage differentiation can be induced through low-dose irradiation in vitro. These macrophages, once injected into the patient *via* intravenous or intraperitoneal routes, can alter the tumor’s immune-suppressive microenvironment. Similarly, CAR-modified macrophages, designed for enhanced tumor recognition and cytotoxicity, could significantly counteract the immune-suppressive effects within the TMEs.^112^ Nevertheless, clinical trials involving DC vaccines and macrophages have not yielded satisfactory outcomes yet. The short-term survival of transferred DC vaccines would limit the duration of tumor antigen presentation,^117^ and the conventional delivery system for DC-based tumor vaccines fails to sustain the activation needs of the immune system.^118^ Additionally, the therapeutic effectiveness in inflammatory, injury, or tumor settings can be substantially influenced by the proportion of M1 and M2 macrophage subtypes.^119^ Thus, to optimize the immunotherapeutic efficacy of these approaches in vivo, two critical issues must be addressed: 1) maintaining the efficiency and vitality of DC antigen presentation in vivo, and 2) effectively managing macrophage polarization.

*Others*: Adoptive immunotherapy also employs cells such as lymphokine-activated killer cells^120,121^ and cytokine-induced killer cells.^122,123^ These cells undergo ex vivo expansion and activation to enhance their anti-tumor capabilities. They are then reintroduced into the patient, increasing their effectiveness in recognizing and attacking tumor cells, thereby offering a wider range of cancer treatment options. Currently, some of these methods are still in the research phase and not extensively used in clinical practice. The practical implementation and efficacy of these therapies require further investigation and validation. Although they have seen limited use in clinical trials, their widespread application in treatment remains limited due to ongoing research into their efficacy and safety. Therefore, innovative approaches are essential to augment the therapeutic impact of these cells.

In ACT, targeting tumor cells directly by utilizing natural receptors on cells is an effective strategy to achieve therapeutic effects. For example, the interaction between the NKG2D receptor on T cells and ligands (such as MICA, MICB, and ULBP 1-4) present in solid tumors and hematologic malignancies triggers tumor cell lysis through perforin and granzyme B release.^123^ Additionally, enhancing the recognition and elimination of pathological cells, such as cancer cells, can be achieved by exogenously introducing CAR to immune cells. CAR design aims to enhance immune cells’ antigen recognition, increasing their efficacy against pathological cells. These receptors not only mediate cytotoxicity but also promote cytokine production, including interferon- γ (IFN-γ), tumor necrosis factor-α (TNF-α), and IL-2.^123^ These cytokines are pivotal in triggering immune responses and physiological processes like inflammation, intensifying tumor destruction. For CAR-modified T cells, NK cells, DCs, and macrophages, cytokine release post-activation is crucial in immune modulation and tumor therapy by recruiting other immune cells.^124^ This cytokine-mediated mechanism enhances the anti-tumor response, aiding in tumor cell recognition and clearance. Furthermore, activated cells form complex networks in cellular therapy, contributing to a comprehensive immune response through signal transduction and interaction with immune cells, inflammatory mediators, and pathological cells.^125^ In summary, cellular therapy mechanisms are multi-layered and complex. Activated cells release cytokines, establishing communication networks among cells and regulating immune cells.^124^ Investigating these networks and interactions is crucial for full understanding of cellular therapy mechanisms. A detailed examination of factors influencing cell functionality will be discussed subsequently.

##### Targeted cell replacement therapy

In addition to stem cells and immune cells, a diverse array of differentiated somatic cells, including Schwann cells,^126^ fibroblasts,^127^ osteoblasts,^128^ cardiomyocytes,^129^ hepatocytes,^130^ and pancreatic islet cells^131^ has been extensively utilized for specific disease treatments. Unlike stem cells, these mature cells, having already undergone differentiation, obviate the need for a prolonged differentiation process, thus shortening the treatment timeframe. Moreover, the transplantation of mature cells poses fewer risks than those associated with the stem cell differentiation process, such as heterogeneity in differentiation, reduced viability, and the potential for tumorigenesis.^62^ The intrinsic advantage of mature cells is their specific functionality and differentiated state, making them more efficacious for therapeutic uses. The cell sources of this modality can also be derived from autologous or allogeneic types. Autologous therapy, which employs the patient’s own cells, minimizes rejection reactions but may be limited by the cells’ insufficient endogenous activity, particularly in treating endocrine disorders such as diabetes, hypoparathyroidism, hypothyroidism, and adrenal insufficiency.^132^ To address these, the development of allogeneic products with robust functionality and acceptable immunogenicity is essential and imperative, especially for the transplantation of parathyroid, thyroid tissue, and adrenal cortex cells.^132^

Despite the slower pace of research progress in targeted cell replacement therapy compared to other cell therapy approaches, recent advancements have been noteworthy. For example, the U.S. FDA’s recent approval of the allogeneic pancreatic islet β-cell therapy, Lantidra, for treating type I diabetes, signifies a groundbreaking development in the field. Lantidra not only facilitates achieving target blood glucose levels for patients but also offers an extended duration of action compared to conventional insulin therapies, thus representing a significant leap forward in targeted cell replacement therapy.

However, as mentioned above, the applicability of mature cells is generally restricted to certain diseases or injuries, necessitating a thorough evaluation of the cell types’ characteristics to tailor the treatment to the patient’s specific health requirements effectively. What’s more, it is critical to recognize that the transplantation of mature cells is not entirely free from safety risks and ethical concerns.^133^

#### Exploring hydrogels’ role in overcoming cell therapy challenges

To date, the majority of cell therapy products approved by the FDA are derived from autologous cell sources. However, allogeneic cells, or more precisely, “Off-the-Shelf” products, represent the future of this field due to their advantages in large-scale manufacturing, source availability, and cost-effectiveness. A critical challenge in the use of allogeneic cells is to overcome immunological rejection. Yet, the traditional approach of taking immunosuppressants may expose patients to a high risk of severe infections, while novel genetic strategies, such as engineering HLA-knockout cells, could lead to more hidden and uncontrollable issues. In this context, hydrogels offer a relatively straightforward and safe alternative for mitigating the immune rejection of allogeneic cells and may serve as a treatment option for localized diseases. Encapsulating transplanted cells within a biocompatible hydrogel matrix can create an immuno-isolating physical barrier between allogeneic cells and the host, effectively shielding the transplanted cells from cell-to-cell contact-mediated immune response, including recognition, lysis, and phagocytosis. Meanwhile, the inherent hydrophilic nature and porous structure allow a bidirectional exchange of oxygen, nutrients, cellular wastes, and therapeutic factors, thus extending cell survival and ensuring therapeutic efficacy in vivo. This feasibility has been proven in the use of hydrogel-coating for stem cell-derived islets.^134^ Such a gel-coating successfully thwarted the host immune response against allogeneic islet engraftment while permitting the transport of glucose, oxygen, and the secretion of insulin from the transplanted cells, demonstrating great potential in reversing diabetes in an MHC-mismatched model. Likewise, encapsulating MSCs within a type I collagen hydrogel has been shown to decrease their immunogenicity and sustain their viability, significantly reducing microglia activation and astrocyte recruitment.^135^ This approach effectively addressed the common issue of poor cell survival in the central nervous system (CNS) after transplantation. Therefore, employing biocompatible, well-tolerated hydrogels, particularly those derived from natural extracellular matrix components, could be a feasible way of supporting cell survival and successful engraftment by diminishing immune rejection.

Furthermore, minimizing the waiting period of patients is another crucial goal for the future of cell therapy. Aside from leveraging allogeneic or universal cell sources prepared in advance, researchers are exploring hydrogel platforms for rapid ex vivo expansion or in vivo genetic modifications, thereby streamlining manufacturing processes. For example, employing a 3D zwitterionic hydrogel culture has resulted in a 73-fold increase in the frequency of hematopoietic stem and progenitor cells, while preserving their long-term repopulating capabilities.^136^ This rapid expansion and maintenance of self-renewal capabilities may be attributed to the gel’s 3D structure, super-hydrophilic, antifouling, and zwitterionic properties. These characteristics can make this culture system more closely mimic the in vivo niche and effectively minimize non-specific protein absorption and interactions, thus avoiding unintended differentiation. Similarly, Jie and colleagues developed a self-assembling peptide hydrogel tailored in stiffness and adhesive ligand density, enabling the rapid proliferation of CAR-T cells and considerably shortening the processing time to merely 3 days.^137^

More strikingly, several in vivo reprogramming manufacturing paradigms have emerged to achieve genetic modifications directly within the target site, drawing considerable attention. For instance, Chen and co-workers developed an injectable hydrogel based on brain ECM-mimetic peptides, designed for transporting CAR plasmid-laden nanoporters into the resection cavity of glioblastoma multiforme (GBM). This CAR gene-laden hydrogel successfully reprogrammed macrophages in situ, yielding CD133-specific CAR macrophages with enhanced phagocytic activity against glioma stem cells. When combined with locoregionally delivering anti-CD47 antibodies using this hydrogel reservoir, the approach effectively prevented postoperative relapse of GBM and induced long-term antitumor immunity in mouse models.^138^ Likewise, an alginate-based cryogelated scaffold has demonstrated the capability of in vivo CAR-T cell production by co-loading human peripheral blood mononuclear cells and CD-19-encoding retroviral particles. This scaffold streamlined T cell activation, expansion, and transduction into a single in situ process, remarkably shortening the manufacturing timeline from the conventional several weeks to just a single day.^139^ More recently, another noteworthy instance showcased a cationic polymer-based supramolecular hydrogel’s ability to reprogram CAR-T cells in situ to target solid tumors, employing plasmid CARs.^140^ These advancements in hydrogel-mediated techniques substantially facilitate faster manufacturing procedures and herald a promising future for the next generation of cell therapy.

Lastly, in terms of the safety concerns, especially associated with ACT therapy, hydrogels still present a feasible solution, owing to their flexibility and versatility, which allow for the controlled pharmacokinetics and biodistribution of transferred cells. Fundamentally, hydrogel-based matrices can establish a localized depot for the relatively prolonged release of transferred cells. For one thing, this cell-loaded depot can enable a controllable egress rate of transferred cells by tailoring the hydrogel’s degradation patterns, dynamic deformation, and swelling properties, thereby reducing the risk of CRS, which typically results from the rapid influx of effector immune cells into the body post-administration.^141^ In this context, the duration of ACT therapy’s effectiveness would also be extended due to the more sustained presence of transferred cells in vivo. For another, this localized platform can minimize the exposure of transferred cells to non-target tissues, either by directly delivering cells in focal sites or by recruiting cells infused systematically, thus diminishing or even averting OTOT toxicity effects.

In addition, hydrogel-based approaches provide a strategic method to circumvent the side effects associated with systemic exposure to supportive cytokines typically required in the co-administration with adoptive transferred cells. These cytokines or supportive agents (e.g., growth factors, antibodies, or costimulatory molecules) can be securely immobilized into the hydrogel’s backbone through chemical modifications like heparin-functionalization or copper-free click reactions, or be maintained as a soluble form within the gel via physical interactions, enabling controlled release at targeted sites. For example, a hydrogel composed of a hydrophobically modified cellulosic polymer mesh has been developed for co-delivering CAR-T cells and IL-15, a potent T cell activator.^59^ The hydrophobic interaction between the cytokine and the gel’s components substantially slowed the rapid diffusion of IL-15 from the gel, offering a safer and more enduringly effective modality for CAR-T therapy. More advanced strategies can be exemplified by a design of attaching nanogel backpacks carrying IL-15 to the surface of transferred T cells to boost their antitumor activity.^142^ This innovative approach confined the stimulatory effects of IL-15 to the transferred cells, rather than causing systemic effects, significantly enhancing the safety of ACT therapy. These strategic approaches for hydrogel-based cell therapy enhance both the safety and efficacy of therapeutic applications, underscoring their potential in addressing current challenges and shaping the future of cell therapy.

### Advantages and design considerations of hydrogels for cell therapy

In the last subsection, we have outlined the promising applications of hydrogels in cell therapy, emphasizing their capability to reduce the immunogenicity of transplanted cells, streamline manufacturing processes, and mitigate systematic adverse effects. Here, we will delve into a comprehensive discussion of the critical factors affecting the efficacy of cell therapy, with a focus on strengths of hydrogels in this context. More importantly, we also examine the primary design considerations for hydrogels suitable for cell therapy, including their chemical composition, modifications, crosslinking methods, matrix stiffness, porosity and mesh size, dimensionality, degradation behaviors, and the possibility of integration with other materials. Understanding how these design factors can direct cell behaviors and fate is essential for leveraging hydrogels to enhance the therapeutic outcomes of cell therapy.

#### Influencing factors on the efficacy of cell therapy and the role of hydrogels

The efficacy of cell therapy is determined by a wide range of factors, including but not limited to soluble factors, cell-cell interactions, matrix dynamics, and microenvironmental conditions such as oxygen tension, pH levels, osmolality, and nutritional availability. These external factors, upon activation of cellular surface receptors, can initiate a cascade of intracellular signaling pathways and gene transcription events, thereby leading to diverse cellular responses and behaviors. These factors hold such a significant importance in the success of cell therapy but also represent the challenges in achieving successful engraftment. By incorporating hydrogels into cell therapy strategies, it is possible to tailor the microenvironment to optimize conditions for transplanted cells, thereby enhancing their functionality and therapeutic potential. This approach seeks to create safer, more dependable, and efficacious cell-based treatments.

##### Cytokines and growth factors

Cytokines and growth factors are highly crucial in intercellular interactions and signal transduction, significantly affecting various cellular processes, including proliferation, differentiation, migration, and other physiological functions. In cell therapy contexts, these molecules are frequently used both as pretreatment in ex vivo cultures and co-delivered with transplanted cells in vivo to ensure successful engraftment and enhance cellular functions. For example, the addition of IL-2 is a standard procedure for the rapid TIL expansion ex vivo, with evidence showing that a medium containing 6000 IU/mL IL-2 can yield a substantial number of therapeutically viable cells with maintained antigen specificity and activity post-expansion.^143^ Similarly, the co-administration of IL-2, IL-4, and IFN-γ is crucial for sustaining the survival and preserving the transplant-tolerant phenotype of alloantigen-specific CD4^+^T cells.^144^ Common practices also include the use of IL-12, IL-15, and IL-18 to maintain NK cell vitality and stimulate ex vivo expansion,^145,146^ employing VEGF, FGF-1, or FGF-2 to support endothelial cell proliferation,^147,148^ and applying transforming growth factor-beta1 (TGF-β1), TGF-β3, or BMP-2 for the chondrogenic or osteogenic differentiation of MSCs.^149–151^

In contrast, however, it is usually not that reliable for in-vivo administration of these factors primarily because of their short half-lives (only a couple of minutes in some cases^152,153^), poor stabilities, and safety concerns. Hydrogels can create a conducive environment for the stability and bioactivity of these factors, protecting them from enzyme degradation and optimizing their release patterns through either physical interactions or chemical coupling. For instance, BMP-2, part of the TGF-β superfamily and crucial for bone remodeling and homeostasis, is FDA-approved for certain orthopedic procedures.^154^ Yet, its wider applications are considerably hindered by its structural instability, rapid degradation (less than 10 min after I.V. administration^155^) as well as troublesome adverse effects associated with its supra-physiological concentration requirement.^156^ Interestingly, research has shown BMP-2’s tendency to aggregate at neutral pH, reducing its efficacy in physiological fluids,^157^ whereas its stability and bioactivity can be preserved in an acidic environment (pH~4.5).^158^ Based on these, Yan and coworkers developed a hyaluronic acid (HA)-based hydrogel that offers an acidic matrix to stabilize BMP-2.^159^ To overcome the low affinity of BMP-2 for acidic matrixes and prevent burst release,^160^ they modified the initial carboxylic acid protonation states of HA. This adjustment rearranged the HA chain, greatly increasing Van der Waals interactions between the HA chain and BMP-2, thereby extending the release kinetics of BMP-2 to 28 days while preserving its stability and bioactivity for several weeks. This innovative hydrogel approach may reduce the dosage requirement of BMP-2 in future clinical settings.

Notably, certain biopolymers, such as heparin and fibronectin, as well as engineered binding motifs (e.g., DNA or RNA aptamers) naturally present a high affinity for specific growth factors or cytokines.^161^ For instance, Heparin, a highly sulfated glycosaminoglycan, can utilize its strong negative charge to attract positively charged factors through electrostatic interactions, such as VEGF, TGF-β1, TGF-β2, FGF-2.^162,163^ By integrating these binding elements into the hydrogel system, it is possible not only to regulate the release of exogenous factors but also to capture and retain endogenous growth factors in situ. An illustrative example is an injectable hydrogel consisting of tyramine-modified konjac glucomannan (KGM) and heparin. KGM, with its D-mannose and D-glucose backbone, exhibited a high affinity for macrophages and induced the cells to secrete pro-angiogenic growth factors, such as VEGF and platelet-derived growth factor BB (PDGF-BB). The heparin component subsequently sequestered these newly secreted growth factors in situ to promote blood vessel formation, thus obviating the need for any additional exogenous factors.^164^

In addition, hydrogels can serve as platforms for incorporating micro- or nanoparticles to enhance the in vivo stability of bioactive factors and control their delivery. For instance, SDF-1, a key member of the chemokine CXC family, is typically present in inflammation and/or hypoxia microenvironments and can mobilize various cell types, including lymphocytes, monocytes, neutrophils, megakaryocytes, and certain stem cells.^165,166^ While the local application of SDF-1 aims to direct transplanted cells towards injured sites post-implantation,^167^ the direct administration of SDF-1α is susceptible to enzymatic degradation and rapid diffusion away from the application sites.^168^ To overcome this, Zheng et al. utilized polydopamine nanoparticles as carriers for SDF-1, which were then incorporated into imidazole-modified gelatin methacrylate (GelMA) along with human amniotic MSCs (hAMSCs).^29^ The electrostatic forces and non-covalent bonds between polydopamine and SDF-1 enabled a prolonged release of SDF-1 with preserved bioactivity, effectively guiding hAMSCs to the injured sites. Furthermore, the inherent RGD peptide of GelMA and the imidazole groups promoted cell adhesion and neural differentiation, contributing to the physiological recovery of traumatic brain injury. Similarly, another study that incorporated SDF-1 into a nano-silicate-reinforced GelMA hydrogel achieved a prolonged 21-day release of SDF-1, yielding significant in vivo results with a healing rate over tenfold in rat calvaria defect models.^169^

The demonstrated success of hydrogels in delivering and immobilizing cytokines and growth factors exemplifies their potential to enhance cell therapy outcomes. Importantly, hydrogels loaded with these molecules offer benefits beyond supporting transplanted cells; they can also recruit endogenous cells from distant sites and facilitate their functional integration. An example includes an alginate-based hydrogel loaded with SDF-1 and BMP-2, which exhibited the capacity to attract and capture host stem and progenitor cells to the gel’s implantation site under ultrasound irradiation.^170^ Such strategies hold particular significance for conditions lacking accessible or sufficient cell sources. Of course, the potential applications of well-engineered hydrogel systems extend further, considering their ability for more precise, spatiotemporal release of bioactive factors^171^ and their highly tunable physical properties,^172^ which will be further discussed in the corresponding sections of this review.

##### Microenvironmental stress

Transplanted cells are often dispatched to diseased regions where they encounter harsh conditions and various adverse microenvironment stress, including hypoxia, oxidative stress, acidic conditions, inappropriated osmotic pressure, nutrient scarcity, and pathogenic invasion. These stressors can disrupt cellular homeostasis and cause cellular damage, such as DNA damage, mitochondrial dysfunction, endoplasmic reticulum stress, and induced autophagy,^173,174^ which could substantially impair the therapeutic potential of transplanted cells. More severely, prolonged and intense exposure to these adverse conditions can directly lead to cell death.^175^ For instance, the TME is characterized by stressful conditions like hypoxia, limited nutrient availability, and pH fluctuations, which can trigger autophagic cell death or apoptosis of transplanted cells.^176–178^ In areas of severe trauma or infection, inflammation-induced oxidative stress and pathogenic stimuli can result in various forms of cell death, such as apoptosis, necrosis, pyroptosis, and ferroptosis.^179,180^ Consequently, these could lead straight to engraftment failure.

In such challenging circumstances, integrating hydrogel systems, with rational structure and functional designs, into cell-based therapies could be a facile but effective materials-driven approach to mitigate these issues. For one thing, hydrogels can be tailored with disease-specific sensitive elements (e.g., in response to ROS, acid, or protease), acting as an “absorption sink” to neutralize harmful molecules to safeguard transplanted cells from adverse stimuli and thus increase their survival possibility. For instance, stem cell replacement is currently a promising strategy for repairing tissues that lack self-regeneration capabilities, yet overproduced ROS following excessive inflammatory responses post-injury often inflict severe oxidative stress on transplanted stem cells, leading to fruitless transplantation and regeneration failure. To address this issue, Ying and coworker combined an antioxidant polymer N^1^-(4-boronobenzyl)-N^3^-(4-boronophenyl)-N^1^, N^1^, N^3^, N^3^-tetramethylpropane-1,3-diaminium (TPA) with Laponite to form a shear-thinning, ROS-scavenging hydrogel via electrostatic interactions for treating traumatic spinal cord injury (SCI).^181^ Owing to ROS removal, this system effectively reduced ferroptosis in transplanted dental pulp stem cells by preventing lipid peroxidation, ultimately achieving axon regeneration and functional recovery from SCI. Another similar attempt utilized a ROS-cleavable thioketal-containing polymer crosslinking methacrylate hyaluronic acid to develop a ROS-responsive and -scavenging hydrogel, encapsulating BMSCs for SCI treatment.^182^ When applied in a rat SCI model, this hydrogel system exhibited potent antioxidation and protective effects, markedly reducing apoptosis at the lesion sites, and converting the hostile oxidative microenvironment into a regenerative one.

Aside from oxidative stress, disruptions in ionic balance can compromise the performance of transplanted cells, leading to protein inactivation, abnormal ion channel activity, metabolic irregularities, and potentially cell death.^183^ Like the acidic microenvironment typically at tumor or inflammation sites, such a condition increase free calcium ions (Ca^2+^), further elevating intracellular Ca^2+^ levels and consequently affecting cell migration, differentiation, cytoskeletal remodeling, and apoptosis.^184,185^ In this scenario, pH-responsive hydrogels offer a solution to neutralize the harmful acidic byproducts of inflammation or tumor anaerobic metabolism. Cheng et al. developed a pH-responsive hybrid hydrogel that incorporated an acid-scavenger—mesoporous bioactive glass nanoparticles—into a gelatin and oxidized starch matrix *via* hydrogen bond.^113^ This hydrogel rapidly neutralized tumor acidity and elevated the TME pH, which significantly enhanced the therapeutic effect of NK cell infusion, contributing to the prevention of hepatocellular carcinoma recurrence in a mouse model. Furthermore, hydrogels can sequester excess free Ca^2+^ in the microenvironment by employing Ca^2+^ as a crosslinker in various gel formulations, such as alginate-based,^186,187^ κ-Carrageenan,^188^ and specific synthetic polymer hydrogels,^189^ highlighting their potential in regulating ion balance to optimize cell therapy outcomes.

For another, hydrogels can exploit their versatile loading capabilities to serve as continuous, supplementary reservoirs, replenishing oxygen, antibacterial, or other therapeutic agents to reverse adverse microenvironments. For example, as most transplanted cells heavily rely on oxygen for energy metabolism, oxygen scarcity in hypoxic environments, such as ischemic or inflammatory sites, can lead to reduced energy production and potentially induce cellular stress or inactivation. In response, hydrogels laden with oxygen carriers like hemoglobin or fluorinated compounds can transport oxygen directly to lesion sites to alleviate hypoxia and prevent hypoxia-induced cell death.^190,191^ An illustrative example includes an alginate-based hydrogel co-encapsulating CAR-T cells, hemoglobin, and IL-15 for antitumor treatment, where hemoglobin functioned as an oxygen reservoir, continuously delivering oxygen to counteract the hypoxia TME.^192^ This action, coupled with the synergistic effect of IL-15, notably enhanced CAR-T cell survival, persistence, and anti-tumor potency in a mouse subcutaneous tumor model. Likewise, Niu and coworkers conjugated an oxygen carrier, perfluorocarbon, into an N-isopropylacrylamide-based hydrogel aimed at ischemic tissue regeneration.^193^ This design demonstrated high oxygen retention and rapid gelation at body temperature, effectively supporting the survival and proliferation of encapsulated MSCs’ under a hypoxia condition for over 14 days. An alternative strategy to combat hypoxia and boost cell survival involves incorporating catalase into the hydrogel system, although this approach is limited to ROS-accumulated areas, as oxygen generation relies on the catalase-mediated H_2_O_2_ decomposition.^194,195^

The applications of hydrogel-based strategies against pathogenic infections and their biomedical prospects have been extensively reviewed.^196–198^ In essence, hydrogels can transport bioactive agents including bacteriophages, antimicrobial peptides, enzymes, antibiotics, or metal nanoparticles (e.g., silver-, gold-, zinc oxide-) for antibacterial purposes. Alternatively, hydrogels themselves can be formulated from inherently antibacterial components, such as polycationic polymers, antimicrobial peptide polymers, or anti-fouling zwitterionic polymers. Nonetheless, it’s crucial to recognize that the potent antimicrobial activity of these materials may be along with the incompatibility and toxicity risks to transplanted cells. Thus, integrating antibacterial functionalities into hydrogels necessitates careful considerations regarding the dosage and proportion of these agents/polymers to balance cytocompatibility with antimicrobial efficacy.

Collectively, these instances affirm the critical role of strategically designed hydrogel systems in supporting transplanted cells amidst adverse microenvironment stressors. Whether through the introduction of functional moieties into the hydrogel network or leveraging the gel’s carrier properties, this strategy can facilitate microenvironment reshaping, creating conducive conditions for cell survival, thereby achieving satisfactory cellular therapeutic outcomes at lesion sites.

##### Cell–matrix interactions

The interactions between cells and the ECM are multifaceted and significantly influence a broad array of physiological and pathological processes, such as histogenesis, aging to disease progression. Beyond its role in providing structural support, the ECM provides a variety of biochemical signals from its components, such as collagens, proteoglycans, glycoproteins, and elastins, alongside various biophysical cues, including matrix stiffness, viscoelasticity, and geometrical cues. These elements are crucial in regulating cellular behaviors and determining cell fate,^199^ such as how ECM stiffness significantly affects cell growth, adhesion, and differentiation, and how gradients in ECM components guide cell migration and functionality. At the same time, cellular responses can chemically and mechanically remodel the ECM, which dynamically undergoes deposition, remodeling, and degradation to maintain tissue homeostasis and response to stress.^200,201^ At its core, the interplay between cells and the matrix underpins the foundation for biomaterial-based cell therapy, ranging from sustaining cell survival to modulating the functionality of both transplanted and endogenous cells. Therefore, a comprehensive understanding of the bidirectional interactions between cells and ECM, encompassing their chemical composition, physical properties, and finer structural aspects, is of paramount importance for the advancement of hydrogel designs as an artificial ECM to support cell therapy. This review will delve into this topic in detail in the subsequent part (“Design considerations on gel’s physicochemical properties for cell therapy”), focusing on the design considerations of hydrogels in cell therapy settings.

##### Involved cellular signal transduction pathways

From a mechanism perspective, the aforementioned external stimuli ultimately influence cellular processes through the activation of cell surface receptors, thereby triggering changes in intracellular signaling cascades and subsequently modulating gene transcription and expression. This subsection aims to elucidate the mechanisms underlying intracellular signaling pathways, including the sources of pathway activation, the molecular constituents involved, and the resulting biological outcomes. We hope to offer an enhanced understanding of the factors influencing the success or failure of cellular therapies.

Cells respond to external stimuli through various signaling pathways, such as phosphoinositide 3-kinase/protein kinase B (PI3K/AKT),^202^ mitogen-activated protein kinase/extracellular signal-regulated kinase (MAPK/ERK),^203^ nuclear factor kappa B (NF-κB),^204^ receptor tyrosine kinases (RTKs),^205^ Wnt/β-catenin signaling.^206^ For example, the cytokine IL-2 enhances the expression of CXCR3 in CAR-T cells via the PI3K/AKT pathway, thereby increasing their in situ chemotaxis towards cancer cells.^207^ SDF-1α binds specifically to the CXCR4 receptors on the surface of MSCs, influencing key proteins in the mTOR signaling pathway within MSCs, which are responsible for cell migration and the homing signal pathway of stem cells.^208^ Exposing cells to hypoxic environments activates several signaling pathways, including the HIF-1α pathway, NF-κB pathway, and p53 pathway, leading to the initiation of apoptosis and ultimately resulting in cell death.^209^

**Fig. 4:**
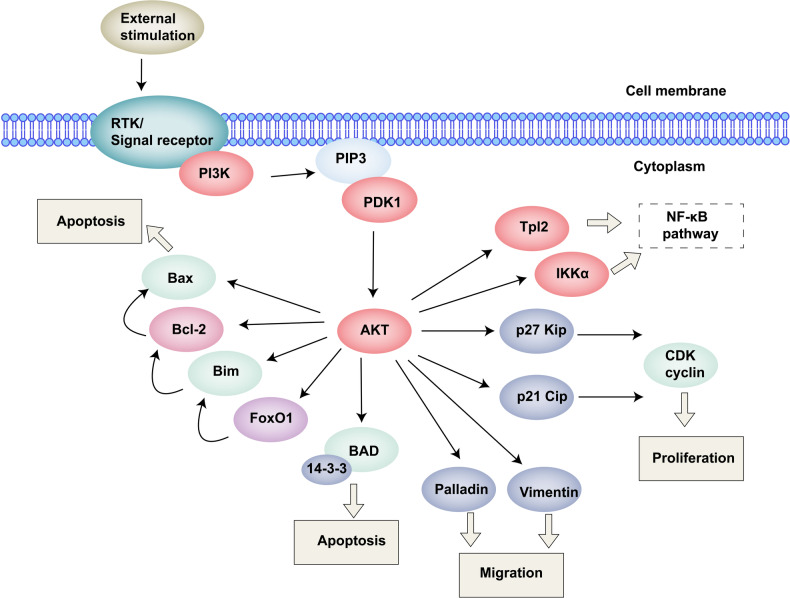
The cellular behavior mediated by the AKT pathway. The PI3K/Akt signaling pathway significantly influences cell survival, proliferation, metabolism, and other cellular processes. Upon reception of external stimuli, various surface receptors (e.g., RTKs, ion channel receptors, G protein-coupled receptors) can activate and recruit PI3K to the plasma membrane, which then phosphorylates PIP2 into PIP3. PIP3 subsequently recruits PDK1 to the cell membrane, leading to the phosphorylation and activation of Akt. This activation affects the expression or phosphorylation of downstream proteins (e.g., vimentin, p21, p27, Bax, etc.), ultimately influencing cellular outcomes, including apoptosis, migration, and proliferation, among others. Information is collected from published work^215–218,221,825^ and Cell Signaling Technology (https://www.cellsignal.cn/). Adobe Illustrator was used to generate this figure. (Abbreviations: PI3K Phosphoinositide 3-kinase, PDK1 Protein kinase D1, AKT Protein kinase B, Bax Bcl-2-associated X protein, Bcl-2 B-cell lymphoma 2, Bim Bcl-2-interacting mediator of cell death, FoxO1 Forkhead box O1, Bad Bcl-2-associated death promoter, p21 Cip Cyclin-dependent kinase inhibitor 1, p27 Kip Cyclin-dependent kinase inhibitor 1B)

While cellular signaling pathways exhibit crosstalk and overlap, the PI3K/AKT signaling pathway is notably instrumental in regulating cell survival, proliferation, metabolism, and other cellular functions.^210^ The pivotal role of the PI3K/AKT pathway in specific cellular biological processes is substantial and therefore merits detailed discussion here (Fig. 4).

Once receiving external stimuli, the respective surface receptors (e.g., RTKs, ion channel receptors, G protein-coupled receptors) can be activated and recruit PI3K to the plasma membrane, then phosphorylating phosphatidylinositol-4,5-bisphosphate (PIP2) into phosphatidylinositol-3,4,5-trisphosphate (PIP3).^211–213^ PIP3 further recruits protein kinase D1 (PDK1) to the cell membrane, which then phosphorylates and activates AKT.^214^ This activation can lead to the phosphorylation of p21 and p27, typically resulting in their retention within the cytoplasm. Such retention reduces their inhibitory effects on cyclin-dependent kinases (CDKs) within the nucleus, thereby promoting CDK activity and culminating in cell cycle progression and increased proliferation.^211,215^ Furthermore, activated AKT can contribute to cellular survival by inhibiting the pro-apoptotic activities of the Bcl-2 family proteins (e.g., Bim, Bax, and Bad), thereby reducing the occurrence of apoptosis.^211^ Moreover, AKT can also influence the expressions of proteins involved in cell migration and invasion, such as palladin^216^ and vimentin,^217^ highlighting its significant role in the regulation of transplant cell functions.

The PI3K/AKT/Forkhead Box O1/3 (FoxO1/3) pathway governs the dynamics of T helper cell 1 (Th1)-Tregs, a T cell subset exhibiting Th1-like attributes. Research indicated that isolated Tregs can be induced to adopt the Th1-Treg phenotype in the presence of PI3K or AKT1 activators, or through the inhibition of PTEN or FoxO1/3.^218^ This suggests that the PI3K/AKT signaling axis serves as a critical regulatory mechanism closely associated with the functionality and stability of Treg cells.^219^ In the context of corneal endothelial injury in a rabbit model, it was observed that corneal endothelial cells treated with PI3K/AKT activators exhibited improved proliferation and migration capabilities, successfully restoring normal corneal thickness.^220^ Moreover, AKT’s role can extend to modulating the NF-κB signaling pathway by phosphorylating Inhibitory Kappa B Kinase α (IKKα) and tumor progression locus 2 (Tpl2),^221^ which in turn enhances CAR-T cell proliferation and anti-apoptotic activities.^222^ These insights underscore the complex interplay among these signaling pathways in orchestrating a range of cellular behaviors and processes.

In summary, the factors that affect the efficacy of cell therapy are complex and intertwined, primarily involving cytokines and growth factors, microenvironment stress, and cell-matrix interactions. Targeted interventions in these areas, particularly through strategies driven by hydrogel-based biomaterial, have the potential to significantly enhance cell therapy outcomes. Furthermore, a detailed examination of the intrinsic signaling pathway mechanisms of these factors can enhance our understanding of the determinants of cellular behavior and phenotype, which contributes to developing more effective and innovative hydrogel-based approaches in cell therapy.

#### Design considerations on gel’s physicochemical properties for cell therapy

Upon the encapsulation of cells within hydrogels, these materials serve as an extracellular microenvironment surrogate, where their material composition, structure, and physicochemical properties significantly influence various cellular processes such as adhesion, survival, proliferation, differentiation, activation, and migration, thereby regulating cell behavior. The mechanical microenvironment offered by hydrogels, for example, can exert a profound influence on cell mechanotransduction, which involves the cellular interpretation of mechanical signals from the hydrogel matrix. This process initiates intracellular structural reorganizations (e.g., alterations in the cytoskeleton and nuclear skeleton) that induce cell morphological changes and functional adaptations. Additionally, the mesh size, porosity, and degradation kinetics of hydrogels are critical in modulating cell attachment, nutrient exchanges, and release patterns.

Given the design considerations of hydrogels, the basic principle of these artificial ECM analogs is ensuring biosafety and cytocompatibility, which includes not only their components and modifications but also the gelation conditions, catalysts, and potential by-products. Moreover, considering the inherent disparities between biopolymers and the natural ECM, the design of hydrogels aims to emulate the natural ECM as closely as possible, which can be achieved by tailoring their structural properties and incorporating biological cues to optimal cellular performance within these matrices. The key design parameters have been discussed as follows.

##### Chemical composition

The materials commonly utilized in the fabrication of cell therapy hydrogels are primarily categorized into natural-derived biopolymers and synthetic polymers. Natural-derived hydrogels are characterized by their inherent bioactivity and capability to closely mimic the biological cues of the natural ECM, making them highly conducive to supporting cell growth, adhesion, and specific differentiation pathways. Despite their intrinsic advantages, the application of natural hydrogel is constrained by their limited tunability, suboptimal mechanical properties, and the immunogenic risk of xenogeneic components, which can lead to batch-to-batch variability and challenges in achieving reproducibility on a large scale. Conversely, synthetic hydrogels present defined compositions, enhanced mechanical strength, and extensive tunability, allowing precise and independent control of their chemical and mechanical properties. However, to closely mimic the natural ECM’s functionality, synthetic hydrogels often necessitate the incorporation of chemical modifications, such as the introduction of functional binding motifs and other bioactive elements. To overcome the limitations inherent to both natural-derived and synthetic polymers, future advancements may focus on the development of hybrid hydrogels or interpenetrating polymer network (IPN) hydrogels.^223^ These innovative strategies aim to combine the advantages of both hydrogel types or other structures to a create hierarchical structure with sophisticated functionalities and precise control mechanisms for the behavior of transplanted cells. In this context, we delineate ten commonly used polymer types in the fabrication of hydrogels for cell therapy, discussing their physicochemical properties and potential application scenarios. Detailed examples of these applications are summarized in Table 2.

**Table 2:** Characteristics of functional hydrogels and their roles in cell-based applications

Chemical composition			Cross-linking methods	Cell interactive motifs	Mechanical properties	Mesh or pore size	Degradability		Cell types	Applications	Functions	Ref
Based hydrogels	Crosslinked hydrogel components	Other functional moieties					Time	Conditions				
Alginate (Alg)	Alg	CPA	Physical (Calcium-Alg coordination)	-	E: 1.2 GPa	-	-	-	hUCMSCs	Bone regeneration	CPA maintained the injectability of the paste and enhanced the mechanical strength	^226^
	Alg-Fibrin	-	Physical (Calcium-Alg coordination, Interpenetrating networks)	Fibrin	-	12 ~23 nm	24 h to days	Alginate lyase	-hMSCs -hECs	Formation of a capillary network	Fibrin exhibited adhesive properties	^794^
	Alg	PLGA-AL	Physical (Calcium-Alg coordination)	-	-		24 h to days	Alginate lyase (β-elimination reaction)	rNPCs	Neural tissue regeneration	PLGA-AL tuned the gel’s degradation rate	^795^
	-Alg -RGD peptide	Alginate lyase	-Covalent -Physical	RGD peptide	-	-	24 h to days	Alginate lyase (β-elimination reaction)	hOECs	Revascularization (Chick chorioallantoic membrane model)	Alginate lyase accelerated the gel’s degradation rate	^796^
Hyaluronic acid (HA)	-HA -Methacrylic anhydride -MBA	-P-aPD-L1 -IL-15	Covalent (Radical polymerization)	IL-15	-	-	Weeks	Hyaluronidase	CAR-T cells	Inhibition of post-surgery tumor recurrence (mouse melanoma resection model)	-P-aPD-L1 blocked the PD-1/PD-L1 pathway -IL-15 maintained the activity and proliferation of CAR-T cells	^28^
	-HA -PBA -Sodium periodate -Methacrylic anhydride	-	Covalent (Radical polymerization)	PBA	E: 2.6 ~23.2 kPa	-	-	-	rbCCs	Cartilage regeneration	PBA enhanced the interaction of chondrocytes and hydrogel, promoting cell adhesion and aggregation through filopodia	^797^
	-HA -Methacrylate	-	Covalent (Radical polymerization)	-	-	-	Days to weeks	-	hMSCs	Endometrial regeneration (Endometrial injury rat model)	-	^798^
Chitosan (CS)	-CS	-	-		-	50~500 μm	Weeks	-	hT cells	Cancer immunotherapy	-	^799^
	-CS -Dextran -β-GP	-	Physical (Electrostatic attraction, hydrogen bond interactions)	-	-	18.5~25.4 μm	70% in 4 weeks	Enzyme	hUCMSCs	Myocardial infarction therapy	β -GP reduced chitosan chain polarity, making the hydrogel milder and less cytotoxic	^800^
	-CS -Glyoxal -Col I	-	Physical (Electrostatic attraction)	Col I	-	-	-	-	hBMSCs	Bone regeneration	-Glyoxal enhanced the mechanical hardness of the hydrogel -β -GP enhanced hydrogel stability	^801^
Collagen (Col)	-Col I -Methacrylate	-	Covalent (Radical polymerization)	Col I	-	-	20.9%~78.6% at 12 h	Collagenase	-rbMSCs -rbCCs	Chondrogenesis	Methacrylate-modified gelatin reduced the gel’s degradation rate	^802^
	Col I	-	-	CTX	E: 170–227 Pa	-	-	-	-hADSCs -hUVECs	Regenerative applications	CTX regulated encapsulated cell fate (apoptosis, adhesion, and migration)	^803^
	-Col I -Alg	PCL/Gel nanofibers	Physical (Calcium-Alg coordination)	RGD peptide	E: 0.0093~0.25 MPa	-	30% in 7 days	-	rADSCs	Wound dressing (rat wound model)	Nanofibers were used as coverage of the scaffold to improve the mechanical strength of the hydrogel	^804^
Gelatin (Gel)	-Gel -Methacrylate	EGCG-EF	Covalent (Radical polymerization)	-	-	458 ~519 μm^2^ (pore area)	-	-	-hADSCs -hDFBs -hUVECs	Tissue engineering applications	EGCG cleared the free radicals	^805^
	-Gel -YIGSR peptide -QK peptide	-	Covalent (Esterification reaction)	-YIGSR peptide -QK peptide	-	-	-	-	-hUVECs -hGEnCs	Tissue engineering applications	-QK peptide stimulated endothelial cell growth -YIGSR peptide promoted cell adhesion and migration	^806^
	-Gel -Methacrylate -Alg	-	Covalent (Radical polymerization)	-	E: 6.0~11.0 kPa	-	Days to weeks	Enzyme	hUVECs	Neovascularization (Hind-limb ischemia mouse model)	-	^807^
Silk fibroin (SF)	-SF -Col I	-	Physical (Interpenetrating networks)	-	-	-	-	-	hMSCs	Tissue engineering applications	-SF enhanced the mechanical properties -Collagen provided cell adhesion sites	^808^
	-SF -SF-TA -Cyclic RGD peptide	-	Covalent (Phenol oxidation reaction)	Cyclic RGD peptide	E: ~300 kPa	20 ~ 35 nm	Days to weeks	Protease	hMSCs	Tissue engineering applications	SF-TA enhanced the hydrogel mechanical stability and resistance to enzymatic degradation	^809^
	-SF -G-TA	-	Covalent (Dityrosine crosslinking)	RGD sequence	E: ~300 kPa	20 ~ 35 nm	Days to weeks	Protease	hMSCs	Tissue engineering applications	G-TA enhanced the hydrogel mechanical stability and resistance to enzymatic degradation	^809^
DNA	-DNA -SilMA -RGD peptide	-	-Physical -Covalent (Radical polymerization)	RGD peptide	-	527.5 ± 25.55 μm	Weeks	Protease	rBMSCs	Cartilage repair (Cartilage defect rat model)	The DNA constrained the SF to increase its β -fold content, allowing for the precise tuning of the surface stiffness of the hydrogels	^810^
	-DNA -IKVAV peptide - Polyacrylamide	-	Covalent (sulfhydryl-MBS crosslinking chemistry)	IKVAV peptide	-	-	-	-	SH-SY5Y cells	Neuronal regeneration	IKVAV peptide enhanced nerve cell attachment and differentiation	^246^
	-DNA -Tetraethylene glycol	-	Physical	-	-	<10 μm	24 h	DNase I	-A549 cells -MCF-7 cells -HEK 293 cells	3D cell culture	**-**	^811^
Poly (ethylene glycol) (PEG)	-PEG-LA-DM -HA	-	-Physical (Interpenetrating networks) -Covalent (Radical polymerization)	HA	E: 180-230 kPa	-	Weeks	Hydrolysis	bCCs	Cartilage tissue regeneration	-LA promoted hydrogel degradation and macroscopic tissue deposition -DM polymerized in situ and bore in vivo forces	^812^
	-PEG -PCL	-	Covalent (acid chloride/alcohol chemistry reaction)	-	E: 2.7~7.1 MPa	-	Weeks	-Hydrolysis -Enzyme	hCECs	Corneal endothelial regeneration (Impaired ovine corneas model)	PCL was covalently incorporated into hydrogel to provide strong tensile properties	^813^
	PEG-g-CS	-	Dynamic covalent (Schiff base reaction)	-	-	0.677±0.049 μm	-	-	Human T cells	Localized glioblastoma immunotherapy	^-^	^27^
Polyvinyl alcohol (PVA)	-PVA -Gel	-	Physical	RGD sequence	E: 3.8 ~5.2 MPa	-	-	-	-NIH3T3 cells -HeLa cells	Study cell behavior and function	-	^16^
	-PVA -CS	HAp	Physical	HAp	E: 109~248 kPa	-	-	Heat	rBMSCs	Bone repair (Bone defect rabbit model)	HAp enhanced the hydrogel-bone interface binding capacity	^257^
	-PVA -HA	-	Physical	-	-	-	-	-	hBMSCs	Study cell behavior and function	-	^814^
Polyacrylamide (Paam)	-PAAm -DNA -BIS	-	-Covalent -Physical (Interpenetrating networks)	-	-	-	-	-	HEK cells	Study cell behavior and function	DNA and BIS enhanced the hydrogel mechanical stability	^815^
	-PAAm -Alg	-	Covalent (Radical polymerization)	-	-	50-100 μm (Porosity: 91%)	-	-	hBMSCs	Tissue engineering applications	The entangled network of PAAm and Alg polymer chains bringed a higher mechanical strength	^816^
	PAAm	-	Covalent (Radical polymerization)	-	E: 0.67-44 kPa	-	-	Hydrolysis	hPOD	Developing the renal chip model	-	^817^

*E* elastic modulus, *Alg* alginate, *Col* collagen, *Gel* gelatin, *RGD* Arg-Gly-Asp, *HA* hyaluronic acid, *CS* chitosan, *PBA* phenylboronic acid, *SF-TA* tyramine-substituted silk fibroin, *G-TA* tyramine-substituted gelatin, *β-GP* β-glycerophosphate, *SilMA* methacrylate groups-modified silk fibroin, *IKVAV* Ile-Lys-Val-Ala-Val, *PEG* poly(ethylene glycol), *PEG-LA-DM* poly(ethylene glycol)-lactic acid-dimethacrylate, *PEG-g-CS* poly(ethylene glycol)-*g*-chitosan, *PVA* polyvinyl alcohol, PAAm polyacrylamide, *BIS* -N,N-bisacrylamide, SF silk fibroin, CPA phosphate, PLGA-AL poly(lactide-co-glycolide) microspheres loaded with the enzyme alginate lyase, PCL/Gel polycaprolactone/gelatin nanofibers, P-aPD-L1 aPDL1-conjuated platelets, *EGCG-EF* nanofiber particles coated with epigallocatechin-gallate, HAp hydroxyapatite, *CTX* carboxy-terminal telopeptide residues in collagen, *APS* ammonium persulfate, *sulfo-MBS* m-maleimidobenzoyl-N-hydroxysulfosuccinimide ester

*Alginate-based hydrogels*: Alginate hydrogels are typically composed of natural alginates or their derivatives, constituting a linear anionic polysaccharide structure. This polysaccharide comprises blocks of mannuronic acid (M) and guluronic acid (G), wherein the ratio of M to G segments profoundly impacts its properties and functionalities. Alginate with higher M content exhibits immunogenicity and more effectively induces cytokine production.^224^ Conversely, the structural integrity of G blocks confers folding and rigidity, thereby increasing the stiffness of alginate molecular chains.^224^ The linear anionic polysaccharide structure of these hydrogels potentially offers additional cell adhesion sites, facilitating cell attachment and proliferation.^225^ Prepared via ionotropic gelation, alginate hydrogels can hold bioactive substances such as calcium and barium, which could influence cell growth and differentiation.^224,225^ For instance, alginate-calcium hydrogels are often applied for bone tissue regeneration. Calcium phosphate minerals, in particular, possess preferred bioactivity for cell adhesion and osteoblastic phenotype expression. Based on that, Zhao et al. developed an injectable, mechanically robust stem cell-calcium phosphate cement-alginate scaffold structure, which successfully promoted the osteogenic differentiation, and bone mineral synthesis of the encapsulated human umbilical cord MSCs (hUCMSCs) while well preserving their viability.^226^

*Hyaluronic acid (HA)-based hydrogels*: HA, a polysaccharide naturally occurring in human tissues, constitutes a major component of the ECM.^227^ Its high molecular weight and remarkable hydration capacity render this material high hydrophilicity and moisturizing properties, making it well-suited as hydrogel wound dressings.^228^ Additionally, hyaluronic acid plays a crucial role intracellularly, interacting with proteoglycans and type II collagen, and modulating cell adhesion, migration, proliferation, and differentiation through interactions with CD44 and receptor for hyaluronan-mediated motility (RHAMM). These facts showcase HA’s potential of involved in many cellular processes such as inflammation, wound healing, tissue development, morphogenesis, tumor progression, and metastasis.^227,229^ To meet clinical demands, HA can be modified through addition reactions, condensation reactions, and photochemically induced free radical polymerization (e.g., thiolation, chloroacetylation, dihydrazide modification, aldehyde modification, tyramine modification, and Huisgen cycloaddition) to generate its derivatives with tailored structures and mechanical properties suitable for specific usages, especially for tissue repair and regeneration.^230^ For example, an in-situ gelling hydrogel composed of amino-functionalized HA-derivatives and diethylene glycol divinyl ether functionalized chitosan has been reported effectively supporting the survival and proliferation of the encapsulated chondrocytes over 28 days, thereby promoting cartilage regeneration.^229^

*Chitosan-based hydrogels*: Chitosan is a cationic polysaccharide recognized for its antibacterial and anticoagulant activity, making it a popular choice in many medical applications. The functional carboxyl and amino groups in chitosan facilitate the fabrication of versatile chitosan-based hydrogels.^231^ Through chelation, chitosan can effectively coordinate with various metal ions,^232^ forming the basis for metal-chitosan composite hydrogels. For instance, the inclusion of Cu^2+^ can create copper-based antimicrobial composites.^233^ Calcium ion-crosslinked alginate-chitosan hydrogels can demonstrate bactericidal properties against pathogens like *Pseudomonas aeruginosa* and *Staphylococcus aureus*. These properties are particularly advantageous in complex wound management when combined with stem cell-based therapies.^234^ Additionally, such calcium ion-chitosan composites also hold great potential in bone repair,^232^ exemplified by a novel chitosan/hydroxyapatite hydrogel that significantly enhanced the proliferation and differentiation of MSCs into osteoblasts.^235^

*Collagen-based hydrogels*: Collagen is vital for maintaining the structure, morphology, and mechanical properties of biological tissues, while also possessing specific biological functions to regulate cellular proliferation, migration, and differentiation by interacting with cell surface receptors.^236^ Specifically, Col2, a type of collagen, contains peptide sequences that bind to collagen receptors on human adipose-derived MSCs (hADMSCs), activating the MAPK/ERK signaling pathway and participating in Smad signaling, thereby regulating cell differentiation.^237^ Recent research has shown that a type of Col2 hydrogel can effectively repair critical-sized osteochondral defects (diameter of 1.5 mm) induced by knee joint osteoarthritis in rats.^238^ This hydrogel inhibited the TGF-β-Smad1/5/8 signaling pathway, thus facilitating the healing of lesions that would not naturally regenerate.

*Gelatin-based hydrogels*: Gelatin is a product derived from partial denaturation and hydrolysis of collagen, which presents excellent tissue compatibility, biodegradability, and hemostatic properties.^239^ Gelatin hydrogels provide abundant cell adhesion sites that promote cell migration and tissue remodeling.^240^ Despite these advantages, the properties of gelation alone are not always sufficient for demanding biomimetic scaffolds, particularly those designed for complex tissues like cartilage. To address this, biofunctionalization techniques are often applied to enhance gelatin-based hydrogels. For example, a modified gelatin-based hydrogel, incorporating chemically modified ε-poly-L-lysine (EPL) and mechanically modified phenylboronic acid (PBA), has been developed.^241^ The introduction of EPL imparted a positive surface charge to the gelatin-based hydrogel, enhancing the adsorption of negatively charged proteoglycans, thereby creating a conducive microenvironment for chondrocyte viability. Furthermore, the addition of PBA introduces dynamic covalent bonds, which conferred stress-relaxation properties to the gel, making it more suited for modulating the mechanical behavior of chondrocytes.^241^ These modifications enhanced the viability and differentiation of stem cells into chondrocytes and significantly contributed to the repair of cartilage defects.

*Silk fibroin-based hydrogels*: Silk fibroin, a member of the collagen protein family, is one of the crucial structural proteins in connective tissues and exhibits substantial biological activity. These properties make silk fibroin hydrogels particularly useful in regenerative medicine. For example, a silk fibroin-based hydrogel has been reported to promote the migration of corneal keratocytes and significantly enhance the proliferation and adhesion of human corneal stromal cells, thereby contributing to the reconstruction and healing of corneal tissues.^242^

*DNA-based hydrogels*: DNA hydrogels leverage the unique genetic functionality, specificity in hybridization, precise molecular recognition, adjustable multifunctionality, and programmability of DNA,^243^ positioning it as an ideal molecule to bridge biological and material sciences.^244^ As a highly negatively charged polymer, DNA readily integrates with other functional materials, such as peptides, proteins, and synthetic organic polymers, enriching the hydrogel functionality. While single-component DNA hydrogels are effective, their high production costs limit large-scale applications. Introducing other polymers or nanomaterials into DNA hydrogels can improve their mechanical properties, expand functionalities, and reduce costs.^245^ Importantly, DNA hydrogels can support cell adhesion, growth, and diffusion, and to some extent regulate cell behavior,^246^ as evidenced by experiments showing increased cell area, receptor expression, endocytosis, and 3D migration in cells cultured on these hydrogels.^247^ Additionally, the adhesive properties of DNA are capable of reshaping cell membranes, enhancing the expression of cell surface receptors, and endocytosis mechanisms. Therefore, DNA hydrogels can be considered effective scaffold materials that mimic the natural extracellular matrix, filling the gap between ex vivo cultures and in vivo models.^247^

*Polyethylene glycol (PEG)-based hydrogels*: PEG hydrogels are synthesized through the copolymerization of monomers with complementary reactive groups, forming hydrogels with uniform network structures that maintain homogeneity even under equilibrium swelling conditions, a feature that underpins their exceptional mechanical properties.^248,249^ Despite their structural benefits, PEG hydrogels generally exhibit low cell adhesion due to the absence of active groups for cell interactions on their surfaces, possibly limiting their direct use in the context of cell-based therapies.^250^ On the other hand, this feature may contribute to reducing nonspecific interactions with cells. Nevertheless, their limited bioactivity on cells can be overcome by functionalizing PEG structures (linear, tetra-, hexa-, and octa-arm) with various groups such as diselenides, dithiols, RGD peptides.^250–253^ For instance, a PEG-peptide hydrogel synthesized by reacting tetra-arm PEG azide moieties with dipropargyl peptide demonstrated enhanced cellular interactions, as evidenced by significant expression of F-actin and phosphorylated focal adhesion kinase (pFAK) in human corneal epithelial cells cultured on them.^253^ This highlights the potential of PEG-based hydrogels, once biofunctionalized, can serve as effective 3D cell scaffolds for tissue engineering or other cell-based usages.

*Polyvinyl alcohol (PVA)-based hydrogels*: PVA hydrogels have garnered considerable attention in cell-based therapies due to their excellent biocompatibility, low friction coefficient, and optimal water retention.^254,255^ However, PVA hydrogels often fall short in mechanical robustness when employed as substitutes for load-bearing tissues like articular cartilage.^256^ To enhance their mechanical performance, PVA hydrogels can be modified by blending with other polymer networks, both natural (such as chitosan and collagen) and synthetic (like polyacrylamide, polyacrylic acid, polycaprolactone), which impart desired functionalities. Notably, a PVA-chitosan double network hydrogel has demonstrated superior mechanical properties, including tensile strength (0.24 MPa), elongation at break (286%), and high compressive strength (0.11 MPa at 60% strain rate),^257^ which are crucial for bone tissue engineering that requires robust mechanical support. Thus, strategic incorporation of toughening components may be a significant part of developing PVA-based hydrogels tailored to specific application requirements.

*Polyacrylamide (PAAm)-based hydrogels*: PAAm hydrogels are characterized by their rich amide (-CONH_2_) functional groups, which confer high hydrophilicity and swelling capacity—properties conducive to supporting cell survival and cellular processes in vivo.^258^ Their mechanical strength and toughness can be improved by many methods such as nanoparticle reinforcement, ion crosslinking, dipole-dipole interactions, and hydrophobic interactions.^259^ In addition, the introduction of natural polysaccharides, such as dextran, can increase the affinity between the gels and cells, thereby enhancing the hydrogel’s support and interaction with cells.^259^ However, their monomer (acrylamide) may present neurotoxin and other toxicity, so it is of paramount importance to remove any residual monomer from PAAm-based hydrogels to ensure their biocompatibility and biosafety.

*Other synthetic polymer hydrogels*: Hydrogels composed of synthetic polymers such as polyethylene oxide,^260^ polylactic acid,^261^ and polypropylene fumarate^262^ offer tunable mechanical strengths, but these materials typically suffer from poor biocompatibility and inadequate cell adhesion properties. Additionally, their rapid degradation and potential toxic byproducts can be significant drawbacks, further limiting their in vivo applications.^247,250,263^ Therefore, modification of the polymer composition and incorporation of bioactive molecules are necessary to mitigate their toxicity and improve their compatibility, thereby extending their utility in cell therapy fields.

##### Chemical modification

Synthetic polymer-based hydrogels, even some derived from natural sources, often exhibit insufficient bioactivity to mimic the natural ECM, necessitating modifications to augment their suitability for cell therapy applications. One of the most representative examples is the incorporation of cell-adhesion peptides that can bind to cellular integrin receptors,^264^ thus establishing a connective interface between transplanted cells and the hydrogel matrix. This interaction is particularly crucial for anchorage-dependent cells such as epithelial cells, fibroblasts, and certain stem cells (e.g., MSCs and PSCs), to support their survival, growth, and functionality.^265^ Without such interactions, those cells would undergo Anoikis—a form of apoptotic death induced by inadequate or inappropriate cell attachment and cell-matrix communication.^266^ Currently, the most commonly used (~90%) cell-adhesion peptides are those containing the Arg-Gly-Asp (RGD) motif, derived from fibronectin, which has been shown to significantly enhance cell viability and functionality when embedded within hydrogels.^267^ For instance, Shu et al. demonstrated that modification of a HA/PEG-based hydrogel with RGD-containing peptides notably improved fibroblast attachment, dispersal, and proliferation, with a noted dependence on the concentration of the RGD peptides.^268^ Other cell-adhesion peptides, including IKVAV and YIGSR from laminin, as well as GFOGER and DGEA from collagen, have also been incorporated in hydrogel formulations.^269^ Importantly, while certain cells, such as anchorage-independent cells like HSCs and most hematopoietic system-derived immune cells, do not require cell-matrix contact for viability, the incorporation of these adhesive motifs can still facilitate their traction-based mechanosensing processes and response to extracellular mechanical cues.^270^

Other modification strategies for hydrogel enhancement generally focus on equipping gels with specific bioactive functions, improving their mechanical properties, as well as integrating responsive degradability (as discussed in “Responsive design of hydrogels”). For instance, Chen et al. developed a chitosan-based hydrogel, functionalized with methacrylic anhydride and phosphocreatine.^271^ The phosphate groups not merely provided binding sites for the incorporation of MgO NPs via metal−ligand supramolecular interactions—thereby enhancing the gel’s mechanical performance; they also facilitated the controlled release of Mg^2+^ to exert its osteogenic and angiogenic functions, thus presenting significant in vivo bone regeneration in a rat calvarial defect model. Similarly, Hamai et al. demonstrated that the addition of sulfonic acid groups and Ca^2+^ into a hydrogel system markedly improved its hydroxyapatite formation capabilities, making it suitable for bone tissue engineering applications.^272^ Hydrophobic functional groups like tert-butyl and fluorine, when being tethered in hydrogels, have been found to enhance the adipogenic marker peroxisome proliferator-activated receptor gamma (PPARG), thereby facilitating adipogenic differentiation of hMSCs.^273^ Given the extensive coverage of this topic in previous reviews,^274,275^ this review refrains from a detailed discussion on this aspect.

##### Crosslinking methods

The methods for crosslinking hydrogels can typically be categorized into three types: covalent crosslinking, dynamic covalent crosslinking, and physical crosslinking. Covalently crosslinked hydrogels, which form irreversible bonds between polymer chains, exhibit chemically stable and maintain matrix properties over time. These hydrogels present superior mechanical strength and a wide range of achievable mechanical properties, making them suitable for mechanobiology studies, particularly regarding the relationship between matrix elasticity or modulus and cell behaviors.^269^ An established strategy for developing covalently crosslinked hydrogels is based on photo-initiated radical polymerization, allowing for a tunable gelation rate and precise spatiotemporal control of the gelling process.^276^ However, the cytotoxicity caused by photo-initiators and free radicals limits their in vivo applications, such as potential DNA damage to cells from UV light exposure during gelation.^277^ Given that, there is increasing attention on click chemistry-based covalent crosslinking methods, especially bio-orthogonal reactions such as strain-promoted azide–alkyne cycloaddition, inverse-electron demand Diels–Alder and Staudinger Ligation.^278^ These chemical reactions, known for their excellent biocompatibility, can proceed under physiological conditions without toxic by-products or catalysts. Moreover, the bio-orthogonal reactive pairs are highly selective and specific, not cross-reacting with commonly functional groups in the body (e.g., amines and sulfhydryls), thus avoiding unintended reactions that could interfere with normal biological processes.^279^ Therefore, incorporating bio-orthogonal chemistry in hydrogel development for cell therapy is considered a safe and promising strategy, whether for directly constructing covalently crosslinked hydrogel scaffolds or coupling bioactive components or therapeutic agents into the gel matrix.^280,281^

In contrast, dynamic crosslinked hydrogels can be formed through reversible covalent bonds—including Schiff base reactions, boronate esters, and thioester bonds—or through physical interactions, such as including electrostatic interactions, the hydrophobic effect, hydrogen bonding, π–π stacking, metal coordination, and host–guest interactions.^282^ Although these hydrogels may not be as mechanically stable as their covalently crosslinked counterparts, the reversible nature of their crosslinks renders them more advantageous for biological and medical applications. Specifically, dynamic crosslinking strategies impart viscoelastic properties to the hydrogels, making them mechanically similar to the native ECM and closer to the natural flexibility of the body’s tissues.^283^ Their self-healing capabilities enhance hydrogel durability, enabling their use in mechanically stressed environments like articular cartilage or skin, which often necessitate to withstand repeated stress.^284,285^ Additionally, their shear-thinning properties enable minimally invasive injection through narrow syringes while dissipating shear and compressive forces to reduce potential damage to encapsulated cells during the injection process.^286^ Furthermore, the inherent dynamics of these bonds endow these otherwise static matrices with responsiveness and adaptability. For instance, boronate esters, as typical dynamic covalent bonds, exhibit a wide range of responsiveness, including to pH changes, reactive oxygen and nitrogen species, and glucose levels, making them rather useful in disease-specific contexts.^287^

In terms of physically crosslinked hydrogels (i.e., supramolecular hydrogels), a prominent advantage of physical crosslinking lies in its high biomedical safety thanks to obviating the need for chemical crosslinkers, thus bypassing the cytotoxicity risk caused by unreacted chemical agents. In comparison to dynamic covalent bonds, physical interactions (e.g., hydrogen bonding and metal coordination) tend to exhibit relatively fast dynamics. This characteristic enables such hydrogels to exhibit faster self-healing and impressive low-temperature self-healing capabilities, further extending their application scopes.^288,289^ Nonetheless, hydrogels formed via physical crosslinking often possess inferior mechanical properties and are susceptible to rapid disassembly and erosion relative to their dynamically covalent crosslinked counterparts. Based on these, the development of IPN hydrogels emerges as a strategic approach for these challenges. By integrating IPN hydrogels, it is possible to significantly improve the mechanical performance of physically crosslinked hydrogels while concurrently maintaining their inherent dynamic remodeling capabilities.^290^ This advancement effectively addresses the limitations associated with singular crosslinking methods, offering a balanced solution that leverages the strengths of both physical and covalent crosslinking mechanisms.

##### Mechanical stiffness

Another pivotal parameter for the design of hydrogels is their stiffness, spanning a broad spectrum from the low kilopascals (kPa), akin to brain tissue (approximately 1-3 kPa^291^), to the megapascals (MPa), matching to cartilage (~0.2–6.2 MPa^292^). A straightforward method to modulate hydrogel stiffness involves adjusting the polymer concentration or the crosslinking density, or both. For example, enhancing the stiffness of GelMA hydrogels can be readily achieved by increasing the precursor concentration or extending UV exposure duration to induce a higher degree of crosslinking among polymer chains.^293^ However, this approach may concurrently affect other hydrogel properties, such as mesh size and the density of bioactive motifs, especially in hydrogels derived from natural sources. For instance, changing the divalent cation Ca^2+^ concentration—a common crosslinker in alginate-based hydrogels—can alter gels’ stiffness, yet this change can also impact various cellular processes, such as signal transduction, cellular motility, and mitochondrial energy production, notably in Ca^2+^-sensitive cell types like neurons and cardiac myocytes.^294,295^

Accordingly, research has focused on developing secondary polymer networks (e.g., hybrid, interpenetrating, or semi-interpenetrating networks) or integrating additional materials, such as micro- and nanoparticles (e.g., silica or metal NPs), or fibers to reinforce mechanical properties without compromising bio-functionality.^296–298^ For instance, Long and colleagues developed a silk-collagen hybrid hydrogel, where silk fibroin treated with ultrasound sonication induced antiparallel β-sheet structures, significantly strengthening the interaction within the collagen fiber network and thus enhancing the gel’s mechanical integrity for cartilage tissue engineering.^299^ Integration of carbon nanotubes (CNTs) into hydrogel scaffolds represents another viable strategy to improve gels’ mechanical performance, which is particularly suitable for nerve or cardiac tissue engineering owing to CNTs’ unique mechanical and electrophysiological strengths.^300,301^

The design of hydrogel stiffness is critical not only for improving the durability of the gel within the body and meeting the specific mechanical requirements of various tissues but also for directing cellular processes via cell-matrix interactions. It has been increasingly acknowledged that cells are mechanically dependent on the ECM, affecting their behavior even beyond simple biochemical signaling. Exposure to matrices of differing stiffness can result in diverse outcomes for their survival, phenotype, functionality, and lineage commitment. For instance, NSCs only survive in matrices with a stiffness range of 0.1 kPa to 100 kPa; with softer matrices (0.1–1 kPa) promoting neuronal differentiation, whereas stiffer ones (7–10 kPa) favoring glial differentiation.^302^ Similarly, the stiffening of the matrix can guide the differentiation of hMSCs from adipogenic to osteogenic lineages, reflecting the natural stiffness gradient between adipose tissue and bone.^303,304^ Additionally, matrices with greater stiffness tend to induce a pro-inflammatory phenotype in macrophages compared to their softer counterparts.^305,306^

**Fig. 5:**
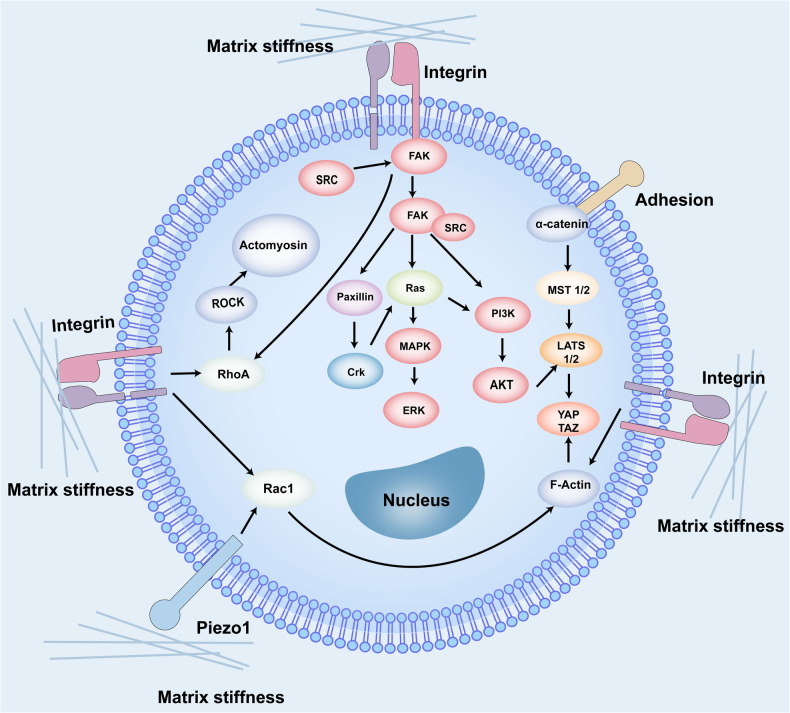
The cell mechanotransduction pathways induced by hydrogel stiffness. The matrix stiffness can be sensed by integrins, triggering signal transduction that leads to the specific activation of intracellular pathways including the FAK, Rho GTPase, and Hippo pathways. Mechanical stimuli prompt integrin subunits to activate FAK, triggering a signaling cascade involving Paxillin, SRC kinase, the PI3K/AKT pathway, and the MAPK/ERK pathway. Concurrently, external stress leads to RhoA activation, resulting in actin cytoskeleton contraction. Piezo1, an ion channel, responds to mechanical stimuli by activating Rac1 of the Rho GTPase family. Changes in stiffness also activate the Hippo pathway, centered around a kinase cascade that includes MST1/2 and LATS1/2, impacting downstream effectors such as YAP and TAZ. Information is collected from published work.^315,347,826–828^ Adobe Illustrator was used to generate this figure

Mechanistically, the stiffness of the ECM can be sensed by integrins, triggering signal transduction that leads to specific activation or relocalization of intracellular signaling molecules. This process regulates gene expression, thereby affecting cell morphology and motility. The signaling pathways triggered by changes in stiffness encompass various cellular structures and molecules, emphasizing the complexity of cell-component interactions. These pathways include the focal adhesion kinase (FAK) pathway, Rho pathway, and Hippo pathway, among others (Fig. 5).

*FAK pathway*: The FAK signaling transduction process plays a crucial role in cells’ detection of external mechanical stiffness. Upon exposure to mechanical stimuli, such as droplet or fluid shear stress, integrin subunits like α5 and αV become activated.^307^ This activation leads to their aggregation on the cell membrane, forming focal adhesions. The subsequent activation of FAK initiates a cascade of signaling events involving molecules such as Paxillin, SRC kinase, the PI3K/AKT signaling pathway, and the MAPK/ERK signaling pathway.^293,308,309^ These pathways collectively orchestrate the reorganization and contraction of the intracellular actomyosin cytoskeleton, underscoring FAK’s pivotal role in both physiological and pathological cellular processes. Paxillin and SRC kinase are primarily involved in dynamic changes and migration of the cellular cytoskeleton, whereas the PI3K/AKT and MAPK/ERK pathways have broader biological effects, including cell proliferation, survival, and differentiation. Research indicates a correlation between changes in matrix stiffness and the activation levels of FAK signaling molecules. For example, Liu’s study examined the impact of gelatin hydrogel stiffness ranging from 2.34 ± 1.48 kPa to 24.09 ± 14.03 kPa on tendon-derived stem cells (TDSCs) proliferation and differentiation. The study found that increased matrix stiffness enhanced the phosphorylation of FAK and ERK1/2, leading to increased proliferation of TDSCs and formation of more stress fibers.^310^

*Rho GTPase signaling pathway*: Members of the Rho GTPase family, including RhoA, Rac1, and Cdc42, play a pivotal role in modulating cell morphology and movement by controlling cytoskeletal dynamics.^311^ This pathway is instrumental in determining cell polarity and directionality of movement by influencing the dynamics at the cell’s leading and trailing edges. The concentration of Rac1 and Cdc42 is highest at the leading edge of migrating cells, facilitating the formation of a flat front and membrane extensions, thus directing cell movement.^312^ Activation of RhoA results in actin cytoskeleton contraction,^313^ forming stress fibers that are crucial for the retraction of the cell’s rear during movement and morphological alterations.^314^ Furthermore, these variations in cytoskeletal dynamics and cell membrane morphology are key in regulating the cell’s phagocytic capacity^305^ and endocytosis. In a study by Gan,^315^ the impact of hydrogel stiffness on the phagocytic activity of embedded macrophages was examined. This research revealed that cells in hydrogels with the highest elastic modulus (106 kPa) demonstrated elevated levels of Piezo1 and phosphorylated Rac1 (p-Rac1). Piezo1, an ion channel protein, upon activation, can trigger Rac1 of the Rho GTPase family, thereby orchestrating the reorganization of the actin cytoskeleton and initiating phagocytosis. Macrophages from rigid hydrogels exhibited significantly higher F-actin polymerization rates at bacterial adhesion sites compared to those from less rigid environments.

*Hippo signaling pathway*: At the core of the mammalian Hippo pathway is a kinase cascade comprising mammalian Ste20-like kinase1/2 (MST1/2) and large tumor suppressor1/2 (LATS1/2), along with downstream effectors, notably the transcriptional co-activators Yes-associated protein (YAP) and transcriptional coactivator with PDZ-binding motif (TAZ).^316^ MST1 and MST2, as upstream kinases, initiate the cascade by phosphorylating and activating LATS1/2 kinases. In turn, activated LATS1/2 phosphorylates YAP and TAZ, inhibiting their activity. This core kinase cascade orchestrates transcriptional programs governing cell proliferation, survival, motility, stemness, and differentiation.^317,318^ YAP/TAZ, key intracellular regulatory factors, are modulated by mechanical signals, including the stiffness of the external environment. Changes in substrate stiffness lead to YAP/TAZ relocalization, thereby affecting cellular behavior.^319^ Research involving mammary epithelial cells cultured on acrylamide hydrogels, with elastic moduli spanning 0.7–40 kPa (mirroring natural tissue elasticity), revealed distinct responses to substrate stiffness. On softer substrates (0.7–1 kPa), YAP/TAZ activity is inhibited, and localization is predominantly cytoplasmic, indicating a quiescent state under lower stiffness conditions. Conversely, on stiffer hydrogels (15–40 kPa), YAP/TAZ translocates to the nucleus, stimulating the transcription of genes associated with proliferation, suggesting enhanced activity and promotion of cell proliferation under higher stiffness conditions.^320,321^ However, a study on Schwann cells cultured on polyacrylamide hydrogels, with elastic moduli ranging from 0.5 kPa to 40 kPa and on polydimethylsiloxane at 4 MPa, revealed a contrasting pattern. In these cells, YAP/TAZ remained cytoplasmic across a broad range of stiffness, localizing to the nucleus only on extremely stiff (4 GPa) glass surfaces.^319^ This highlights that YAP/TAZ’s response to substrate stiffness is cell-type-dependent.

It is noteworthy that integrins are pivotal sensors that allow cells to perceive extracellular mechanical stiffness, and they encompass a broader spectrum of receptors and signaling molecules. Research has demonstrated integrins’ key role in the coordinated regulation of cellular responses to their external milieu by influencing various signaling pathways. Particularly, one study highlighted an increase in osteoclastogenesis correlated with higher hydrogel stiffness.^322^ This research found that on hydrogels with high stiffness (55–68 kPa), integrin β3 expression was significantly higher than on hydrogels with low (2–10 kPa) or medium stiffness (17–45 kPa). Further analysis revealed the intricate relationship between integrin signaling, stimulated by substrate stiffness, and other signaling mechanisms. It was shown that a stiffer matrix promotes integrin β3 activation via accumulated ECM fibronectin, which in turn diminishes RhoA/ROCK2 signaling and prevents the nuclear localization of YAP.^322^ Additionally, the study discovered that RhoA acts upstream of NF-κB signal transduction, influencing the NF-κB pathway in a bidirectional manner.^322^ The interaction between integrin-mediated mechanotransduction, particularly through the RhoA-ROCK2-YAP pathway, and the biochemical signaling via NF-κB, collectively fosters angiogenesis and bone repair in osteoclast precursors. These insights underscore that responses to substrate stiffness involve interconnected signaling pathways, where mechanical cues via integrins activate a complex network of signaling cascades, adding to the system’s complexity and diversity. In addition to integrin-mediated signaling, cells can detect extracellular stiffness through other membrane proteins or receptors, including those associated with vesicles, which play a role in mechanotransduction.^323,324^ Moreover, the cellular cytoskeleton can perceive and adapt to the extracellular structure, resulting in alterations in the network of actin filaments, microtubules, and intermediate filaments, thus facilitating the cell cytoskeleton’s adaptation to the matrix’s varied structural and mechanical properties.^325,326^

##### Porosity and mesh size

Porosity and mesh size are also critical parameters significantly influencing the mechanical properties of hydrogels, as well as numerous cellular processes. As mentioned before, adjustments in the concentration of precursors or the density of crosslinking alter the spacing between polymer networks, leading to variations in mesh sizes typically ranging from 5 to 500 nm.^327^ This parameter is pivotal for cell survival within hydrogels, as it governs the diffusion coefficients of solutes within the hydrogel, thereby controlling the bidirectional exchange of bioactive factors, nutrients, gases, and metabolic wastes between the inside and outside of the scaffold.^328^ Moreover, hydrogels can be engineered to develop macroscopic pores, generally ranging in size from 10 to 500 μm,^329^ which can be achieved through methods like cryogenic treatment, solvent casting/particle leaching, gas foaming, sphere templating, and electrospinning.^330–332^

Incorporating porosity and mesh size into the design of hydrogels for cell therapy is important, as these characteristics not only dictate the permeability to cells and bioactive factors but also can affect cell proliferation, migration, and intercellular interactions within the matrix. For example, research indicates that cells in 3D matrices with smaller pores and lower porosity exhibited enhanced metabolic activity and proliferation, while those in matrices with larger pores and higher porosity showed improved aggregation and differentiation.^333^ Matrices with smaller pores can provide greater mechanical support and enhance cellular responses to mechanical stimuli, thus facilitating stable cell-matrix interactions, extending cell residence time,^27^ and promoting cell proliferation.^334^ However, smaller pore sizes may hinder the penetration of large cell aggregates and restrict cell migration, crucial for cell–cell interactions, thus potentially impairing cell differentiation.^335,336^ Moreover, excessively small pores can mechanically confine cells, obstructing cell interactions, morphological changes, proliferation, differentiation, and nutrient diffusion. On the other hand, larger pores can improve cell infiltration and migration, as well as neurovascular formation,^337^ but may also reduce cell adhesion due to a relatively lower surface area.^338^

In fact, different cell types exhibit preferences for specific material pore sizes.^339^ For instance, research showed that BMSCs, chondrocytes, and tendon stem cells preferentially exhibited optimal cell viability on scaffolds with pore sizes of 200 μm, 100 μm to 200 μm, and 300 μm, respectively.^339^ Generally, scaffolds with pore sizes ranging from 300 μm to 400 μm are associated with promoting osteogenesis, including improved osteogenic differentiation, matrix mineralization, and vasculature formation.^340–342^ Scaffolds with pore sizes between 100 μm and 200 μm are more conducive to chondrogenic differentiation and cartilage-like matrix deposition.^339,342^ The minimum porosity required for blood vessel regeneration is 30–40 μm,^343,344^ while scaffolds supporting neuron growth may have pore sizes of only a few hundred nanometers.^345^

It is also pertinent to note that changes in pore size may impact the hydrogel’s overall mechanical properties (especially stiffness, and vice versa in some cases). Increased porosity may lead to reduced stiffness due to introducing more void spaces,^335,346^ whereas smaller pores can result in a denser material that is more resistant to deformation but may mechanically confine cells. Therefore, developing systems that allow for more independent modulation of these properties, minimizing their interdependence, could provide clearer insights into their respective effects on cellular behavior. Alternatively, exploiting the synergies between these properties may achieve more effective cell regulation and therapeutic outcomes.

##### Dimensionality

The spatial dimension of hydrogels is another factor influencing cell-matrix interactions. 2D hydrogels can provide a flat surface that facilitates cell attachment and the formation of a monolayer,^347^ making it well-suited for large-scale cell culture, production, and in vitro cell therapy research. However, these 2D systems are usually inability to recapitulate the complexity of the natural ECM or to fully encompass the signals present within a 3D system.^348^ In contrast, the 3D environments can offer a more physiologically relevant context, crucial for understanding cellular responses to mechanical cues, thereby enabling a more accurate interpretation of biological data and its translation from in vitro to in vivo settings. For instance, research demonstrates significant discrepancies in cellular responses between 2D and 3D hydrogel cultures, as observed in valvular interstitial cells (VICs). In 2D cultures, VICs showed upregulation in genes associated with cell structure, motion, developmental processes, proliferation, and differentiation, while those in 3D hydrogels only exhibited upregulated genes primarily related to transport, presenting a more natural state and similarity to freshly isolated VICs.^349^ These discrepancies may be attributed to the unnatural polarity influence of VICs in the 2D environment, emphasizing the need for 3D models to fully capture cellular dynamics in a manner reflective of physiological conditions.^350^

The study of cellular responses within 3D hydrogels, however, presents considerable challenges due to the inherent non-uniformity of gel properties and cell population heterogeneity, often leading to variable cell responses.^248^ The introduction of degradable gel environments further complicates this analysis since real-time monitoring of gel property changes poses difficulties.^350^ Additionally, current research tools, particularly mechanical stretching devices, often fall short in accurately simulating the diverse mechanical stimuli cells encounter in vivo due to limitations in controlling strain distribution within hydrogels.^351^ This limitation restricts the ability to comprehensively study the influence of multiple mechanical parameters on cell behavior.^351^ Therefore, there is a need for the design of programmable mechanical stretching devices for 3D cell research, and microfluidic devices may be a method to achieve this goal. Microfluidics can be used to create microreactors or cell culture devices that allow for the mechanical manipulation of 3D cell constructs to observe the formation of 3D gels and cell mechanics. Furthermore, with advancements in active tracking technology and improved measurement resolution, micro-rheology may be utilized in the future to observe local gel degradation processes.^248,352^

##### Degradability

Incorporation degradability into hydrogels is rather necessary for cell therapy applications, since non-degradable matrices will confine cell growth, restrict space for tissue regeneration, and potentially incite chronic inflammation.^353^ One of the commonly used biodegradation strategies is hydrolysis, which refers to utilizing water molecules to disassemble the hydrogel’s polymer network. Naturally derived hydrogels inherently feature hydrolytically labile bonds—such as amide in proteins, glycosidic in carbohydrates, and phosphodiester in nucleic acids—while synthetic hydrogels can be engineered with hydrolytically sensitive linkages (e.g., ester, hydrazine, acetal bonds) to facilitate hydrolytic degradation.^354^ This mode of degradation is advantageous due to its mild conditions, absence of external triggers, and biocompatible by-products, rendering it widely applicable for in vivo cell therapy. Enzymatic degradation presents another biodegradation approach, exploiting endogenous enzymes to remodel the hydrogel matrix. Likewise, this method is intrinsic to natural-derived hydrogels (e.g., collagen, gelatin, hyaluronic acid) and can be incorporated into synthetic variants by embedding specific peptide sequences responsive to cell-secreted enzymes (e.g., matrix metalloproteinases (MMP), plasmin, elastase).^355,356^ Such enzymatic activity allows hydrogels to undergo localized remodeling by residing cells, offering a promising approach for tissue engineering applications. Despite these advantages of hydrolytic and enzymatic degradation, their unpredictable kinetics and limited control over spatiotemporal degradation remain a challenge. Thus, efforts are increasingly focused on designing hydrogels that are responsive to external stimuli (e.g., photodegradation) or specific disease microenvironments (e.g., oxidative degradation) to achieve precise degradation control and enable smart therapeutic delivery (as discussed in “Responsive design of hydrogels”).^357^

Beyond degradation mechanism, the degradation rate is another critical consideration in optimizing cell therapy outcomes. A relatively slow and controlled degradation rate is generally preferable, whether to provide cells sufficient time for anchorage, proliferation, and new ECM synthesis in tissue engineering settings or to ensure sustained delivery of therapeutic cells and agents. The degradation rate of hydrogels can be affected by many factors, such as material composition, crosslinking degree, pore structure, surface area, as well as surrounding conditions (e.g., pH and temperature),^358^ in which the commonly used strategies are by altering the monomer/crosslinker concentration, crosslinking density, and the ratio of cleavable bonds in the network backbone.^359^ The gel material’s permeability to water, enzymes, acid radicals, or other soluble triggers can also impact the degradation rate, which can be adjustable by modifying the proportion of hydrophilic and hydrophobic polymers in the gel network.^360^

Additionally, the nature of the hydrogel’s degradation by-products warrants attention, necessitating non-toxic or, preferably, bioactive outputs. For instance, research on a PEG-based hydrogel demonstrated that its acidic degradation by-products could support primary macrophage survival and M1-like activation, potentially through acidic degradation products-induced immune stimulation and increased lysosomal activity.^361^ This beneficial effect illustrates the potential to harness degradation by-products to enhance cell therapy outcomes, demonstrating that these by-products can have a role beyond mere waste.

##### Integration with other materials

Another commonly used design for hydrogel-based therapies is integrating other types of materials or structures into the hydrogel matrix to improve their bioactive functionalities, improve mechanical strength, provide morphology cues, and/or introduce stimuli responsiveness. For instance, the integration of ceramics and nanomaterials (e.g., glass nanocrystals, nanoclays),^362^ enables hydrogels to support multifaceted cell delivery and therapeutic strategies. Wang et al. ^363^ developed a composite by blending collagen hydrogel with BCP ceramics and using it with rBMMSCs/chondrocytes to repair rabbit condyle cartilage defects. This combination shows promise in cell-mediated condyle cartilage regeneration, offering a novel treatment for bone-cartilage defects. Additionally, glass nanocrystals (BGnW)^364^ are emerging as potential tools for bone tissue regeneration. Incorporating bioactive BGnW into hydrogels not only improves mechanical properties but also enhances biomineralization and stimulates osteogenic gene expression, aiding in bone tissue regeneration. In neuroscience and biomedical science, materials with high conductivity, stretchability, and cell adhesiveness are essential. Integrating conductive polymers into hydrogels confers electrical conductivity and mechanical flexibility, making them suitable for tissue-mimicking neural stimulation electrodes.^365,366^ Nanoclay hydrogels, like those developed by Tondera,^365^ demonstrate effective conductivity and biocompatibility, supporting cell adhesion, proliferation, and neuronal differentiation, showing potential in therapeutic neural prosthetic systems for recovering lost neural functions due to injury or disease. Additionally, the morphological cues provided by the matrix play a pivotal role in modulating cellular behaviors, primarily through the alteration of cell shape and polarity. To this end, fiber structures are frequently embedded within hydrogels to emulate the natural tissue architecture, thereby offering spatial guidance for cell orientation and growth. For example, Zhu and colleagues introduced oriented porous nanofibers into a dual-network hydrogel system laden with BMSCs. This design mimicked the hierarchical organization of the native intervertebral disc (IVD), effectively promoting new ECM formation and IVD repair in vivo.^367^

In conclusion, the design principles for hydrogels are centered around augmenting their functionality and versatility in controlling cellular environments, thus satisfying the stringent requirements of biomedical applications and expanding their application spectrum. The selection of design parameters should be tailored to the specific needs and goals of intended applications. Crucially, when engineering hydrogels for cell therapy purposes, it is imperative to consider the biocompatibility of the constituents, any modifications made, the conditions under which gelation occurs, as well as the characteristics of any additional materials integrated into the gel. Furthermore, understanding the impact of each physical and chemical property on cellular activities and functions is essential to optimize cell performance within these matrices. This holistic approach ensures that the designed hydrogels are not only suitable for their intended applications but also contribute to the advancement of cell therapy technologies.

### Hydrogel-mediated applications on cell therapy

#### Applications in tissue engineering and repair

Recently, hydrogel-mediated stem cell delivery has emerged as a vital method in wound healing, promoting epithelialization, angiogenesis, and regulating inflammation, offering a new strategy for tissue injury treatment.^368–370^ Stem cells, due to their multi-directional differentiation and self-replication, are effective for tissue repair. However, direct injection can be compromised by inflammatory responses in the microenvironment, risking stem cell viability and repair success. Hydrogels, capable of encapsulating stem cells and preserving their viability, have been developed in various formulations, and used with different stem cells to study their effects on tissue repair, including heart, kidneys, cartilage, and bone.

While PSCs offer extensive differentiation potential, they also add complexity and uncertainty to therapy. In cases requiring specific cell types for distinct functions, using differentiated mature cells could simplify therapy, reduce uncertainty, and improve treatment control. Mature cells, already specialized, may offer a more direct approach for tissue replacement or specific functions, like myocardial cells for heart repair. In practice, medical professionals must consider these aspects to determine the best approach based on the treatment needs and patient conditions. Treatment strategies might involve using various cell types or a combination of mature and stem cells for enhanced therapeutic effects.

##### Applications in skin repair

In skin repair applications, immediate treatment of extensive skin defects is critical to prevent complications like infections and excessive inflammation, particularly in severe burn cases. Standard hydrogel dressings have limited antimicrobial properties, and improving the survival and functionality of transplanted cells in complex wound environments is challenging.^371^ Recent advancements in biomaterials have advanced hydrogel applications. The design of ideal hydrogels (with high oxygen permeability,^372^ antimicrobial properties,^127^ cell adhesion,^373^ etc.) shows promise in maintaining transplanted cell functionality and as effective wound dressings. For example, bacterial cellulose, a natural polymer with high water absorption, purity, and porosity, shows potential in wound healing when combined with cell delivery.^127,374^ Amphiphilic materials, like sulfobetaine derivatives, with their hydrophilic and anti-protein non-specific adhesion properties, help prevent wound infections and resist foreign body reactions.^375^ A sulfobetaine-derived dextran (DSC) hydrogel, developed by Yu,^371^ demonstrated excellent contamination resistance, including against bacteria and non-specific protein adhesion. Injecting DSC with adipose-derived stem cells (ADSCs) into burn mice reduced bacterial adhesion, immune recognition, and inflammation, providing a conducive environment for ADSCs proliferation.

**Fig. 6:**
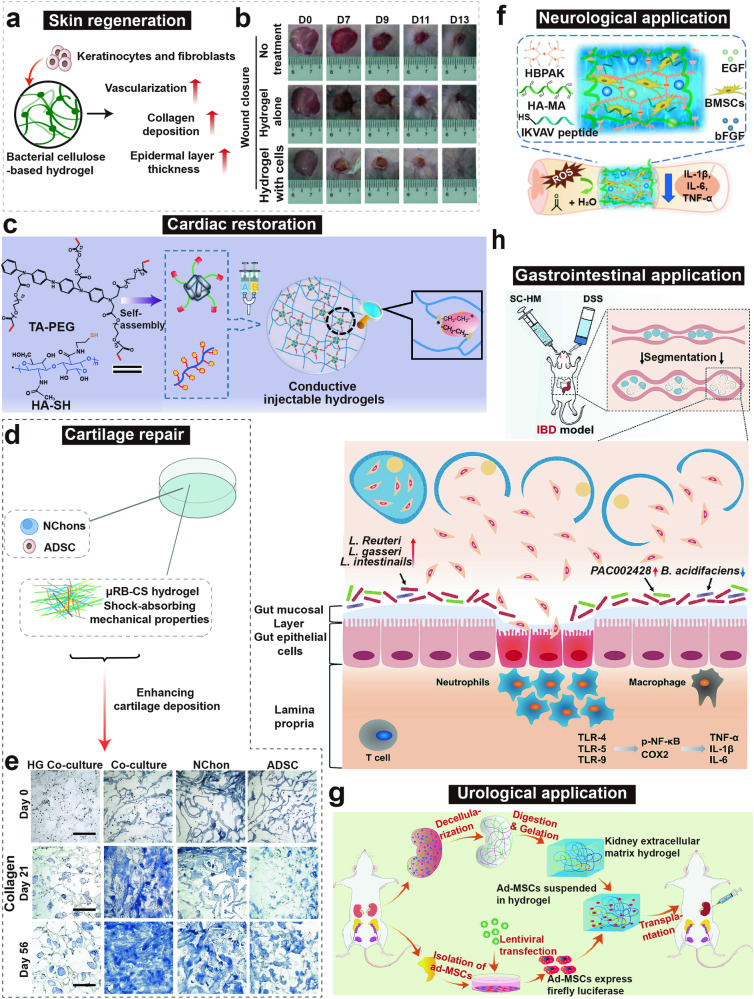
**a** Properties of bacterial cellulose-based hydrogel include high purity, flexibility, antibacterial properties, and adequate moisture balance. It is utilized for delivering keratinocytes and fibroblasts for the treatment of skin wounds. The therapeutic effects include vascularization, collagen deposition and significant epidermal thickening. **b** Images represent the rough appearance of wound closure from Day 0 (D 0) to Day 13 (D 13) for different treatment groups. Reproduced from ref. ^373^ **c** The TA-PEG/HA-SH/ADSCs/Gene hydrogel system was injected into the infarcted myocardium of Sprague-Dawley rats for the treatment of MI. (TA-PEG Tetraaniline-polyethylene glycol diacrylate; HA-SH thiolated hyaluronic acid). Reproduced with permission from ref. ^380^ Copyright 2018, Elsevier**. d** Composite scaffolds composed of macroporous gelatin microribbon/chondroitin sulfate (μRB-CS) combined with neonatal chondrocytes (NChons) and ADSCs were employed to accelerate cartilage regeneration. The hydrogel scaffold provided excellent mechanical properties. **e** The image displays masson’s trichrome staining for collagen in regenerated cartilage from different groups. Reproduced with permission from ref. ^396^ Copyright 2020, Elsevier**. f** Schematic representation of BMSCs-encapsulated ROS-scavenging hydrogel for spinal cord injury therapy. Reproduced from ref. ^182^ **g** ECM hydrogel enhances ad-MSCs for renal ischemia-reperfusion injury. Reproduced with permission from ref. ^398^ Copyright 2020, Elsevier. **h** Stem cell-loaded hydrogel microcapsules with a thin oil layer (SC-HM) encapsulate and release stem cells through segmentation and release during the peristaltic and transient phases in the intestine. Reproduced with permission from ref. ^400^ Copyright 2022, Elsevier. Adobe Illustrator was used to generate this figure

Injectable hydrogels are particularly beneficial for deep or chronic wounds with poor blood supply, such as diabetic wounds or full-thickness skin injuries. Traditional dressings or transplanted cells often fail to reach deep wound layers, complicating long-term and refractory skin wound healing. Injectable hydrogels can directly access these layers, creating a favorable microenvironment to protect delivered cells, thus preserving their vitality and functionality in situ. Zeng’s work demonstrated the efficacy of a preformed gel carrying cells for skin wound healing, highlighting injectable hydrogels’ potential as a minimally invasive option for refractory wounds.^376^ Besides stem cells, other tissue cells combined with hydrogels, like Trem2 macrophages, show clinical potential in wound healing. Transplanted Trem2 macrophages modulate inflammatory responses, reducing chronic inflammation and fibrosis at the wound site. Additionally, the collaborative action of keratinocytes and fibroblasts accelerates re-epithelialization (Fig. 6a, b).^373^

##### Applications in cardiovascular tissue repair

In myocardial infarction (MI), the adult heart’s limited regenerative ability is underscored by the expansion of necrotic tissue that catalyzes myocardial remodeling. Concurrently, fibrotic scars disrupt electrical communication, and an insufficient blood supply to the infarcted myocardium exacerbates heart damage, increasing the risk of heart failure. Stem cell transplantation is increasingly recognized as a viable therapeutic option for MI. Multiple studies^377–379^ have confirmed the positive effects of hydrogels combined with cells on cardiac function and fibrosis. In this context, hydrogels provide mechanical support to maintain structural integrity in damaged areas and regulate cell delivery. They improve cell retention and survival, ensuring effective localization and functioning of therapeutic cells. A major challenge is the loss of mechanical and electrical signal transmission due to myocardial fibrosis scar formation, as hydrogels lack electromechanical coupling with host myocardial tissue, and monitoring implantation is difficult, potentially impacting treatment outcomes. The introduction of conductive biomaterials, such as tetraaniline (TA), graphene oxide, carbon nanotubes, and metal particles, can mitigate these issues. For instance, conductive organic compounds like TA,^380^ and polypyrrole,^381^ when integrated with hydrogels, could confer electrical conductivity. Studies^382–384^ showed that incorporating conductive materials into hydrogels enhanced mechanical and electrical signal transmission in the myocardium. TA is notable for its superior electrical conductivity and biocompatibility.^385^ In a pivotal study, Wang synthesized a water-soluble conductive cross-linker by modifying PEG with TA, creating a conductive injectable hydrogel (Fig. 6c).^380^ At a concentration of 7.5% TA-PEG, the hydrogel exhibited electrical conductivity (*G* = 2.32 × 10^−4^ S/cm), aligning with the conductivity range of natural myocardial tissue (from 5 × 10^−5^ to 1.6 × 10^−3^ S/cm). This hydrogel, combined with ADSCs, was administered into the infarcted myocardium to offset cell loss post-MI and improve myocardial electrical connectivity. A resultant increase in the expression of gap junction protein Cx43 in myocardial cells was observed, indicating enhanced electrical signaling among adjacent cardiac cells and contributing to the restoration of conductivity in areas affected by the infarction.

##### Applications to bone/cartilage tissue repair

Stem cell scaffold methodologies hold significant potential for bone tissue engineering. Hydrogels, derived from biopolymers, have been widely used as effective cell carriers in regenerating various bone types (e.g., alveolar, skull),^386^ and cartilage. It is essential to note that hydrogels with larger pore sizes are generally preferred to promote osteoblast proliferation and matrix deposition. Studies^335^ have identified an optimal pore size range for osteoblastic activity, specifically 350–400 μm, with a porosity of 30–90%. However, conventional injectable polymer-carriers and hydrogels often lack the necessary durability for weight-bearing orthopedic applications. This has led to the development of new injectable polymers with enhanced load-bearing capabilities. The mechanical properties and stability of hydrogels can be significantly improved by incorporating materials like calcium phosphates, silicates,^226,387,388^ polyvinyl alcohol, collagen,^389,390^ cellulose, nanofibrillated cellulose,^391^ and various crosslinkers.^392^ Selecting scaffold materials and structures that provide adequate rigidity, strength, and elastic modulus is crucial for fostering new bone formation and integrating with natural bone tissue. Therefore, creating a scaffold that mirrors the mechanical properties of cancellous bone is a key objective in bone tissue engineering.

The limited self-healing capacity of cartilage, due to its avascular nature and the inability of chondrocytes to migrate to injury sites, necessitates alternative strategies for repairing cartilage lesions. Hydrogels with structural and biochemical biomimetic characteristics are considered a promising solution for effective cartilage regeneration. These hydrogels not only fill cartilage defects but can also repair both cartilage and the underlying subchondral bone,^393^ which supports mechanical strength and promotes cell migration and new tissue growth.^394^ Moreover, since cartilage injuries often trigger inflammatory reactions that can hinder repair, designing hydrogels with anti-inflammatory properties is beneficial. For instance, a novel poly-anionic hydrogel enriched with carboxylate/sulfonate groups and closely cross-linked with Fe^3+^ has shown effectiveness in suppressing hydroxyl radicals and nitric oxide in macrophages, protecting chondrocytes/fibroblasts from severe inflammation.^395^ Controlled release techniques and carriers that deliver bioactive substances, such as drugs and growth factors, to the injury site can also enhance cartilage regeneration by moderating inflammation. Additionally, the selection of cell sources for transplantation is critical. Due to challenges in harvesting cartilage cells and the subpar mechanical performance of differentiated cartilage from stem cells, a mixed population of chondrocytes (NChons) and stem cells (ADSC) is being explored as a potential cell source for cartilage regeneration (Fig. 6d, e).^396^ Studies have shown that optimizing the ratio of mixed cells and cultivation conditions can more effectively facilitate the formation of articular cartilage.

##### Applications in other tissue repairs

In the field of tissue engineering, hydrogel-based cell therapies have made considerable advances in the repair of various organs, spanning the nervous system, kidneys, gastrointestinal tract, respiratory epithelium, ocular tissues, and liver. These advancements address critical needs in organ tissue repair, demonstrating significant therapeutic potential.

For example, in neurotissue engineering, traumatic spinal cord injuries result in an overproduction of ROS at the lesion sites, leading to the necrosis and apoptosis of transplanted BMSCs. Hydrogels containing ROS scavengers can mitigate this excess ROS, reducing oxidative stress and inflammation to improve BMSCs survival. This approach significantly enhanced neurogenesis, and motor recovery, and reduced scar formation (Fig. 6f).^182^ In the context of acute kidney injury caused by ischemia-reperfusion, the local injection of MSCs into the renal parenchyma has been explored. Nonetheless, this method is hampered by post-transplantation cell loss and acute cell death due to the hostile microenvironment.^397,398^ Combining ad-MSCs with ECM hydrogels has proven to effectively enhance ad-MSCs retention (Fig. 6g), increase cell proliferation and neovascularization, and reduce renal tubular damage, thereby improving renal function after injury.^398^ In gastrointestinal engineering, the low delivery rate of transplanted cells to the gastrointestinal tract impedes the clinical potential of stem cell therapy.^399^ Utilizing monodisperse hydrogel microcapsules as oral delivery carriers enabled MSC transport directly to the gastrointestinal tract, protecting them from the acidic external environment and improving their survival and functionality (Fig. 6h).^400^

Additionally, in respiratory epithelium repair, cell-loaded collagen hydrogels have been employed to evenly distribute exogenous MSCs across de-epithelialized rat tracheas, treating airway epithelial damage. This method enhanced cell adhesion and survival, showcasing the potential for treating diseases like primary ciliary dyskinesia and chronic obstructive pulmonary disease.^401^ Ocular tissue engineering has seen the use of hydrogels for sustained delivery of therapeutic agents, such as in a study where HEK293 cells, overexpressing the neuroprotective agent GDNF, were encapsulated in collagen and alginate-based hydrogels. This facilitated sustained delivery of GDNF, promoting photoreceptor survival in a model of genetic retinal degeneration.^402^ In liver tissue engineering, co-transplantation of allogeneic liver cells and BMMSCs within biopolymer and collagen-based hydrogels has shown promise in a rat model of liver failure, indicating structural liver repair, prevention of necrosis, and reduction in liver fibrosis transformation by the 30th-day post-transplantation.^403^ Finally, in the treatment of inflammatory bowel disease, hydrogels have been instrumental in enhancing MSC survival in the gastrointestinal tract, significantly reducing pro-inflammatory factor secretion and macrophage infiltration, thereby improving the microbial balance and reducing disease severity.^400^

Despite these advances, tissue engineering research still faces challenges in cell culture, scaffold design, tissue integration, functional maintenance, immunological rejection, and long-term post-transplantation stability. The field is predominantly in its foundational stages, with the future focused on advancing technologies in biomaterials, cell engineering, and bioprinting to simulate and rebuild complex physiological structures for enhanced clinical therapy applications more effectively.

#### Applications in tumor immunotherapy

**Fig. 7:**
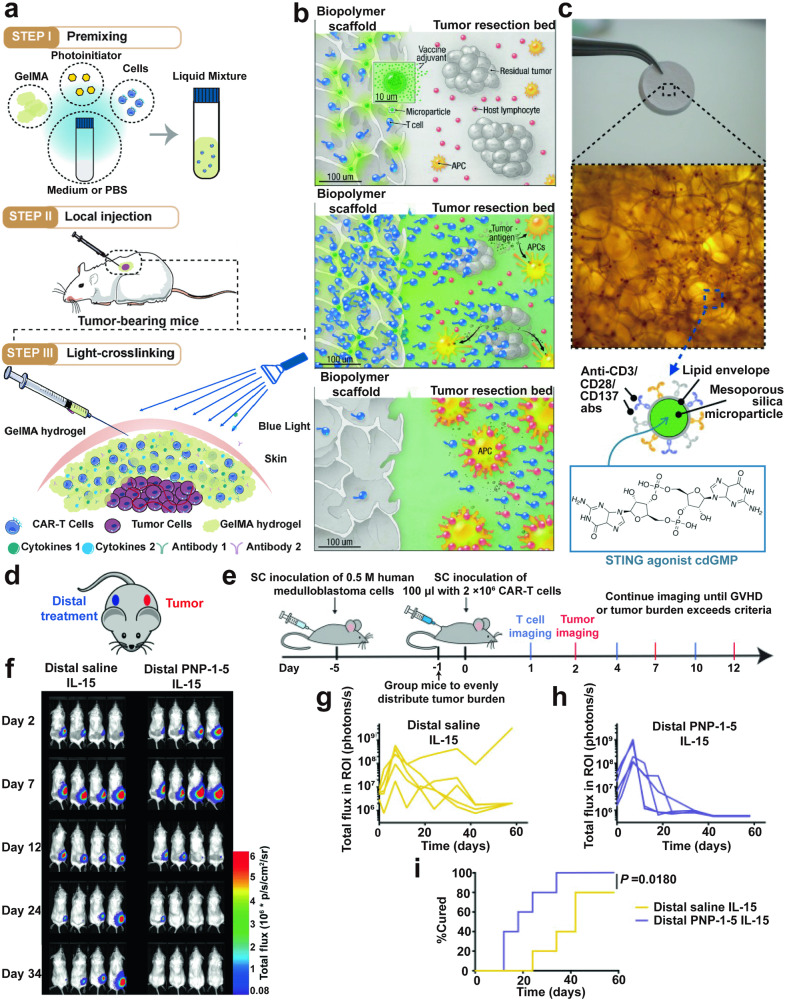
**a** Schematic diagram of the installation process of the injectable delivery system. The first step is to mix the cells, cytokines and hydrogel solutions. The second step is to inject the mixed solution into the tumor site. The final step is photo-curing by blue light irradiation. The hydrogels loaded with CAR-T cells solidify locally at the tumor site and continuously release T cells to kill tumor cells. Reproduced with permission from ref. ^405^ Copyright 2022, Elsevier. **b**, **c** Design of a biomaterial carrier that codelivers CAR-expressing T cells and vaccine adjuvant to simultaneously clear heterogeneous cancer cells and establish systemic antitumor immunity. Macro- and microscopic views of a porous alginate matrix functionalized with microparticles that have the STING agonist cdGMP entrapped in the polymer core and stimulatory anti-CD3/CD28/CD137 antibodies tethered to their phospholipid membrane. Schematic shows the chemical structure of cdGMP. Reproduced with permission from ref. ^411^ Copyright 2017, The American Society for Clinical Investigation. **d**, **e** Experimental timeline and schematic illustration of treatment placement for murine distal subcutaneous medulloblastoma using PNP hydrogel. Representative in vivo bioluminescence imaging of tumors (**f**), quantitative analysis of imaging data for the two experimental groups (**g**, **h**), and inter-group cure percentage (**i**). **d**–**i** reproduced from ref. ^59^ Adobe Illustrator was used to generate this figure

Immunotherapy has emerged as a significant approach in tumor treatment, with ACT, notably CAR-T and TCR-T cells, demonstrating efficacy in treating diverse tumors. The emphasis, particularly on CAR-T and TCR-T cells, aligns with their therapeutic potential. However, the effectiveness of CAR-T cells in treating solid tumors faces limitations due to various factors. To overcome this, there is a proposal to employ biomaterials, particularly hydrogels, to improve treatment. Studies indicate that biomaterial scaffolds, like hydrogels, significantly activate CAR-T cells at the tumor site and enable localized cytokine delivery, minimizing systemic exposure and reducing toxicity (Fig. 7a).^404–406^ Furthermore, high CAR-T cell activation, which requires cytokines, often leads to systemic effects due to the expansion of cytokines at high concentrations. Research showed that modifying hydrogel structures can influence the behavior of T cells at tumor sites,^59^ indicating the possibility of more precise targeting of solid tumors by controlling T cell activity. In light of the challenges posed by the immunosuppressive TMEs, future research could involve incorporating immune stimulants and checkpoint inhibitors into hydrogel carriers for CAR-T delivery.^28^ This approach may enhance the recruitment of endogenous immune cells, amplifying the anti-tumor response. These strategies could also apply to other adoptive cell therapies (TCR-T, NK, DCs, etc.) delivered to localized tumor regions.^117,406–408^

Another significant approach in immunotherapy is therapeutic tumor vaccines, particularly those involving DCs from patients. These cells are combined with tumor antigens or immune stimulants in the lab, processed, and reintroduced into the patient to activate the immune system against cancer cells. DC vaccines are particularly advantageous when tumor-specific antigens are unknown, as DCs are efficient APCs crucial for initiating robust immune responses.^409^ However, the infiltration and functionality of tumor-associated DCs are often suboptimal within the tumor host. Augmenting the host immune response can involve reinfusing host DCs loaded with tumor antigens in vitro. Strategies include combining tumor whole cells and DCs vaccines with immunostimulants (nano-adjuvants, anti-PD-1 antibodies, etc.) through hydrogel delivery^117,410,411^ (Fig. 7b-c). Nano-adjuvants enhance antigen presentation and promote DC maturation, driving immune activity. Hydrogels not only extend the efficacy of whole-tumor cell vaccines but also provide a conducive environment for endogenous DC recruitment. Given the limited immunogenicity of tumor whole-cell vaccines and the short lifespan of DC vaccines, previous studies have faced challenges in achieving satisfactory clinical outcomes. Therefore, enhancing the capacity of cell-based vaccines to elicit strong, enduring immune responses has been a primary objective. Recent findings underscore the significant roles of immunostimulatory agents and hydrogels in boosting the efficacy of immune vaccines, supporting the development of more effective immunotherapeutic strategies.

Addressing recurrence after tumor resection, the development of responsive degradable hydrogels, tailored to specific tumor characteristics like the TMEs, is crucial for monitoring recurrent tumor cells. Cheng et al. ^113^ developed a dual pH-responsive hydrogel containing a tumor acidity-neutralizing agent and a NETs-cleaving enzyme (DNase I). This hydrogel, used alongside NK cell infusion, effectively prevented hepatocellular carcinoma (HCC) recurrence post-resection. Injected at the incision margin, the hydrogel transitions into an adhesive gel, aiding rapid hemostasis, reducing tumor acidity, limiting immunosuppressive cell infiltration, and releasing DNase I in a pH-responsive manner to degrade NETs. This study demonstrated that combining NK cells with hydrogels could effectively prevent HCC recurrence. Additionally, hydrogels combined with cells show potential for distal tumor treatment. Studies by Grosskopf^59^ using CAR-T cells and IL-15 within a polymer nanoparticle hydrogel showed rapid cure in mice with untreated tumors, highlighting the efficacy of this approach for treating occult metastases or tumors through direct injection or catheter delivery, despite a longer treatment duration compared to peritumoral injection (Fig. 7d–i).

#### Applications in inflammatory disease treatment

In addition to their established roles in tissue engineering and tumor immunotherapy, hydrogel cell scaffolds are being developed for a wide range of applications, including the treatment of inflammatory diseases such as periodontitis, vocal cord scarring, rheumatoid arthritis, and inflammatory bowel disease.

**Fig. 8:**
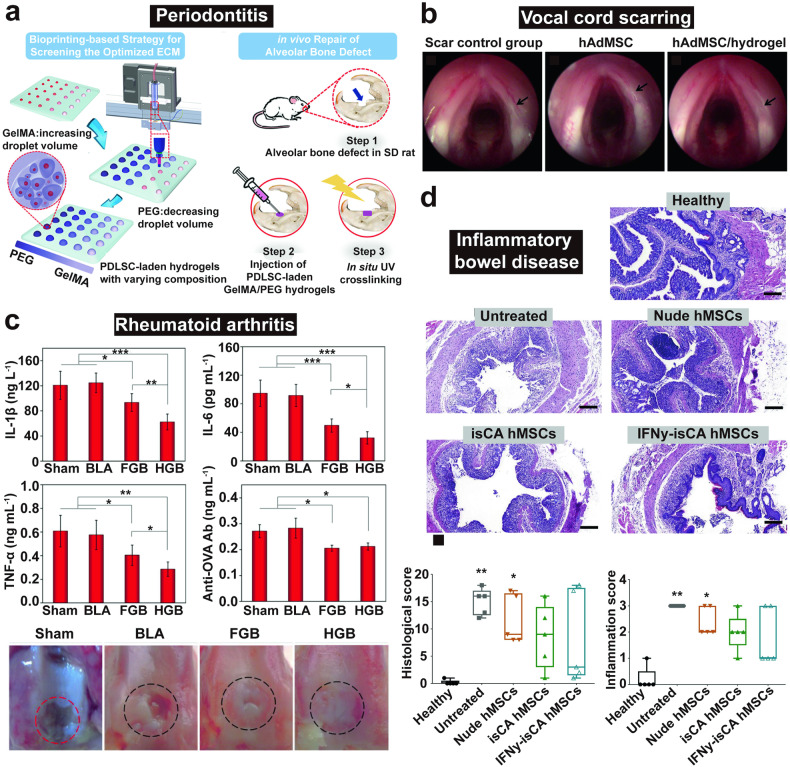
**a** Schematic representation of the strategy for repairing alveolar bone defects in vivo using a hydrogel microarray encapsulating periodontal ligament stem cells (PDLSCs) based on bioprinting. Reproduced with permission from ref. ^386^ Copyright 2017, American Chemical Society. **b** Gross observation results of vocal fold scar formation three months post-injury. Morphological improvements were observed in both the hAdMSCs and hAdMSsC/hydrogel groups, showing smaller fibrotic bands (arrows). Reproduced with permission from ref. ^416^ Copyright 2013, John Wiley and Sons. **c** Concentrations of IL-1β, IL-6, TNF-α, anti-OVA antibodies in serum were measured by ELISA, along with the macroscopic appearance of rheumatoid arthritis cartilage after 12 weeks post-transplantation for four different groups: sham, blank (BLA), fibroblast gel-BMMSCs (FGB), and hydrogel-BMMSCs (HGB). Reproduced from ref. ^418^ **d** Histological examination of the therapeutic effects of adipose tissue-derived human MSCs encapsulated in injectable hydrogel in mice with acute colitis induced by dextran sodium sulfate and histological scoring was performed. Reproduced with permission from ref. ^419^ Copyright 2022, Elsevier. Adobe Illustrator was used to generate this figure

Periodontitis is a chronic inflammatory disease affecting the periodontal tissue, leading to the deterioration of structures supporting teeth, including alveolar bone, cementum, and periodontal ligament (Fig. 8a).^386^ Periodontal therapy aims to reduce infection and restore the structural and functional integrity of these tissues.^412^ Traditional treatments for periodontal disease face limitations like poor biodistribution, low therapeutic specificity, abrupt drug release, and unintentional damage to healthy cells.^413^ A promising approach involves a polymer-based material with anti-inflammatory and regenerative properties, combined with periodontal stem cells, offering immunomodulatory and potent regenerative effects, poised to address these therapeutic challenges in oral disease.^386,414^ Vocal cord scarring, a major cause of severe voice disorders, is characterized by the replacement of the lamina propria’s ECM with disordered collagen bundles, leading to voice issues like dysphonia or aphonia.^415^ Current treatments for vocal cord scars are limited. MSCs have shown potential in enhancing wound healing and improving vibrational capacity and elasticity of the vocal folds, but they struggle to survive in damaged vocal cords^416,417^ (Fig. 8b). Rheumatoid arthritis (RA) results in persistent cartilage damage due to chronic inflammation and synovial hyperplasia, making cartilage repair challenging. Unlike damage from trauma or osteoarthritis, RA-related damage continues despite treatment. BMMSCs have been used to mitigate RA, but their efficacy in stopping cartilage destruction is limited. To improve outcomes, researchers are integrating BMMSCs with auxiliary scaffolds in cartilage tissue engineering (Fig. 8c).^418^ Inflammatory bowel disease treatment relies on immunomodulatory factors from MSCs, but conventional methods often lead to rapid loss of these cells’ immunomodulatory phenotype. A novel hydrogel system has been developed to overcome these limitations, ensuring sustained delivery of bioactive factors^419,420^ (Fig. 8d).

It has been demonstrated that scaffold-based delivery systems possess high specificity, enabling slow release to reduce cell death and target site leakage, thereby favoring the maintenance of effective cell viability. This approach becomes a method to improve the quality and effectiveness of inflammation treatment. Targeted cell therapy of this nature is an active area of ongoing research, and methods combining cells with injectable hydrogels offer a conducive microenvironment for the treatment of inflammatory diseases.

### Considerations in opting for cell therapy

During the application, the decision to use cell therapy requires consideration of multiple factors. Firstly, considering the advantages and disadvantages of cell therapy, its primary advantage is to offer highly personalized treatment plans that can eradicate certain significant diseases. For instance, CAR-T cell therapy has achieved complete remission in leukemia cases. Moreover, in treating severe skin burns or chronic wounds, stem cell applications through cell therapy facilitate the repair and regeneration of damaged tissues, directly addressing the disease source.^421^ These successful cases underscore cell therapy’s significant therapeutic potential and its capacity to reduce the frequency of treatments, offering long-term, and in some cases, lifelong benefits. However, the application of cell therapy faces numerous challenges. The complexity of the technology and associated procedures, encompassing cell collection, cultivation, modification, and reinfusion, demands precision and control to guarantee safety and efficacy.^422^ Furthermore, cell therapy often results in high costs, limiting its widespread adoption and accessibility.^423^ Safety issues also pose significant challenges for cell therapy, including the risk of potential immune reactions.^424^ For example, even the use of a patient’s own cells for treatment can trigger unpredictable immune responses.^425^ The risk of cell over-proliferation or ectopic growth cannot be overlooked either, especially when using stem cells with powerful regenerative capabilities, as uncontrolled cell proliferation may lead to new diseases such as cancer.^426–428^

Secondly, upon evaluating the severity of conditions and corresponding treatment requirements, the distinct application contexts and benefits of cell therapy become exceedingly apparent. In cases of serious tissue damage or functional loss, such as severe cardiac diseases, liver diseases, or specific degenerative disorders, cell therapy offers a targeted solution by directly providing cell replacement or repair, addressing the core problems associated with these conditions.^429,430^ For instance, in patients with severe cardiac conditions, the injection of MSCs into damaged cardiac tissue can promote the repair and regeneration of heart tissue, an outcome that traditional pharmacological treatments cannot achieve.^431^ Within the severe liver disease treatment, cell therapy techniques like hepatocyte transplantation offer a potential treatment option for end-stage liver disease patients, enhancing liver function and potentially precluding the necessity for organ transplantation.^430^ Moreover, cell therapy also demonstrates its substantial advantage when highly personalized treatments are required. In the treatment of certain genetic diseases, such as specific types of genetic retinal diseases, cell therapy can utilize the patient’s own cells for genetic correction before reinfusion, aiming to fundamentally solve genetic defects and restore or improve vision.^432^ For certain types of degenerative diseases, like Parkinson’s disease, the injection of human pluripotent stem cells into the brain can alleviate symptoms and decelerate disease progression.^433^

Lastly, from the perspectives of resource availability and ethical considerations, the application of cell therapy faces a complex decision-making environment.^424,434^ In situations where resources are abundant and high costs can be sustained, despite the high expense of cell therapy, it remains a consideration for patients with severe diseases who could see significant improvements in quality of life or for whom it represents the only viable treatment option, especially when other treatments have failed. Concurrently, cell therapy applications involving embryonic stem cell research and gene-editing technologies like CRISPR may elicit a range of ethical concerns.^435,436^ For instance, the employment of gene-editing technology harbors inherent risks and ethical quandaries concerning the modification of human genetic material, with the possibility of inadvertently affecting other genes and precipitating unexpected adverse effects.^437^

Therefore, taking into consideration the aforementioned factors, in contexts where resources are limited, costs and ethical considerations are paramount, non-cell therapies (such as targeted drugs, peptide medications, and protein therapies) become the preferred option for non-acute or mild to moderate diseases. For example, in early-stage cancer treatment, targeted drugs utilize specific molecular markers of tumors to precisely kill cancer cells, significantly reducing the impact on normal cells and thereby relatively minimizing side effects.^438^ Similarly, for the repair of minor tissue damage, such as skin damage caused by mild to moderate diabetes or minor arthritis, peptide and protein therapies, by mimicking or enhancing the body’s natural healing processes—such as promoting the wound healing action of growth factors or the effect of anti-inflammatory peptides—facilitating the repair and functional restoration of affected tissues, effectively managing these disease states without relying on more complex and costly cell therapies.^439^

Hydrogels have seen widespread use in non-cell therapies, where their functionality extends further as advanced delivery systems for drugs, proteins, or other therapeutic molecules.^440^ By finely tuning the network structure and chemical properties of hydrogels, it is possible to achieve precise control over the rate and duration of drug release, enhancing therapeutic effects while reducing side effects. The development of responsive hydrogels has realized sensitive reactions to changes within the internal environments, such as pH, temperature, or specific biomarkers, adjusting drug release behavior as needed.^441^ This controlled release behavior is especially suited for the long-term management and treatment of chronic diseases, ensuring the continuity and stability of drug delivery. Beyond serving as drug delivery vehicles, hydrogels demonstrate additional possibilities in biomedical applications. For instance, bioadhesives leveraging the adhesive properties of hydrogels provide an effective method for connecting or repairing damaged tissues in tissue engineering and surgical operations.^442^ Hydrogel-based artificial tissues promise to revolutionize tissue engineering and regenerative medicine by replacing damaged tissues.^443^ Hydrogel-based biosensors, adept at detecting and responding to biomolecular changes, hold promise for disease diagnostics and monitoring.^444^ The innovative applications of these technologies will be elaborated upon in the subsequent section.

## Hydrogels for non-cell therapy

Hydrogels are extensively utilized as delivery systems within the domain of non-cell therapy.^161,445,446^ Traditional chemically synthesized drugs aim to impact specific targets within lesion sites. However, systemic administration—such as intravenous or oral routes—where drugs exhibit transient circulation times and undesirable accumulation in filtration organs like the liver and spleen, leads to a diminished drug concentration at the lesion and potentially elicits severe adverse bodily reactions.^447^ Furthermore, bioactive agents such as proteins, genes, and peptides are readily recognized and eliminated by the body’s immune system or enzymes upon systemic administration, thereby reducing their bioavailability.^448,449^ Notably, despite the clinical efficacy of immune checkpoint antibodies (e.g., PD-1, PD-L1 antibodies), their systemic administration can also induce immune-related adverse effects, including cardiotoxicity, dermatotoxicity, gastrointestinal toxicity, hepatotoxicity, and endocrinological toxicity.^450^ Likewise, the off-target effects of CRISPR/Cas-9 gene editing, despite its significant medical advancements and Nobel recognition, restrict its clinical utility due to unintended alterations in normal cells.^451^ These challenges underscore the critical need for more precise drug delivery to lesion sites in non-cell therapy.

Hydrogels, recognized for their biocompatibility and modifiability, present a superior platform for in vivo drug delivery.^329,452^ Drugs can be integrated into hydrogels via straightforward mixing or sophisticated physicochemical modifications, effectively shielding the drug from premature degradation and immune system attacks while preserving its biological activity. Whereas initial hydrogel formulations predominantly relied on physical passive diffusion for drug release, contemporary advancements in hydrogel technology employ both physical and chemical methodologies to refine the gel-drug interaction, facilitating both sustained and on-demand drug release, thereby enabling more sophisticated control over drug release kinetics. Beyond drug delivery, hydrogels are also widely used in other areas of non-cell therapy, such as bioadhesives, artificial tissues, and biosensors.

In this section, we first provide an overview of hydrogels’ strengths as delivery vehicles and the basic design considerations. Thereafter, we systematically discuss the application of hydrogels in drug delivery (e.g., small molecules delivery, peptide drugs delivery, protein drugs delivery, and gene drugs delivery), and physical intervention therapy. Each section highlights the challenges specific drugs face in disease treatment, the benefits of hydrogel-mediated delivery, and illustrative examples of hydrogel-facilitated therapeutic outcomes in diverse pathological contexts. In other applications (e.g., bioadhesives, artificial tissues, and biosensors), we have discussed the advantages of hydrogels in these current applications and the design considerations that must be addressed. Examples of the contributions of these applications to disease diagnostics and therapy are also presented. Through these discussions, we hope to elaborate on the pivotal role of hydrogels in contemporary medicine and underscore their significant potential for future advancements.

### Advantages and design considerations of hydrogels for non-cell therapy

#### In drug delivery

The most intuitive advantage of utilizing hydrogels lies in their intrinsic ability to localize drug delivery directly to target tissues, thereby significantly reducing off-target drug exposure. This direct targeting concentrates therapeutic agents at the site of interest, which in turn reduces the requisite dosage and minimizes systemic side effects. This feature is particularly exploited in injectable hydrogels, which, through mechanisms like in-situ gelation, shear-thinning, or shape-memory characteristics, facilitate ease of administration and minimal invasiveness, making them emerge as an ideal solution for addressing clinical and pharmacological challenges, such as the regeneration of irregular defects or the treatment of deeply situated solid tumors.^284,453,454^

More strikingly, hydrogels’ highly customizable nature for controlled drug release expands their application spectrum. Effective therapy often necessitates slow, stable, and sustained drug release patterns to prevent the risks associated with burst release while ensuring prolonged therapeutic efficacy at the targeted site. Advanced hydrogel systems are adept at facilitating these controlled release dynamics, offering continuous or, when required, pulsatile release patterns, capable of establishing long-lasting drug depots at specific sites.^455–457^ This attribute is especially advantageous for the management of chronic conditions that require long-term medication administration, such as diabetes.^458,459^

Recent research efforts have been directed toward refining the structural and compositional aspects of hydrogels to achieve a precise match between drug release profiles and therapeutic or physiological needs. For one thing, adjusting the hydrogel’s mesh size relative to the drug’s hydrodynamic dimensions is one of the fundamental strategies to yield desirable release patterns.^460,461^ For example, modifications to polymer concentration or crosslinking density are commonly employed to modulate release profiles.^462^ Conceivably, increased polymer or crosslinker contents will lead to decreased mesh size, which may reduce burst release and prolong the release time, although most of the time, achieving desired outcomes requires a multifaceted approach due to the significant size disparity between the hydrogel mesh and the drug molecules. Dynamic adjustments to hydrogel properties, such as network erosion, swelling behavior, and mechanical deformation, offer additional mechanisms to modulate drug release.^463,464^ To be specific, the introduction of hydrophobic components into the hydrogel’s backbone can effectively decelerate water penetration into the bulk hydrogels, thereby diminishing the rate of hydrolysis-induced degradation and consequently prolonging the duration of drug release. Alternatively, the integration of stimuli-responsive motifs into the hydrogel crosslinkers facilitates precise, on-demand drug release, triggered by either endogenous enzymes such as matrix metalloproteinases or thrombin, or by external stimuli, including photo-radiation or ultrasound, or magnetic fields.^1,357^ This strategic manipulation of hydrogel properties allows for a tailored approach to drug delivery, optimizing therapeutic outcomes through controlled release mechanisms.

For another, incorporating drug-polymer interactions within hydrogel formulations is a strategic approach to enhance the control over drug release mechanisms, particularly important for small molecule drugs.^465^ These molecules, often too small to be affected by the hydrogel’s mesh size sterically, can be more effectively managed through either covalent conjugation or non-covalent interactions (e.g., electrostatic interactions and hydrophobic associations). Such interactions significantly enhance the affinity between the therapeutic molecules and the hydrogel matrix, mitigating premature burst release and fostering a sustained release profile.^466,467^ Furthermore, these drug-polymer interactions are instrumental in augmenting the solubility and stability of the encapsulated agents, especially crucial for drugs with poor water solubility and sensitive biomolecules like proteins or RNA. This enhancement not only improves the drug’s bioavailability but also preserves its biological activity over time.^468,469^ More importantly, hydrogels, with numerous modifiable sites that can be used for fabricating physicochemical interactions, also accommodate multiple drug delivery.^470,471^ This capability is particularly beneficial in oncological applications, where the synergistic action of combined therapeutics is necessary to achieve optimal treatment outcomes. Further, the design of hydrogels can simultaneously include different encapsulation techniques—ranging from passive encapsulation to active tethering of drugs to the hydrogel’s structure, or stimuli-responsive elements, leading to the asynchronous release of different drugs and thus paving the way for precise spatiotemporal control over drug delivery.^171,472,473^ Such meticulously designed hydrogel systems hold the promise of maximizing therapeutic efficacy, especially in complex treatment regimes.

Despite the versatility of hydrogels, it is essential to consider that increasingly complex designs can elevate the corresponding risks, such as reduced drug activity, compromised biocompatibility, as well as increased unpredictability. Additionally, the fate of hydrogels post-drug release or degradation demands attention, particularly regarding the metabolizability and excretion of their by-products. The potential for degraded base components to elicit specific immune responses in the body is an often underestimated concern. As the field progresses, emphasis on optimizing hydrogel structures and their interactions with therapeutic agents must be balanced with rigorous evaluation of the biotoxicity, absorption, and metabolism of hydrogels in vivo. These factors can be critical in determining the feasibility of hydrogels for clinical application in non-cell therapy.

#### In other non-cell therapy

The extensive applications of hydrogels in the medical field are primarily attributed to their excellent biocompatibility, which is important for their success in different areas like bioadhesives, artificial tissues, and biosensors. In bioadhesives, hydrogels demonstrate multifunctionality and high adjustability, effectively bonding tissues and facilitating the restoration of their structure and functionality. This is enabled by the integration of polymerizable groups within hydrogels, along with their exceptional adhesive properties, such as intermolecular bonding, chain entanglement, mechanical interlocking, and electrostatic interactions.^275,474^ These materials not only halt bleeding but also prevent infection and speed up the healing process via enhanced antimicrobial properties and self-healing capabilities.^475–477^ Furthermore, hydrogel designs can be customized to meet diverse adhesive requirements from epidermal to deep tissue applications, maintaining adhesion in moist environments.^478,479^ Additionally, hydrogels inspired by natural models, using the chemistry and structures found in nature, improve their attachment to wet and moving tissues, providing revolutionary solutions for high-risk applications like cardiac surgery.^480,481^ The utilization of hydrogels as bioadhesives, with their biocompatibility, self-healing, and potent adhesive properties, brings superior therapeutic potential over traditional suturing and stapling methods.

In artificial tissues, hydrogels are celebrated for their resemblance to natural tissue structure and function, exceptional biocompatibility, and tunable mechanical properties, offering innovative solutions to complex challenges in tissue engineering and regenerative medicine. They can replicate key properties of human skin, cartilage, and blood vessels, support cell growth, and promote tissue repair and regeneration, presenting effective alternatives for tissue loss caused by injury or disease.^482–484^ For instance, hydrogels can mimic the load-bearing function of human cartilage, support the adhesion and proliferation of vascular endothelial cells, and provide an appropriate scaffold for the regeneration of cartilage and blood vessels.^484,485^ By adjusting the composition and crosslinking density of hydrogels, their degradation rate, mechanical strength, and bioactivity can be precisely controlled to fulfill the requirements of various types of tissue engineering.^284,486^

Similarly, the application of hydrogels in biosensors offers numerous advantages, particularly in enhancing biosensor performance, broadening their application range, and improving biocompatibility.^487–489^ Their exceptional biocompatibility significantly diminishes the risk of immune rejection upon implantation, while their unique physicochemical properties enable hydrogels to integrate with various internal components, increasing the sensitivity of biomarker detection. Hydrogels can detect low concentrations of biomarkers and visually monitor changes in fluorescence intensity, signaling alterations within the body.^490^ The flexibility, scalability, adhesiveness, and self-healing properties of hydrogel sensors render them highly promising for health monitoring applications. They can adjust to various dynamic changes in the body and through innovative design, achieve precise health status monitoring and data transmission.^491,492^ Furthermore, the adjustable biodegradability of hydrogels presents unique advantages for in vivo sensors, allowing for continuous health condition monitoring without necessitating surgical removal. In conclusion, the applications of hydrogels in bioadhesives, artificial tissues, and biosensors underscore their immense potential as multifunctional, highly adjustable, and biocompatible materials. These applications are instrumental in promoting wound healing, advancing tissue engineering, and enhancing health monitoring, underscoring the significant impact of hydrogels in medical science.

Although hydrogels demonstrate numerous advantages in various applications, careful consideration is also required when designing them for specific uses. Biocompatibility is most important in the medical application of hydrogels to avoid triggering adverse immune responses or promoting inflammation. Furthermore, when applying hydrogels as bioadhesives, it is essential to ensure that hydrogels possess adequate adhesive strength, adaptability, and the ability to facilitate wound healing.^493^ Adhesive strength is paramount for maintaining wound closure, particularly in deep tissues or dynamic areas like joints and internal organs, which may subject the adhesive to increased fluid pressure.^494,495^ Additionally, hydrogels must exhibit adaptability and flexibility to seamlessly adhere to and accommodate the irregular surfaces of wounds and the dynamic changes of tissue remodeling.^496^ For internal applications, the prevention of tissue adhesion is a further critical aspect. In practical applications, the ideal bio-adhesive should be simple to apply, quickly solidify to reduce surgical duration and exhibit physical and chemical properties post-curing that support wound physiology.

When hydrogels are applied to artificial tissues, their mechanical properties must precisely match the characteristics of the target tissues, such as elasticity, strength, and extensibility.^497,498^ This necessitates highly customized design and synthesis of hydrogels to cater to the demands of various application scenarios, including the regeneration of diverse types of tissues like skin, cartilage, or blood vessels.^499,500^ The degradation rate and manner of hydrogels are also significant, as they should degrade at a controlled rate conducive to tissue regeneration and eventual absorption or replacement.^230,501^ To foster cell proliferation and tissue repair, hydrogel-based artificial tissues must efficiently facilitate cell adhesion, growth, and differentiation. This process might necessitate refining the hydrogel’s internal composition or incorporating growth factors, cell-binding peptides, or additional bioactive compounds to augment their bioactivity. Furthermore, the stability and functionality of hydrogels in medical applications, including their physiological stability, performance retention post-implantation, and minimally invasive implantation techniques, warrant attention.

In the development and application of hydrogel-based biosensors, several key considerations are necessary to ensure the sensors’ efficacy and practicality. Firstly, for hydrogel sensors designed to detect low concentrations of biomarkers, it is imperative to integrate internal materials that enhance sensing capabilities. Another important factor is the physical and chemical stability of hydrogels, especially in scenarios of long-term implantation or continuous monitoring. This encompasses their response to variations in physiological conditions, such as changes in pH, temperature fluctuations, and potential enzymatic degradation.^502,503^ The mechanical properties of hydrogels, including elasticity, extensibility, and self-healing capabilities, must be tailored to their intended application to guarantee the durability and reliability of sensors in real-world applications. When designing hydrogel sensors for specific biomedical applications, precise control over release dynamics and drug delivery capacities are crucial considerations. For instance, in therapy monitoring or targeted treatments, hydrogels must possess the ability to precisely regulate the rate and quantity of drug release to achieve optimal therapeutic outcomes.^504^ Although hydrogels offer the potential for sensitive and specific detection of a wide range of biomarkers, developing a hydrogel system capable of efficiently capturing target molecules while resisting interference from biological matrices, such as proteins and cellular debris, remains a significant challenge.

### Hydrogel-mediated applications on non-cell therapy

#### Hydrogel-mediated drug delivery

##### Small molecules

The prevalent categories of small molecule pharmaceuticals are predominantly chemically synthesized, including a wide range of antimicrobial, antiparasitic, and antitumor agents.^505,506^ Specifically, in the context of chemotherapy, the variability in individual bioavailability post systemic administration and the severe adverse effects resulting from the off-targeted action of these agents significantly constrain their broader application. Moreover, the inherent hydrophobicity of conventional small-molecule chemotherapeutic agents necessitates the inclusion of co-solvents in vivo to enhance their bioavailability, which may introduce additional toxicities. For instance, substantial incorporation of ethanol to augment paclitaxel solubility has been implicated in central nervous system pathologies,^507^ while Cremophor-EL, another solubilizing agent for paclitaxel, is associated with severe hypersensitivity reactions.^508,509^ Thus, a versatile drug delivery system that enhances the bioavailability of small molecule drugs is paramount in advancing disease treatments.

As previously discussed, the capability for in-situ delivery and the physicochemical properties of hydrogels render them an exemplary platform for small molecule drug delivery. However, the challenge of burst drug release from the carrier due to the low molecular weight of small molecule drugs, leading to adverse effects from sudden concentration spikes, necessitates strategic utilization of hydrogels for this purpose. In response, researchers have developed peptide-based supramolecular hydrogels, which, through the conjugation of chemotherapeutic drugs to specific peptide sequences, form amphiphilic conjugates that trigger hydrogel formation in response to environmental stimuli (e.g., temperature, pH, or ionic strength changes).^510–513^ These hydrogels not only address the issue of premature drug release but also enhance drug solubility, thereby increasing the drug’s loadability and release, which in turn reduces the required total drug dosage.^514,515^ Furthermore, peptide hydrogels can be engineered to target specific cell membrane receptors, facilitating targeted drug delivery and minimizing systemic toxicity.^516^ For instance, Li and coworkers developed a hydrogel utilizing a pentapeptide (Phe-Phe-Arg-Gly-Asp) and glucosamine structure through covalent linkages. The RGD incorporation facilitated precise targeting of drug delivery towards tumor cells by binding to integrins, which are abundantly present on the surface of these cells.^517^ In another study, a supramolecular hydrogel, composed of D-amino acid residues, was engineered, where hydrogel formation was triggered enzymatically by alkaline phosphatase.^518^ The conjugation of Taxol to this D-peptide hydrogelator precursor improved Taxol’s solubility, thus enhancing its loading amount while preserving its antitumor activity. Moreover, the integration of D-amino acids into the peptide sequence markedly improved the hydrogel’s resistance to degradation by proteinase K in comparison to its L-amino acid counterpart, thereby offering a robust matrix for sustained drug release.^519^ Upon intratumoral injection, Taxol was directly delivered to and retained within the tumor site for up to 14 days, demonstrating enhanced antitumor effectiveness and minimal systemic side effects relative to its intravenous administration.^518^

The inherent hydrophilic characteristics of hydrogels pose challenges for the encapsulation and subsequent release of hydrophobic pharmaceutical drugs. Given this context, incorporating hydrophobic molecules into hydrogel networks serves as a typical strategy to provide binding sites for hydrophobic drugs, thereby enhancing compatibility with hydrophobic drugs. For example, cyclodextrin (CD), a class of macrocyclic oligosaccharides, is characterized by its outer hydrophilic surface and inner hydrophobic cavities, enabling the encapsulation of hydrophobic molecules while maintaining the hydrogel’s hydrophilicity.^520,521^ Thus, CD-based carriers have been extensively employed in the delivery of diverse pharmaceutical agents, even multi-drug delivery.^522–525^ For instance, Domiński and coworkers developed a supramolecular hydrogel utilizing host–guest complexes formed by pH-responsive micelle-derived PEG chains and α-CD to simultaneously deliver hydrophilic glycoconjugate and hydrophobic drugs doxorubicin.^526^ Its dynamic physical bonds imparted hydrogel’s shear-thinning properties, facilitating direct injection at the tumor site. Furthermore, the acidic TEMs triggered the hydrolysis of ketal bonds, allowing for the controlled disassembly of the gel matrix, releasing loaded anti-cancer agents, and thus inhibiting tumor growth. Compared to free drug administration, this hydrogel-based codelivery system not only demonstrated superior therapeutic outcomes but only reduced drug dosages, thus effectively diminishing systematic adverse effects.

Another approach to enhance the incorporation and dispersion of hydrophobic drugs within hydrogels involves the utilization of nanoparticles.^527^ By encapsulating the drugs in nanoparticles and embedding them within a hydrophilic hydrogel matrix, this strategy not only facilitates improved drug loading but also potentially enhances cellular drug uptake *via* endocytosis and circumvents drug efflux mechanisms mediated by ABC transporters, thereby attenuating drug resistance.^528,529^ Furthermore, the encapsulation of drug-laden nanoparticles in hydrogels can minimize systemic distribution and thereby mitigate toxicity.^530^ For instance, gefitinib—a tumor-targeting drug—experiences challenges in homogeneous distribution and drug loading within hydrogels due to its lipophilic nature. Karimi and coworkers devised a formulation comprising gefitinib-loaded cellulose acetate butyrate nanoparticles (Gnb-NPs), which were subsequently integrated into thermosensitive chitosan/β-glycerophosphate hydrogels, creating drug-loaded depots for intratumoral administration in breast cancer-bearing mice.^531^ Relative to free gefitinib or its direct incorporation into hydrogels, the Gnb-NPs hydrogel demonstrated superior antitumor efficacy by localizing therapy, attributed to the hydrogel’s capacity to extend the retention period of nanoparticles near the tumor site, thus enhancing the penetration of nanoparticles into the tumor. Additionally, the Gnb-NPs hydrogel exhibited the lowest incidence of organ-related toxicities.

Beyond the encapsulation of hydrophobic drugs, sophisticatedly designed hydrogels possess the capability to facilitate multiple drug delivery, especially those that are sequential or spatiotemporal, thus optimizing drug effectiveness and ensuring targeted therapeutic action. This approach is particularly crucial for the management of intractable tumors, where monotherapy often proves insufficient, and the concurrent administration of multiple drugs may diminish the efficacy of combination treatments due to drug interactions and excessive systemic accumulation. Consequently, the innovation of hydrogels with phased drug release capabilities is instrumental in maintaining continuous treatment and significantly enhancing therapeutic efficiency. For chemotherapeutic agents such as paclitaxel, doxorubicin, and cisplatin, an orchestrated release is attainable by leveraging their differential affinity to water and varying diffusion rates, matching with the hydrogel matrix’s hydrolysis rate or specific stimuli-responsive degradation mechanisms.^532–534^

**Fig. 9:**
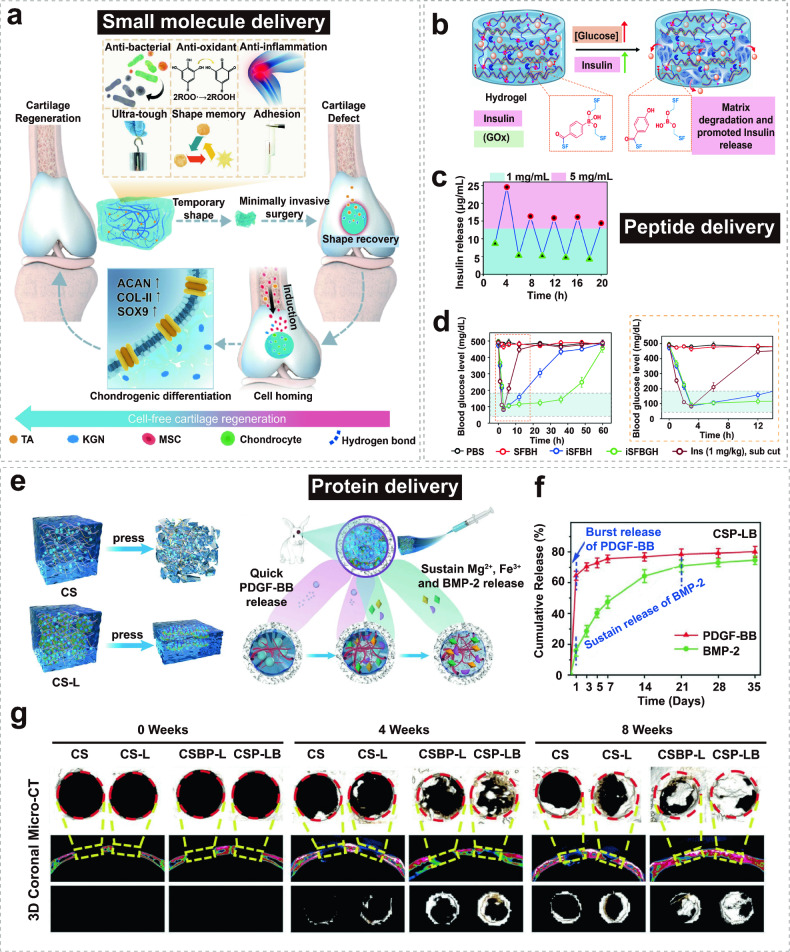
Representative examples of hydrogel-mediated delivery systems for small molecules (**a**), peptides (**b**–**d**), and proteins (**e**–**g**). **a** Schematic of a hydrogel crosslinked through multiple hydrogen bonds, presenting fast shape memory to facilitate minimally invasive implantation and release of small molecule drugs for homing endogenous BMSCs to promote cartilage regeneration. Reproduced from ref. ^535^ **b** Schematic of a glucose-sensitive hydrogel designed for functional insulin delivery. **c** Insulin release profile from the gel, demonstrating a pulsatile release pattern. **d** Blood glucose levels of diabetic rats after subcutaneous administration. **b**–**d** reproduced from ref. ^549^ **e** Schematic of a CS hydrogel enhanced with LDH nanosheets to improve its mechanical properties and support a controlled sequential release of growth factors, promoting bone regeneration. **f** Release profiles of PDGF-BB and BMP-2 from the hydrogel. **g** Micro-computed tomography (Micro-CT) images showing skull defect repair induced by the hydrogel. **e**–**g** reproduced with permission from ref. ^572^ Copyright 2022, John Wiley and Sons. Adobe Illustrator was used to generate this figure

Furthermore, the strategic sequential release of drugs *via* hydrogels also holds profound implications for tissue repair, aiming to mimic the sequential biochemical cues inherent in physiological regenerative processes. For instance, Yang and coworkers devised a hydrogel with multiple hydrogen-bond crosslinking, loading tannic acid and Kartogenin through a polyaddition reaction to promote cartilage regeneration (Fig. 9a).^535^ These hydrogen bonds not only endowed stable mechanical properties and notable shape memory features which facilitated minimally invasive surgery but also enabled phased drug release.^535^ Due to the divergent water affinities of the encapsulated drugs, the highly hydrophilic tannic acid was preferentially released post-implantation, mitigating inflammation and clearing excess ROS. This initial release shifted the microenvironment towards one favorable for the homing of BMMSCs. Subsequent hydrogel degradation allowed for the sustained release of the hydrophobic Kartogenin, promoting the differentiation of BMMSCs into chondrocytes and thus accelerating cartilage regeneration.

The aforementioned examples underscore the substantial benefits of employing hydrogels as delivery systems for small molecule agents. This hydrogel-based approach not only augments drug loading capacity and sustains drug release but also ensures the maintenance of effective drug concentrations at the target site post in-situ injection, thereby diminishing systemic toxicity and adverse effects. Additionally, hydrogels’ capacity for multi-drug delivery and sequential drug release further enhances the therapeutic potential of small molecule drugs, particularly in combination therapies.

##### Peptide drugs

Peptide drugs are characterized for their superior biological activity and safety over small molecule drugs, and a simpler, more cost-effective production compared to protein-based drugs. Nonetheless, the application of peptides still faces several challenges, including susceptibility to enzymatic degradation within the body, rapid clearance by the immune system, and a brief plasma half-life. Traditional administration methods, including intravenous, subcutaneous, or intramuscular injections, often necessitate multiple daily doses to maintain effective drug levels, leading to potential issues with patient compliance.^536,537^ Transdermal delivery depends heavily on the drug’s penetration capabilities; however, the diffusion of peptide drugs through transcellular or paracellular pathways is impeded by their larger molecular weight compared to small molecule drugs, complicating membrane transport and absorption.^538^ In addition, their vulnerability to the stomach’s acidic environment and digestive enzymes restricts oral administration to only a few peptides, such as cyclosporine and desmopressin. Despite attempts to enhance oral bioavailability through protease inhibitors, outcomes remain modest and may introduce additional safety concerns.^539,540^

In this context, hydrogels stand out as a viable strategy to overcome these obstacles. Chemically tailored hydrogels, such as hyaluronic acid variants modified with cholesterol groups, demonstrate enhanced peptide affinity, thereby increasing the drug loading.^541^ Moreover, hydrogels, through the integration of responsive elements, provide a protective barrier against detrimental environmental factors like gastric acids, ensuring peptide release under optimal conditions and maintaining drug efficacy. Illustratively, Yang et al. devised hydrogel-based microparticles from carboxymethyl β-cyclodextrin-grafted carboxymethyl, presenting a novel method for oral insulin delivery.^542^ These hydrogel microparticles, with their loose pores, provided ample storage for insulin and used their hydrophilic groups and porous network to absorb water, inducing gel swelling and rapidly releasing insulin. Notably, the incorporation of carboxymethyl chitosan (CMC) shielded the drug’s activity against harsh gastric acids. At lower pH levels, such as in gastric fluid (pH = 1.2), CMC’s amino groups protonated, fostering intermolecular hydrogen bonding that consolidated intermolecular forces and inhibited swelling, thus preventing premature drug release.^543^ Conversely, at a pH of 6.8 (mimicking intestinal fluid), the hydrogel’s swelling behaviors significantly enhanced, facilitating insulin release and absorption in the intestinal tract.^542^

More critically, in some cases, on-demand drug release seems to be indispensable, which can be achievable through the strategic incorporation of responsive elements that trigger hydrogel degradation, deformation, or swelling changes. Still concerning insulin delivery, such on-demand release mechanisms are crucial in preventing hypoglycemia caused by abrupt or undesired insulin discharge. To imitate pancreatic function and automate insulin release in accordance with blood glucose concentration, an array of glucose-responsive delivery systems has been designed, showcasing the adaptability and potential of hydrogels in regulated drug delivery. Li and coworkers developed a pH-responsive peptide hydrogel loaded with glucose oxidase (GOx), catalase, and insulin.^544^ Elevated blood glucose levels triggered glucose diffusion through the hydrogel matrix, reacting with GOx to catalyze glucose turning into gluconic acid and hydrogen peroxide (H_2_O_2_), thereby reducing the local pH. When the pH dropped below the pKa of lysine/ornithine side chains, that would induce electrostatic repulsion, disassembling the hydrogel and liberating insulin. In vivo experiments demonstrated that this smart hydrogel platform presented long-term efficacy in glucose regulation in diabetic mice.

A similar closed-loop artificial pancreas system can be realized through phenylboronic acid (PBA)-based hydrogels. PBA, as a glucose-sensitive agent, is capable of reversible binding with 1,2- or 1,3-cis-diols of glucose, forming dynamic boronic ester bonds that modulate the network’s hydrophilicity.^545^ This responsiveness facilitates matrix swelling or degradation, supporting sustained insulin release.^546,547^ Moreover, these dynamic bonds can endow this kind of gel with shear-thinning and self-healing properties, crucial for injectable applications and forming a durable drug depot for regulated insulin delivery.^548^ Maity’s team devised a glucose-responsive, self-regulating hydrogel composed of PBA-functionalized silk fibroin protein, for functional insulin delivery.^549^ The incorporation of GOx within the hydrogel structure conferred glucose sensitivity. Upon detection of elevated blood glucose levels, the hydrogel’s responsive mechanism—through the interaction of glucose with embedded elements—generates H_2_O_2_, leading to the cleavage of dynamic boronic ester bonds. This reaction facilitates a pulsatile release of insulin. In vivo experiments on type 1 diabetic Wistar rat model demonstrated that this hydrogel could effectively maintain diabetic glucose levels to normal physiological conditions over a period of 36 hours, highlighting the gel’s potential for providing a sustained and efficacious insulin delivery (Fig. 9b–d).^549^

##### Protein drugs

Protein therapeutics, similar to peptide drugs, encounter the same issues such as rapid in vivo elimination and low bioavailability. To sustain therapeutic concentrations within the body, an increase in dosage frequency or quantity is often required, potentially imposing financial strains on patients.^550^ Moreover, certain protein drugs require optimal reaction conditions for sustained activity and efficacy, with systemic administration potentially resulting in adverse effects that could restrict their wider therapeutic applications.^551^ For instance, hyaluronidase PEGPH20 aims to deplete hyaluronic acid in the pancreatic ductal adenocarcinoma matrix, serving as an adjunct to enhance the permeation of chemotherapeutic agents like gemcitabine and paclitaxel. However, phase III clinical trial outcomes for this approach have been underwhelming.^552^ Consequently, the evaluation of protein drug delivery methods must account for the simplicity and cost-effectiveness of production, alongside considerations of protein activity, bioavailability, and potential toxicity. Hydrogel-based controlled-release delivery systems offer a promising avenue for reducing dosage frequency and extending dosing intervals, all while preserving drug efficacy.

*Antibodies*: The in vivo utilization of antibodies can be hindered by their instability and potential for inducing adverse reactions. Structurally, antibodies consist of two domains: the antigen-binding fragment (Fab), which is responsible for antigen recognition, and the crystallizable fragment (Fc), which mediates immune effector functions. These domains are conjoined by disulfide bonds that are susceptible to being broken in environments rich in glutathione (GSH), such as the TMEs, potentially leading to the dissociation of the antibody structure and consequent therapeutic failure. In addition, the manufacturing complexity and associated costs with antibody drugs underscore the need for more efficient delivery systems that can stabilize antibodies thus ensuring therapeutic efficacy. For example, Li et al. successfully engineered a thermo-responsive hydrogel, poly (N-isopropylacrylamide-co-methacrylic acid), conjugated with IL-6-specific antibodies. This innovative hydrogel not only preserved the antibody’s function post-application but also inhibited its premature dissemination, markedly diminishing IL-6 concentrations during cytokine release syndrome triggered by CAR-T cell therapy.^553^

Additionally, systemic administration of antibodies often leads to broad drug distribution throughout the body, necessitating increased dosages to achieve therapeutic concentrations at target sites and heightening the risk of off-target effects and systemic adverse reactions. For example, ICBs, such as cytotoxic T-lymphocyte-associated protein-4 (CTLA-4), PD-1, and PD-L1 antibodies, are designed to modulate immune checkpoint pathways. However, since these pathways are also present in normal tissues, the use of ICBs can inadvertently lead to the immune system attacking non-tumor tissues, triggering a spectrum of adverse reactions, including fatigue, rash, diarrhea, and nausea.^554^ In response to these challenges, hydrogel-based drug delivery platforms have been developed, which see the great potential of minimizing serum antibody concentrations, thereby mitigating systemic side effects.^555^ For instance, a thermo-responsive polypeptide hydrogel encapsulating dual ICB antibodies for the treatment of B16F10 melanoma demonstrated controlled release through protease-mediated enzymatic degradation within the extracellular matrix.^556^ This slow release ensures that the antibodies can be localized around the targeted site, restricting ICB antibodies to the vicinity of the tumor and minimizing systemic dissemination. Similarly, another investigation employed an in-situ crosslinking hydrogel for extended delivery of anti-CTLA-4 and anti-PD-1 antibodies, evidencing the hydrogel’s advantage in mitigating side effects.^557^ Intratumoral delivery via this gel system ensured sustained antibody bioavailability and lower hepatotoxicity, as indicated by the significantly reduced serum levels of aspartate aminotransferase compared to direct antibody administration.

Moreover, the intricate microenvironment of pathological sites, such as the TMEs, may attenuate antibody reactivity. Hydrogels offer a versatile platform for the encapsulation and controlled release of drug combinations—such as antibodies in concert with chemotherapeutic agents or immunoadjuvants—to enhance therapeutic outcomes through synergistic effects. Cheng *et al*. have innovated a single-dose, thermo-responsive hydrogel system (NvIH) to encapsulate immune ICB antibodies and synthetic polymer nanoparticles. These nanoparticles were loaded with tripartite immunostimulatory agonists targeting Toll-like receptors 7, 8, and 9 (TLR7/8/9) alongside a stimulator of interferon genes (STING).^558^ This hydrogel system was designed to rapidly gelation at body temperature directly within the tumor site, thereby enhancing the duration of drug retention and facilitating a controlled release of the therapeutic agents from the NvIH matrix to specifically address the dispersion of off-target immunostimulants. The integration of cationic polymers within the hydrogel aided in the intracellular delivery of these immunostimulants, broadening the spectrum of activatable immune cell subsets and activating multiple signaling pathways, including those leading to pro-inflammatory responses. This strategy effectively diminished immunosuppression within the TMEs. Compared with non-synergistic treatment, this combination—the single-dose NvIH hydrogel—presented substantial tumor treatment efficacy and significantly elevated fractions of complete tumor regression. Similarly, a unique thermosensitive hydrogel, composed of gelatin and Pluronic® F127, was developed for delivering a nitric oxide donor (S-nitrosoglutathione, GSNO) and ICB antibodies targeting CTLA-4, offering a potent approach to anti-tumor immunotherapy.^559^ The dual delivery system exploited the capability of GSNO to activate DCs, concurrently with CTLA-4 inhibitors intervening in the function of regulatory T cells to weaken their suppression of dendritic cells, thereby offsetting the undesired stimulation of CTLA-4-expressing immunosuppressive cells which can negatively impact anti-tumor treatment. Additionally, by leveraging the hydrogel’s temperature-sensitive gelation and TME-responsive degradation properties, this strategy ensured prolonged retention of the therapeutic agents at the tumor site, resulting in a synergistic and systemic anticancer response in a melanoma model that is resistant to monotherapy with either GSNO or CTLA-4 blockade.

In the context of dense tumor matrices, the large molecular size of antibody drugs can impede their penetration and therapeutic action. Hydrogel systems, when engineered to co-deliver matrix-degrading enzymes, can enhance the permeation of these drugs, thus potentially improving their therapeutic impact.^560^ For example, in the context of breast cancer, the dense, collagen-rich ECM significantly obstructs the infiltration of therapeutic antibodies such as trastuzumab. A thermosensitive hydrogel based on PLGA-PEG-PLGA was employed for the peritumoral injection in human epidermal growth factor receptor 2 (HER2)-positive BT474 tumor-bearing mice, facilitating a prolonged release of trastuzumab and collagenase over two to three weeks due to the gradual breakdown of the polyester components.^561^ The co-delivery of collagenase notably diminished the collagen density within the solid tumor ECM, enhancing the deep tissue penetration of antibodies and thereby amplifying both the interstitial transport and the overall anti-tumor effectiveness of the therapy.

*Growth factors*: Growth factors, acting as pivotal signaling molecules, orchestrate cellular processes such as proliferation, differentiation, and migration by interacting with specific transmembrane receptors, significantly impacting tissue development and repair.^562–564^ The application of growth factors for tissue repair and regeneration necessitates a prolonged duration to synchronize with the physiological cycles of tissue healing. Consequently, the development of hydrogel systems capable of maintaining the bioactivity of growth factors and facilitating sustained release offers an optimal delivery mechanism.^565,566^ Owing to the charged nature of many protein-based growth factors, it enables the exploitation of electrostatic interactions with hydrogels to extend the release duration of growth factors. Notably, heparin-based hydrogels, characterized by their significant anionic charge and diverse sulfation patterns, present a compelling approach for the delivery of various growth factors such as fibroblast growth factors (FGFs), hepatocyte growth factors (HGFs), endothelial growth factors (EGFs), and platelet-derived growth factors (PDGFs).^567,568^ Studies have demonstrated that the inclusion of thiolated heparin in hydrogels significantly amplifies retention of growth factors, with one such hydrogel exhibiting an elevenfold increase over 28 days compared to its counterpart without thiolated heparin.^569^ Similarly, Fu and coworkers have demonstrated that the incorporation of heparin into the hydrogel matrix not only extended the diffusion of endothelial growth factors (EGFs) but also preserved their biological activity, thus underscoring the promise of heparin-based hydrogels as vehicles for growth factor delivery.^570^

Moreover, beyond the paradigm of sustained release, the temporal sequencing in the release of growth factors is of paramount importance, especially in the context of cell differentiation and tissue regeneration. Such biological processes are contingent upon a well-orchestrated series of stimuli that mimic physiological regenerative sequences.^571^ Hydrogels, with their inherent versatility, offer a platform that can be tailored through various strategies (e.g., different encapsulation techniques) to modulate the sequential delivery of growth factors, thereby catering to the intricate demands of regenerative therapeutics.^161^ Lv et al. developed an injectable chitosan/silk fibroin (CS) hydrogel incorporating MgFe-layered double hydroxide (LDH) nanosheets to enhance the gel’s mechanical properties and to permit a sequential release of growth factors, conducive to bone regeneration.^572^ Within this system, PDGF-BB exhibited an initial burst release by direct encapsulation within the gel, while BMP-2 was modulated for prolonged release by its conjugation to LDH nanosheets *via* electrostatic interactions facilitated by chondroitin sulfate. This sequential release paradigm can promote angiogenesis followed by osteogenesis,^573^ ultimately resulting in significantly enhanced bone regeneration efficiency in a skull defect model of New Zealand rabbits (Fig. 9e-g).^572^

Furthermore, the spatial configuration and scale of compartments within hydrogel constructs, loaded with disparate growth factors, also offer an avenue for sequential release mechanisms. The advent of 3D printing technology particularly propels this approach, allowing the precision fabrication of hydrogel scaffolds with intricate designs that enable the sequential release of multiple growth factors.^574^ For instance, multi-layered cylindrical shells constructed by 3D printing technology permit fine-tuned spatial control over the sequence of growth factor release,^569^ underscoring the significant potential of this technology in the field of regenerative medicine.

*Enzymes*: Enzymes, as catalytic proteins, are integral in various biosynthetic, transformational, and degradative processes within biological systems. However, the efficacy of natural enzymes largely depends on maintaining optimal conditions; deviations in temperature, pressure, or pH can result in their inactivation and consequent therapeutic ineffectiveness.^575^ Ensuring the stability and activity of enzymes during in vivo delivery is a critical challenge, one that hydrogel systems have been developed to address.

Take lysostaphin as an example. Lysostaphin is a typical enzyme with potent bacteriolytic properties against staphylococcal infections, yet its therapeutic application has been historically constrained by the absence of an efficient delivery mechanism.^576^ Johnson et al. have innovatively engineered an injectable hydrogel from 4-arm PEG functionalized with terminal maleimide groups to physically encapsulate lysostaphin.^577^ Notably, this hydrogel maintained the enzymatic activity of lysostaphin for over two weeks and demonstrated consistent performance with freshly prepared enzyme samples. Moreover, the hydrogel-mediated delivery of lysostaphin even exhibited superior efficacy in treating biofilms compared to its soluble counterpart, likely attributed to the hydrogel’s capacity for the sustained release of lysostaphin through passive diffusion and its degradation by proteases. When administered locally at the site of femur fracture, this delivery system markedly increased lysostaphin levels at the infected site, effectively eradicating orthopedic infections caused by *S. aureus*. Further, the simultaneous delivery of lysostaphin and BMP-2 through this hydrogel not only eliminated *S. aureus* infections but also promoted the regeneration of functional bone, thereby facilitating the repair of defects without eliciting local or systemic adverse effects.^578^

##### Gene drugs

Gene therapy, a forefront medical technology, introduces specific exogenous genes into human target cells. These genes have the potential to correct genetic anomalies, as well as enhance or suppress the function of specific genes through replacement, integration, or regulation within the target cells, offering therapeutic approaches for diverse diseases. Its high degree of customization stands as a significant advantage. Tailoring treatment to the patient’s genetic and disease profile, scientists can select or adapt genes for personalized treatment strategies, aiming for the best therapeutic results. The gene therapy sector is marked by a wide variety of developing and applied product types, including, but not limited to, nucleic acid-based drugs (DNA and RNA), viral vector-based gene therapy medicines, and bacterial vector-based therapeutic agents.^579^ The aim of selecting and designing these strategies is the efficient and safe delivery of therapeutic genes to the target cells. Strategies using viral vectors leverage the inherent infective capabilities of viruses to precisely deliver genes. Conversely, lipid nanoparticle-based delivery systems encase messenger RNA (mRNA) vaccines or other therapeutic molecules in minuscule lipid particles, utilizing advanced nanotechnology to improve bodily stability and intracellular delivery efficacy.^580,581^

Nucleic acid-based therapeutics exhibit inherent chemical and physical instabilities, rendering them susceptible to various endogenous factors (such as primary structure alterations, chemical modifications of bases, and the presence of small molecular residues) and exogenous factors, including pH, buffer concentration, metal cations, and reactive oxygen species.^582^ Given the challenges posed by nucleases and the necessity for active and targeted delivery of nucleic acids, the selection of suitable materials for the controlled orientation of gene editing products is crucial. This not only ensures the stability of therapeutic agents but also targets diseased cells, thereby enhancing the safety profile of the treatment. Hydrogels, with their versatile physicochemical properties, emerge as a promising candidate in this context. Their ability to form protective matrices around nucleic acids can significantly mitigate the impact of both endogenous and exogenous destabilizing factors.^583^ Moreover, hydrogels can be engineered to provide targeted delivery through modifications that recognize specific cellular markers, addressing the critical aspect of ensuring active transport and migration to the intended site of action.^584^

In the dynamic field of therapeutic interventions, combining gene therapy with other therapies has attracted significant interest. This interdisciplinary approach requires the identification of materials and methods capable of simultaneously delivering these treatments. Hydrogels, with their adjustable degradation rates and ability to encapsulate various therapeutic molecules, are ideally suited for this task.^1^ They can be engineered to release multiple agents in a controlled fashion, potentially enhancing the efficacy of each treatment modality. Additionally, while many delivery systems emphasize sustained drug release, the potential for adverse effects due to prolonged release necessitates careful consideration.^585^ Hydrogels address this issue by incorporating stimuli-responsive elements that can adjust or cease drug release in response to specific physiological signals, thus minimizing the risk of side effects from extended drug exposure and underscoring the versatility and critical importance of hydrogels in the targeted delivery of nucleic acid-based therapeutics.^586^

**Fig. 10:**
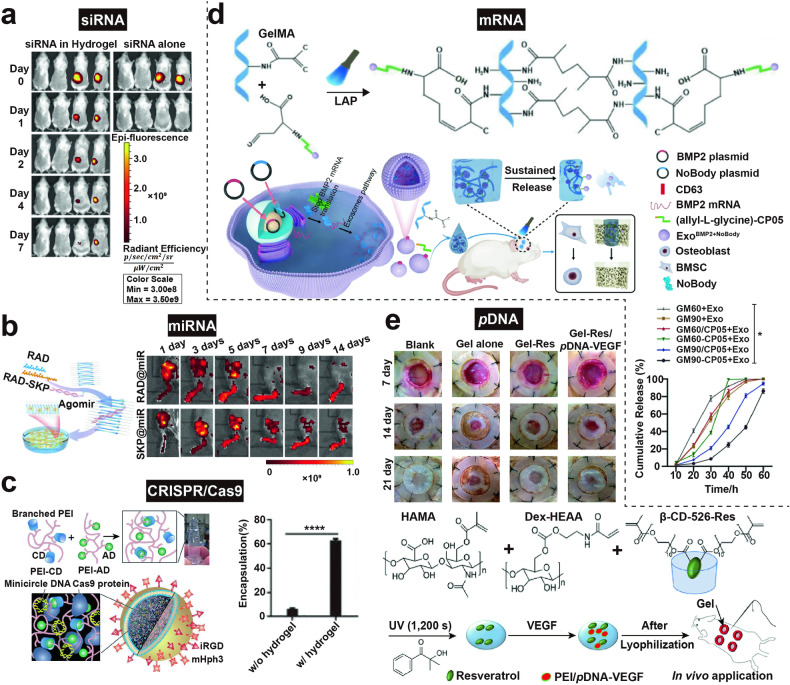
**a** Following subcutaneous injection of Cy5-siRNA-loaded hydrogel in mice, the fluorescent signal from chitosan hydrogel-incorporated siRNA can be sustained for up to 7 days, whereas the fluorescent signal from RANK siRNA used alone diminishes in less than 1 day, and the cumulative release curve of siRNA from the chitosan hydrogel/siRNA in vitro and in vivo. Reproduced from ref. ^583^ **b** Self-assembled nanofiber hydrogels composed of RAD and SKP peptides, internally loaded with agomir-29b-5p (cholesterol-modified mir-29b-5p), were injected into the joints. Schematic representation of in vivo imaging of Cy5.5-labeled agomir-29b-5p in mouse joints at 1, 3, 5, 7, 9, and 14 days post-injection of the hydrogel. Reproduced from ref. ^586^ **c** The formation of PEI hydrogel through CD-AD mediated host-guest interactions, with the core and shell of LHNPs composed of PEI hydrogel and DOTAP liposomes, respectively. Hydrogel-core systems demonstrate an enhanced encapsulation efficiency of CRISPR/Cas9 up to 62.8%. Reproduced with permission from ref. ^584^ Copyright 2017, John Wiley and Sons. **d** Schematical showing the preparation of ExoBMP2+NoBody-loaded GelMA and its effect on bone regeneration. And the sustained release effect of GelMA-CP05 + Exosome hydrogel, with GM-90-CP05 + Exosome hydrogel exhibiting the slowest degradation rate. Reproduced from ref. ^605^ **e** Resveratrol and VEGF DNA plasmid were integrated into the scaffold to confer anti-inflammatory and pro-angiogenic effects. And representative images of wounds treated with Gel alone, Gel-Res, and Gel-Res/pDNA-VEGF for 21 days. Reproduced with permission from ref. ^610^ Copyright 2019, Elsevier. Adobe Illustrator was used to generate this figure

*siRNA:* Small interfering RNA (siRNA), composed of 20–25 base pairs of double-stranded RNA, selectively targets and degrades transcribed mRNA by recruiting specific enzymes. In cancer cells, siRNA disrupts the mRNA from oncogenes, effectively diminishing protein synthesis that facilitates tumor growth. However, siRNA faces challenges such as degradation by endogenous nucleases and rapid elimination due to its small size, leading to potential renal clearance. To overcome these obstacles and improve cellular uptake, the use of protective carriers for targeted delivery is essential. Hydrogel systems, in particular, have shown promise in achieving targeted siRNA delivery and controlled release, thereby influencing gene expression precisely. An example is the use of chitosan hydrogels for delivering NF-κB receptor activator (RANK) siRNA in a periodontitis mouse model, where in vivo studies demonstrated that fluorescent signals from the siRNA encapsulated in chitosan hydrogels can be sustained for up to 7 days—significantly longer than the less than one-day longevity of free RANK siRNA (Fig. 10a).^583^ This chitosan hydrogel could serve as a suitable reservoir and carrier for locally sustained delivery of siRNA in chronic inflammation therapy.

Chemical conjugation, such as covalent bonding, significantly stabilizes siRNA integration within hydrogels, minimizing nonspecific release and enhancing therapeutic effectiveness. Kim^587^ engineered siRNA targeting noggin—an inhibitor of bone morphogenetic protein signaling—by attaching it to methyl acrylate groups with disulfide bonds for covalent linkage to hydrogels. This method resulted in an impressive initial incorporation rate of 95.3% for siRNA, surpassing the 72.7% achieved through mere physical mixing. Chemical conjugation markedly extended the release duration of siRNA and mitigated the burst release effect. For instance, on the first day, the release rate was only 24% in the chemically conjugated group compared to 70% in the simple mixing group. This controlled release is attributed to the gradual degradation of disulfide bonds within the hydrogel, ensuring a steady release over time.^587^ Evidence suggests that the chemical linkage of siRNA to hydrogel matrices substantially improves its stability and release control in vivo, marking a potent delivery mechanism for siRNA-based gene therapy.

Moreover, the inherent negative charge of compounds like siRNA poses challenges in crossing similarly charged cell membranes, hampering cellular uptake. A proven strategy to overcome this involves incorporating cations into hydrogels to neutralize the charge, thereby encapsulating siRNA for effective delivery and cell entry.^6,588,589^ Leber^590^ developed cationic nanogel particles loaded with negatively charged siRNA for treating liver fibrosis. The nanogel particles feature a cationic core with multiple binding sites, facilitating siRNA complexation through multivalent electrostatic interactions. This configuration not only ensures the particles’ stability but also significantly stabilizes the encapsulated siRNA.^590^ Consequently, this positively charged hydrogel system emerges as an efficient siRNA carrier, offering a promising approach for enhancing siRNA cellular uptake.

*miRNA*: MicroRNAs (miRNAs), which are 18–22 nucleotide long double-stranded RNA molecules, play a crucial role in post-transcriptional regulation by base-pairing with mRNA and suppressing its expression. The therapeutic use of miRNAs requires careful consideration of potential off-target effects that may lead to genetic imbalances and the challenges associated with repeated dosages due to their inherently low stability. Self-assembling, injectable hydrogels offer a promising solution for localized delivery, providing sustained release of miRNAs to overcome these hurdles.^591^ For example, the self-assembling peptide hydrogel SKPPGTSS, with its nano-fibrous, porous structure that resembles the extracellular matrix, is particularly effective for the encapsulation and delivery of miR-29b-5p.^586^ The presence of positively charged lysine residues in SKPPGTSS facilitates a strong affinity for miR-29b-5p through hydrogen bonding and electrostatic interactions, ensuring the miRNA’s prolonged bioactivity and sustained release. Zhu^586^ demonstrated that this hydrogel system could continuously release miR-29b-5p for up to 40 days in vitro. When administered in vivo, it targeted the inflamed joint microenvironment, where the encapsulated miR-29b-5p was active for up to 14 days (Fig. 10b). This prolonged presence significantly contributes to inhibiting chondrocyte senescence, enhancing chondrogenic differentiation, and preserving cartilage homeostasis. Such findings underscore the system’s potential in effectively managing osteoarthritis and mitigating the issues related to the repeated administration of unstable miR-29b-5p oligonucleotides.

Another noteworthy hydrogel exploits host-guest chemistry, utilizing interactions between β-cyclodextrin (β-CD) as the host and adamantane (AD) as the guest, within modified polymers to create supramolecular hydrogels.^592^ These interactions enable the CD to effectively bind polymer chains, forming robust hydrogel matrices. By conjugating CD with hyaluronic acid, the hydrogel’s affinity for hydrophobic miRNAs is significantly enhanced, resulting in slower release rates and extended-release durations. Wang’s research demonstrates that incorporating cholesterol-modified miRNAs for myocardial infarction treatment into these hydrogels shows that cholesterol’s binding to CD slightly impacts the hydrogel’s erosion and mechanical properties. However, it significantly extends the miRNA release period to over three weeks in vitro, surpassing the duration achieved without this modification.^593^ The extended-release can be attributed to the dynamic nature of CD-AD interactions and the electrostatic repulsion between negatively charged HA and miRNAs. Furthermore, cholesterol’s modification increases the miRNA’s volume and, due to its hydrophobic nature and tendency to self-aggregate, aids in retaining miRNAs within the hydrogel network, thereby prolonging their release.^593^

*CRISPR/Cas9*: Clustered Regularly Interspaced Short Palindromic Repeats (CRISPR) and its associated protein, Cas9, have revolutionized genetic engineering, offering targeted modifications such as gene knockout, insertion, and sequence alterations. The ubiquity and short length of CRISPR/Cas9 target genes across the genome afford it a broader range of applications than alternative methods.^594^ This versatility facilitates widespread research across microorganisms, plants, and animals, tailoring genes to meet human needs, including the precise correction of disease-causing gene mutations.^595–598^ Nonetheless, the propensity for CRISPR/Cas9 to engage off-target sequences raises concerns about editing fidelity, potentially leading to the undesirable modification of functional genes or incorrect repair of disease-causing genes.^599^ To mitigate these risks, researchers are investigating targeted delivery systems, including the use of hydrogels, to enhance specificity and reduce off-target interactions. Although adeno-associated viruses are commonly employed for CRISPR/Cas9 delivery, their use is limited by immunogenicity and integration-related genotoxicity.^600^ Hydrogels, characterized by their mesh-like architecture, offer a promising alternative, providing abundant drug-binding sites for increased loading efficiency and protection against nuclease-mediated degradation.^601^ Their superior biocompatibility minimizes cytotoxicity and facilitates cellular entry of CRISPR/Cas9. Moreover, stimulus-responsive hydrogels can fine-tune the delivery of CRISPR/Cas9, ensuring precision editing at desired sites and minimizing unintended alterations.

One such non-cationic DNA-crosslinked nanogel, achieved by incorporating Cas9/single guide RNA (sgRNA) complexes into DNA-grafted poly(ε-caprolactone) brushes (DNA-g-PCL) and stabilizing through DNA hybridization, effectively encapsulates the Cas9/sgRNA complexes. This structure demonstrates remarkable nuclease resistance.^601^ Another innovative method uses liposome-templated hydrogel nanoparticles (LHNPs), which employ a cationic 1,2-dioleoyl-3-trimethylammonium-propane chloride (DOTAP) lipid shell, recognized for its gene delivery efficacy, safety, and broad clinical applicability.^602^ Despite its advantages, DOTAP liposomes alone have shown a limited Cas9 protein encapsulation efficiency of only 6.3%. To address this limitation, Chen et al. ^584^ introduced polyethylenimine (PEI) hydrogels within the LHNPs’ core to enhance the encapsulation efficiency, preserving the activity of the Cas9 protein. This core-shell architecture significantly improves CRISPR/Cas9 encapsulation efficiency to 62.8%, facilitating efficient delivery (Fig. 10c).^584^ This delivery system has been successfully applied in targeted gene therapy for mouse brain tumors, demonstrating significant gene suppression both in vitro and in vivo, and suggesting its potential for advanced cancer gene therapy applications.

The utilization of hydrogels as CRISPR/Cas9 delivery platforms holds considerable promise for enhancing the precision of gene editing. These innovative systems provide a protective environment against nucleolytic degradation for Cas9/sgRNA complexes, thereby improving the accuracy and efficacy of gene targeting. Developments in non-cationic DNA-crosslinked nanogels and liposome-templated LHNPs illustrate the capability for tailored design approaches to boost CRISPR/Cas9 packaging efficiency and preserve its functional activity. Nonetheless, the use of hydrogels as CRISPR/Cas9 delivery vehicles still faces several challenges. Ensuring the accuracy of CRISPR/Cas9 delivery to minimize off-target effects remains a crucial issue, which includes developing more precise targeting mechanisms and strategies to reduce unintended editing.^603^ Moreover, enhancing the encapsulation efficiency of CRISPR/Cas9 within hydrogel systems and maintaining its biological activity are current research focuses. Additionally, the immunogenicity and biocompatibility of hydrogels are essential factors to consider for their safe long-term application. Future research directions will involve developing novel hydrogel materials and configurations to further improve the stability, encapsulation efficiency, and targeting accuracy of CRISPR/Cas9 delivery systems. Furthermore, exploring stimulus-responsive hydrogels that can trigger the release of CRISPR/Cas9 under specific physiological conditions offers spatial and temporal control for precise gene editing.

*mRNA:* mRNA technologies are pivotal in tissue engineering, opening new therapeutic pathways by fostering cell differentiation and protein synthesis.^604^ The effectiveness of these technologies hinges on the precise functioning of mRNA within specific cellular structures and sites, necessitating the creation of delivery systems that ensure localized and sustained mRNA release. To this end, researchers have developed delivery vehicles that emulate the intracellular microenvironment to maintain mRNA’s biological activity and stability. Material selection for mRNA delivery often favors polymers that exhibit electrostatic neutrality, such as PEG and dextran, alongside cationic polymers that promote mRNA stability via self-assembly.^449^ These materials are crucial for the efficient encapsulation of anionic mRNA, facilitating its controlled release in the body. Furthermore, hydrogel systems adeptly address off-target concerns associated with nucleic acid transport, ensuring accurate mRNA delivery to intended cells and enhancing therapeutic efficacy.^604^ Therefore, hydrogels as mRNA delivery carriers not only increase the bioavailability of therapeutic agents but also improve treatment precision and safety.

Yang and his team engineered exosomes enriched with BMP-2 mRNA, incorporated into GelMA hydrogels to achieve sustained release. Methacrylated phage peptide CP05 was integrated to enlarge pore sizes, creating a porous structure (GM-90-CP05) that serves as an efficient scaffold for BMP-2 mRNA exosome delivery. This enhanced system demonstrated increased mRNA capacity, slower degradation, and extended release, promoting osteogenic protein expression and supporting durable bone regeneration in vivo (Fig. 10d).^605^ In parallel, a hydrogel-based system was developed to protect mRNA vaccines targeting TMEs, significantly boosting immune responses against tumors. Modular hydrogels composed of methacrylate encapsulate exosomes with GM-CSF mRNA, preserving mRNA integrity and activity for tumor vaccine delivery. This strategy enhances antigen presentation and elicits a strong immune response targeting tumor cells.^606^ Furthermore, nanoliposomes (mRLNPs) encoding tumor antigens and immunoadjuvants, embedded in a dynamic hyaluronic acid hydrogel, showed improved stability and efficacy at room temperature. This approach ensured sustained release and protection of mRNA, facilitating antigen presentation by dendritic cells and triggering antigen-specific CD8 T cell generation for effective tumor immunotherapy.^607^

The integration of hydrogel technologies with mRNA delivery systems marks a significant milestone in regenerative medicine and immunotherapy. This innovation introduces porous scaffolds and encapsulation techniques, enhancing treatment safety and efficacy. Such advancements facilitate the controlled release and protection of therapeutic agents, while simultaneously mimicking the cellular microenvironment to optimize therapeutic outcomes. The ongoing development of hydrogel-based delivery systems is poised to open new avenues in personalized medicine, particularly in targeted gene therapy and vaccine development. Future research aims to refine the biocompatibility, degradation rates, and payload capacity of these hydrogels, expanding their applicability across a wider spectrum of medical challenges. Moreover, the incorporation of smart hydrogels, which can respond to physiological stimuli, is expected to lead to more sophisticated, on-demand drug delivery systems.

*pDNA*: Plasmid DNA (*p*DNA), with its circular structure and autonomous replication capability, is instrumental in enhancing gene expression and facilitating cloning processes. Its precise cleavage by restriction endonucleases allows for the insertion of foreign DNA segments, making *p*DNA an essential vector in gene therapy and cloning research.^608,609^ However, the substantial molecular weight of *p*DNA poses a challenge to efficient cellular delivery due to its size and negative charge.^604^ Hydrogel technology offers a viable solution to these issues. The adjustable chemical and physical properties of hydrogels, along with their excellent biocompatibility, provide an ideal platform for *p*DNA delivery. These materials are designed to release *p*DNA in a controlled manner, preserving its stability and activity within the biological milieu. The ability of hydrogels to encapsulate large molecules, such as *p*DNA, effectively addresses the challenges of *p*DNA’s size and charge, demonstrating their superiority in gene delivery applications. Furthermore, tailoring hydrogels to specific environmental conditions enhances the efficiency, safety, and controllability of *p*DNA release.^608^ This method ensures the targeted release of therapeutic genes, retaining their bioactivity for prolonged periods and achieving delivery at precise rates and durations, highlighting the transformative potential of hydrogels in gene therapy as a powerful platform for delivering therapeutics with significant molecular weight like *p*DNA.

In inflamed wound environments, direct gene therapy faces challenges, including low transfection rates and the instability of naked DNA. To address this, a hydrogel composed of hyaluronic acid, dextran, and β-CD, loaded with plasmid DNA for vascular endothelial growth factor (*p*DNA-VEGF) and Resveratrol (Res), was developed.^610^ This innovative hydrogel, crosslinked under UV light, demonstrates enhanced mechanical strength and tailored pore properties. The β-CD’s hydrophobic center offers binding sites for Res, enhancing solubility and therapeutic efficacy. Remarkably, this hydrogel promotes rapid wound healing, reduces inflammation, and encourages vascular growth, evidenced by a marked increase in VEGF expression within 21 days (Fig. 10e). Its versatility suggests potential applications across various wound types.^610^ For transdermal *p*DNA delivery, methacrylate-based hydrogel microneedles offer a non-invasive method for tissue regeneration and cancer treatment. By bypassing the stratum corneum and evading immune detection, these microneedles enable precise *p*DNA delivery to target cells, with controlled release rates minimizing the risk of non-targeted effects.^611^ Additionally, a hybrid hydrogel scaffold combining N-carboxymethyl chitosan and sodium alginate encapsulates VEGF and TGF-β *p*DNA, utilizing arginine-modified chitosan for deep second-degree burn wound treatment. This approach leverages the hydrogel’s capacity for moisture retention and swelling to stabilize and continuously release *p*DNA, promoting wound repair through enhanced neovascularization, fibroblast proliferation, and elastin synthesis.^612^

#### Hydrogel-mediated physical intervention therapies

Physical therapies such as photothermal, photodynamic, sonodynamic, and radiation treatments have gained prominence for their therapeutic impact on refractory diseases. Central to the efficacy of these therapies are sensitizers—physically responsive agents that, upon administration, react to external stimuli to amplify the therapeutic outcomes. Nonetheless, these sensitizers also face challenges related to penetration depth, specificity, and stability, which may inadvertently lead to non-target tissue accumulation and reduced therapeutic effectiveness. Hydrogels, characterized by their biocompatibility and versatile encapsulation capabilities, offer a solution to these limitations. Well-designed hydrogels are capable of encapsulating various sensitizers, efficiently transporting them to targeted sites without compromising their functionality. Moreover, hydrogels can exploit their inherent physical and chemical properties to enhance the effectiveness of physical treatments. This synergistic integration of hydrogels with physical therapies paves the way for developing more precise and effective physiotherapeutic strategies.

##### Photothermal therapy (PTT)

PTT employs electromagnetic radiation, typically in the infrared spectrum, to generate vibrational energy (heat) for eradicating targeted cells. This therapy has emerged as a promising research area and alternative treatment for intractable tumors and wound infections caused by antibiotic-resistant bacteria. The development of PTT materials currently focuses on nanomaterials owing to their superior photothermal conversion efficiency and stability.^613,614^ However, these nanomaterials present notable drawbacks, including the potential for significant harm to healthy tissues due to their systemic distribution and the inaccuracies of laser targeting during treatment. Additionally, the accumulation of non-biodegradable nanomaterials within the body may pose long-term health risks.^615–617^

**Fig. 11:**
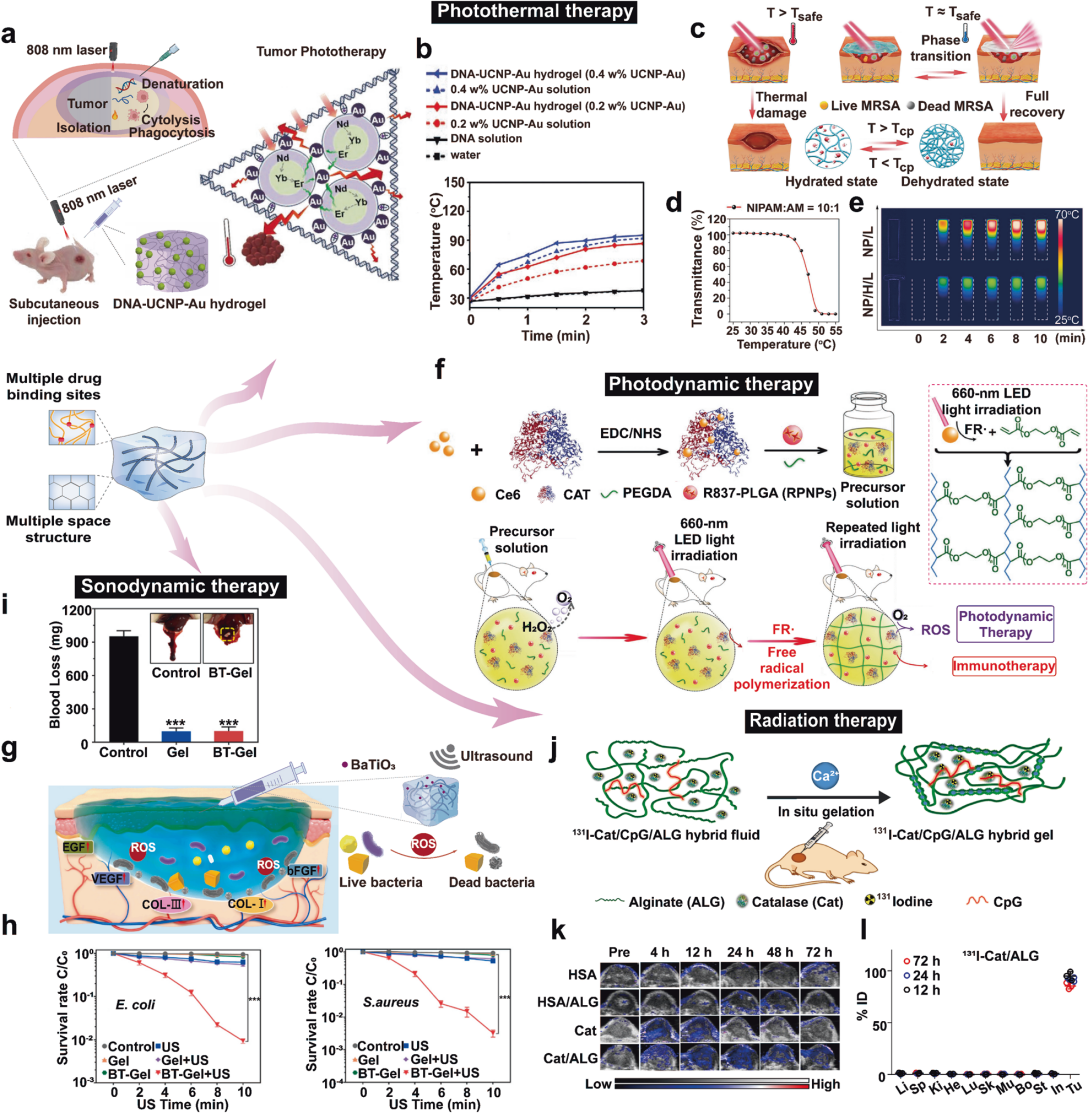
Representative examples of hydrogel-mediated physical therapies including PTT (**a**–**e**), PDT (**f**), SDT (**g**–**i**), and RT (**j**–**l)**. **a** Schematic of an DNA-inorganic hybrid hydrogel for enhanced PTT. **b** Temporal progression of the hydrogel’s temperature under NIR light irradiation. **a**, **b** reproduced with permission from ref. ^621^ Copyright 2020, John Wiley and Sons. **c** Schematic of a thermos-responsive hydrogel facilitating thermostatic PTT for improved bacteria-infected wound healing. **d** Transmittance of the gel under different temperature conditions (upon 808 nm NIR irradiation). **e** Thermal images of photothermal nanoparticles with/without the gel upon NIR irradiation. **c**–**e** reproduced from ref. ^625^ **f** Schematic of a light-triggered hydrogel incorporating Ce6-modified CAT for robust PDT. Reproduced with permission from ref. ^637^ Copyright 2019, John Wiley and Sons. **g** Schematic of an injectable self-healing hydrogel containing BaTiO_3_ for SDT aiming at improving bacterial-infected wound healing. **h** Survival rates of *E. coli* and *S. aureus* after varying durations of ultrasound treatment, demonstrating the gel’s antibacterial efficacy. **i** Assessment of blood loss in a rat liver hemorrhage model, showcasing the gel’s bioadhesive properties (Inset: representative images of a liver hemorrhage model treated with/without the bioadhesive hydrogel). **g**–**i** reproduced from ref. ^647^ **j** Schematic of a ^131^I-Cat/ALG hybrid hydrogel for localized immunostimulatory RT. **k** Photoacoustic imaging illustrating the ratio of oxygenated hemoglobin to deoxygenated hemoglobin in tumors following gel injection at various time points. **l**. Ex vivo biodistribution of ^131^I-Cat after gel injection at different time points. (Abbreviations: Li liver, Sp spleen, Ki kidney, He heart, Lu lung, Sk skin, Mu muscle, Bo bone, St stomach, In, intestine, Tu, tumor). **j**–**l** reproduced with permission from ref. ^654^ Copyright 2018, Springer Nature. Adobe Illustrator was used to generate this figure

To augment the efficacy of PTT while reducing the potential risks, investigators aim to enhance the targeting capability and localized accumulation of photothermal materials. Given this context, the injectable hydrogels can allow for precise localization of the photosensitizers at therapeutic sites. This approach not only significantly boosts photothermal conversion efficiency but also localizes PTT effectiveness, thus minimizing the systemic dispersion of photosensitizers and mitigating potential inflammation or damage to healthy tissues induced by photothermal effects.^618–620^ For instance, Liu and coworkers innovated a near-infrared (NIR)-responsive, DNA-mediated upconversion and Au nanoparticle hybrid hydrogel, enhancing photothermal efficiency up to 42.7%.^621^ The electrostatic interactions between DNA and the hybrid nanoparticles of upconversion lanthanide-Au formed a dense, stable network, shielding the nanoparticles from rapid decomposition. Upon NIR irradiation (808 nm laser), this hydrogel rapidly elevates temperatures to ~90 °C in vitro and 62.2 °C in vivo within three minutes, effectively melting cancer cell membranes and denaturing proteins, thereby inducing cell death with pronounced photothermal impact (Fig. 11a, b).^621^

Moreover, the structural network of hydrogels offers additional drug-binding sites, broadening therapeutic possibilities through the integration of PTT with other treatment modalities for enhanced therapeutic outcomes.^622^ For example, Jiang et al. developed a polyethylene glycol-based hydrogel for the co-delivery of a photosensitizer, palladium nanosheets, and the chemotherapeutic agent doxorubicin, achieving greater stability and a synergistic anticancer effect through NIR-triggered drug release.^623^ Similarly, a hydrogel combining benzylaldehyde functionalized PEG and poly(N-isopropylacrylamide) functionalized chitosan (CS-g-PNIPAAm) was engineered to co-deliver a photosensitizer (Mo154) and doxorubicin.^624^ Its dual crosslinking mechanisms involving Schiff base reactions and electrostatic interactions conferred this system self-healing property post-injection. In addition, the gel exhibited dual-responsive properties: Mo154 served as a crosslinker while also being sensitive to near-infrared radiation, enabling it to transform light into thermal energy to prompt the release of encapsulated drugs, the gel’s imine bonds also endowed its pH-responsive, allowing faster and full release of encapsulated cargos upon acidic conditions. This dual-sensitive approach facilitates the synergistic efficacy of PTT and chemotherapy, showcasing efficient tumor ablation without detectable systemic toxicity.

Another challenge of PTT is the risk of thermal damage to healthy tissues or organs from excessive energy or temperature. Hydrogel materials offer a solution by acting as carriers for photothermal agents while exploiting their intrinsic physical and chemical properties to enhance PTT efficacy. For instance, their sol-gel phase transition can be used as a thermal barrier in certain conditions to mitigate heat diffusion and safeguard normal tissues. Fu et al. have designed a thermo-responsive hydrogel to sustain a thermostatic PTT system for promoting bacterial-infected wound healing.^625^ This system harnessed NIR-induced thermal generation from nanomaterials, channeling it to the thermo-responsive hydrogel that underwent reversible phase transitions. At temperatures above its transition threshold, the hydrogel dehydrated, creating an opaque barrier (i.e., numerous light scattering centers) that limited NIR penetration. Upon cooling, it rehydrated to a transparent state, allowing for protective hydration during hydrogel removal (Fig. 11c–e).^625^ This smart hydrogel design not only eradicated bacteria and aided in wound healing but also prevented thermal damage to adjacent tissues, offering a secure and efficient method for infection management.

##### Photodynamic therapy (PDT)

PDT is another significant light-based modality, extensively explored for its efficacy especially in cancer therapy due to its distinctive attributes.^626^ PDT operates through the activation of photosensitizers localized within tumors by specific wavelengths of laser light, resulting in the generation of ROS and free radicals. These reactive agents can induce cell death through apoptosis, autophagy, and necrosis.^627,628^ The evolution of photosensitizers from the first-generation hemoporphyrin to third-generation formulations encapsulated in nanoparticle carriers marks significant advancements in increasing ROS yield, enhancing light penetration, and improving treatment efficacy while minimizing side effects.^629–633^

However, the efficacy of PDT can be greatly hampered by the oxygen-dependent nature of the treatment and the hypoxic condition prevalent in TMEs.^634^ Such conditions significantly reduce the production of effective ROS, undermining the therapeutic effectiveness of PDT.^635^ To address this, hydrogel-based delivery systems have been employed to directly transport oxygen or oxygen-generating agents, such as hemoglobin or catalytic enzymes like catalase (CAT), to the tumor site.^636^ This strategy aims to mitigate hypoxia, enhance the generation of cytotoxic ROS, and thus, augment the therapeutic efficiency of PDT. For example, Meng et al. devised a light-induced in situ gelation system, incorporating chlorin e6 (Ce6)-modified CAT.^637^ Upon administration into tumors and subsequent irradiation with red light (660 nm), this system utilized the photosensitizing properties of Ce6 to generate ROS, which triggered the polymerization of polyethylene glycol biacrylate directly within the tumor. This process effectively encapsulated CAT within the gel matrix. The immobilized CAT facilitated the conversion of the tumor’s endogenous H_2_O_2_ into O_2_, persistently alleviating hypoxia within the TMEs. This mechanism enhanced the efficacy of PDT and mitigated the immunosuppressive conditions of the TMEs, thereby promoting antitumor immune responses (Fig. 11f).^637^ In a similar vein, Zhang et al. formulated an injectable, thermosensitive hyaluronic acid hydrogel embedded with Ce6, calcium peroxide (CaO_2_), and ceria nanoparticles (CeNPs) to bolster PDT outcomes.^638^ The design of this hydrogel facilitated the permeation of water, which then reacted with CaO_2_ in the presence of CeNPs—acting as a catalyst—to continuously supply oxygen. This approach significantly mitigated hypoxia within the tumor for up to seven days, markedly improving the therapeutic effectiveness of PDT.

Admittedly, ROS produced during PDT possesses the capability to directly eliminate tumor cells. Nonetheless, an overabundance of ROS may adversely interact with adjacent agents.^639^ Especially, when ICB antibodies are jointly administered with photosensitizers, the ROS generated therein can damage the unreleased antibodies, thereby compromising the efficacy of the combined PDT and ICB therapy approach. In response to this challenge, Zhang et al. engineered a ROS-responsive hydrogel designed for the co-delivery of photosensitizers (Ce6) and ICB antibodies (anti-CD47).^4^ This hydrogel, composed of poly (deca-4,6-diynedioic acid) (PDDA), served to neutralize detrimental ROS produced during PDT, safeguarding the encapsulated antibodies. Concurrently, it initiated a ROS-responsive, gradual degradation of the hydrogel via oxidative decomposition, facilitating the controlled release of Ce6 and anti-CD47 into the TMEs. Upon exposure to light, the Ce6-produced ROS not only killed tumor cells directly but also enhanced the effectiveness of anti-CD47 therapy by sensitizing tumors with low immunogenicity. This strategic design of the hydrogel system significantly improved the combined efficacy of cancer immunotherapy and PDT, effectively preventing the recurrence and metastasis in mice bearing 4T1 tumors over the long term.

##### Sonodynamic therapy (SDT)

SDT is an emerging physical modality combining low-intensity ultrasound and sonosensitizers to operate through both thermal and mechanical effects imparted by sound waves. Thermally, sound wave propagation activates the sonosensitive agent, such as indocyanine green (ICG), facilitating the production of electrons by the sonosensitizer that reacts with oxygen to generate cytotoxic singlet oxygen species. These ROS species induce apoptosis via lipid peroxidation within the mitochondria and cell membranes. Mechanically, the penetration of sound waves generates shock waves and shear stresses, causing cellular microvibrations and pressure changes, leading to physical damage to the cells.^640^

As another novel non-invasive modality derived from PDT, SDT circumvents the limitations associated with the shallow penetration depth of photoactivation and mitigates severe skin phototoxicity associated with photosensitizers.^641^ Its enhanced soft tissue penetration capability and minimal skin phototoxicity expand the scope of SDT beyond tumor treatment, offering the potential for bactericidal applications, particularly in combination with hydrogels for the healing of bacterial-infected wounds. For one thing, the hydrogel’s inherent high-water content and three-dimensional structure offer a moist, matrix-mimicking environment as well as a physical barrier against bacterial invasion, favorable for wound regeneration.^642,643^ For another, the tunability of hydrogels’ bioadhesive properties can extend the retention of sonosensitizer at targeted wound sites, particularly crucial for wet, dynamic, and slippery tissues such as epithelia and mucosa.^644,645^ Furthermore, the introduction of reversible, dynamic bonds within the hydrogel matrix confers the material with self-healing capabilities, allowing it to resist mechanical disruption induced by ultrasound, thereby increasing the therapeutic system’s durability.^646^ When combined with SDT, these hydrogel formulations can effectively eradicate bacteria, including multidrug-resistant strains, thereby accelerating wound healing. Such combinations, whether designed as hydrogel dressings for epidermal infections or as injectable treatments for deep-seated infections (e.g., deep intramuscular abscesses, paravertebral infections, or gastrointestinal infections), are promising.

Illustratively, Liu et al. have designed an injectable self-healing hydrogel, utilizing N-[tris (hydroxymethyl) methyl] acrylamide (THM), N-(3-aminopropyl) methacrylamide hydrochloride (APMH) and oxidized hyaluronic acid (OHA), aimed at enhancing the healing process of bacteria-infected wounds.^647^ Barium titanate (BaTiO_3_, BT) nanoparticles, serving as a sonosensitizer,^648,649^ were embedded into the gel to eliminate pathogens through the accelerated generation of ROS via piezoelectric catalysis under ultrasonic stimulation. The unique tri-hydroxyl structure of THM, characterized by extensive hydrogen bonding, endowed the hydrogel with significant tissue bioadhesion capabilities, inherently restricting the BaTiO_3_ nanoparticles to the wound site. This localization confined the sonodynamic therapy (SDT)‘s ROS generation, minimizing potential damage to surrounding healthy tissues. Furthermore, introducing Schiff-base bonds between OHA and THM-APMH impart the hydrogel with swift self-healing properties, thereby enhancing the structural integrity.^650^ That prolonged the presence of the sonosensitizer within the organism and extended the duration of the SDT antimicrobial treatment. The potent efficacy of the hydrogel in bacteria clearance and wound healing, accompanied by reduced inflammation in an infected dermal defect model, underscores its pivotal contribution to the precision and effectiveness of SDT (Fig. 11g–i).^647^

##### Radiation therapy (RT)

RT, including both external radiation (comprising X-rays, γ rays, protons, or neutrons) and internal radioisotope-mediated brachytherapy, employs radiation exposure to disrupt genetic or cellular structures, thereby inducing apoptosis. This modality is widely adopted in oncological treatments.^651^ However, similar to other physical therapeutic interventions, RT also encounters the significant challenge of off-target toxicity. This is particularly evident as the dissemination of radioisotopes within non-targeted organs can lead to alterations in the DNA structure of healthy tissues, primarily due to the interactions between oxygen molecules and the free radicals generated by radiation exposure.^652^ Moreover, the effectiveness of RT is further compromised by the hypoxic characteristic of solid TMEs.^653^ Oxygen’s role is pivotal in enhancing the sensitivity of tumor cells to radiation-induced damage, as it hinders the cellular mechanisms responsible for the repair of DNA damage caused by radiation, while simultaneously facilitating an increased production of ROS. This complex interplay underscores the necessity for precise targeting, adjustment of radiation dosages, and mitigation of hypoxia in RT-mediated cancer treatments to optimize therapeutic outcomes while concurrently minimizing adverse effects.

To address these challenges, significant progress has been achieved in integrating hydrogel systems with RT. Chao and coworkers have developed a sodium alginate (ALG)-based hydrogel, embedded with immune adjuvant CpG oligonucleotide and ^131^I-labeled catalase (^131^I-CAT), to amplify RT efficacy.^654^ This enhancement is achieved by augmenting the oxygen levels within the TMEs via CAT-induced decomposition of endogenous H_2_O_2_, thereby enabling low-intensity radiation doses to exert potent antitumor effects. Upon intratumoral administration, ALG, a soluble polysaccharide, rapidly underwent cross-linking in response to the presence of multivalent cationic calcium ions within the tumor environment, thus immobilizing ^131^I-CAT within the tumor mass without systematic distribution. Furthermore, adjusting the concentration of ALG allowed for the homogeneous dispersion of ^131^I-CAT throughout the hydrogel, thereby enhancing the RT’s capability to curtail the proliferation of subcutaneous tumors and rabbit liver tumors effectively. Moreover, coupled with the blockade of immune checkpoints (anti-CTLA-4 antibodies) and the administration of immunostimulatory CpG oligonucleotide, this hydrogel-mediated localized radiotherapy not only targeted and eradicated metastatic tumors but also promoted an immune memory effect, offering a strategic avenue to prevent tumor relapse (Fig. 11j–l).^654^

Another approach is leveraging hydrogels towards combination therapy, attributed to their biocompatibility and versatility as drug carriers. This strategy is particularly noteworthy for its capacity to engender synergistic interactions among diverse therapeutic approaches, thereby enhancing the overall therapeutic beyond the limits of monotherapy. Such synergism may have the potential to reduce systemic toxicities through the strategic modulation of dosages for each therapeutic agent.^655^ The use of hydrogel systems in this context not only facilitates the co-delivery of multiple therapeutic agents but also provides varied drug-binding sites, enabling a more controlled and sustained release of drugs, thereby addressing the challenges posed by the complexity of TMEs.^656^ For instance, Mirrahimi et al. developed a thermosensitive alginate hydrogel loaded with cisplatin and AuNPs for achieving a tripartite combination of thermo-chemo-radio modality.^657^ The incorporated AuNPs acted as a potent radiosensitizer, leveraging their substantial X-ray absorption capabilities, while also functioning as a thermal source upon exposure to 532 nm laser irradiation, thereby enabling photothermal destruction of tumor cells. Subsequently, the temperature rise induced the hydrogel to degrade, thereby discharging cisplatin for a synergistic chemotherapeutic impact. This hydrogel-mediated combination led to a significantly enhanced inhibition of tumor growth compared to the monotherapy. Similarly, another injectable macroporous hydrogel has been reported as a sequential delivery platform for concurrent exploiting RT and chemotherapy for combating 4T1 breast cancer.^658^ This hydrogel was characterized by its unique two-phase release mechanism for sequential releasing radiosensitizers (bismuth nanoparticles, Bi NPs) and the chemotherapeutic agent doxorubicin. The Bi NPs were rapidly discharged within 24 h due to the hydrogel’s interconnected macroporous structure, generating hydroxyl radicals that eradicated tumor cells under X-ray irradiation. The subsequent sustained release of doxorubicin aided in preventing tumor relapse, thereby amplifying the synergistic therapeutic effect.

#### Hydrogel-mediated other non-cell therapies

Aside from their extensive applications in drug delivery and the increasingly important roles in assisting or/ and boosting the efficacy of physical therapies, hydrogels have broadened their influence into more extensive research areas within biology and medicine. The inherent and diverse characteristics of hydrogels render them indispensable in the development of bioadhesives, artificial tissues, and biosensors.^488,659–662^ Their unique and versatile properties, such as adhesiveness, hemostasis, and mechanical contraction, are critical for wound management and tissue repair.^663^ Additionally, hydrogels facilitate the creation of bioengineered organs and tissues with realistic functionalities, opening new paths in regenerative medicine.^664^ In the field of biosensors, hydrogels are driving innovations, enhancing non-invasive monitoring technologies to provide sensitive, accurate, and continuous assessments of health conditions, thereby marking a significant advancement in our capability to monitor physiological states with precision and ease.^665^ The integration of hydrogel-based technologies into the landscape of disease diagnosis and treatment offers the promise of more efficient treatment protocols, reduced recovery times, and the potential to tackle complex medical challenges that have remained elusive.

##### Bioadhesives

Inadequate management of wounds can severely inhibit the recovery of tissue or organ function, potentially leading to fatal outcomes. Suturing is the conventional technique for wound closure in clinical.^666^ Standard treatments typically involve employing sutures and staples to physically close wounds, thereby promoting healing.^667,668^ However, these conventional methods come with numerous limitations, such as damaging adjacent healthy tissue, causing tissue exudation and bleeding, and triggering inflammatory responses and scar formation.^666,669,670^ Bioadhesives, comprising substances that enable the adhesion of tissues to each other or different surfaces through biological materials, often polymerizable in situ, offer a promising alternative.^671–673^ These bioadhesives not only halt bleeding but also support the restoration of tissue structure and functionality. They are categorized into natural materials like proteins and polysaccharides, rich in functional groups (amino, carboxyl, and hydroxyl), including clinically approved fibrin glue,^674,675^ and synthetic or semisynthetic materials like cyanoacrylate, a long-standing choice for surgical adhesion.^676^ Their adhesive capabilities arise from mechanisms such as intermolecular bonding, chain entanglement, mechanical interlocking, and electrostatic interactions.^677,678^ Specifically, polysaccharides derived from natural sources possess amino groups with antibacterial properties. The formation of Schiff bases through reactions between amine and aldehyde groups, coupled with multiple cross-linking, enhances their adhesive and physical attributes.^679–681^ Hydrogels synthesized from these materials exhibit adhesive, antibacterial, self-healing properties, and notable mechanical strength, establishing them as ideal bioadhesive options.

The bioadhesive properties of hydrogels play a pivotal role in wound closure and repair. These adhesives are applied directly to the wound site to prevent infection, a risk increased by the removal of traditional dressings. For example, dressings designed for diabetic foot ulcers must possess strong adhesive qualities to accommodate movement without detaching.^682^ Beyond mere adhesion, hydrogels for extensive skin defects can leverage mechanical contraction to expedite wound healing. Sun et al. developed a hydrogel with a chitosan and EDC-NHS pretreated surface, facilitating an interpenetrating network with the skin that ensured robust integration with the tissue.^442^ Additionally, incorporating thermally responsive Nisopropylacrylamide (NIPAM) allowed the hydrogel to contract at body temperature, reducing its volume by 73.7% at 37 °C. This contraction enabled the hydrogel to tightly conform to the wound, reducing the size of the skin wound and enhancing healing. Furthermore, incorporating C_70_ fullerenes into the hydrogel mitigated cellular inflammation, inhibited cell apoptosis, and fostered fibroblast migration by modulating cellular ROS levels.

**Fig. 12:**
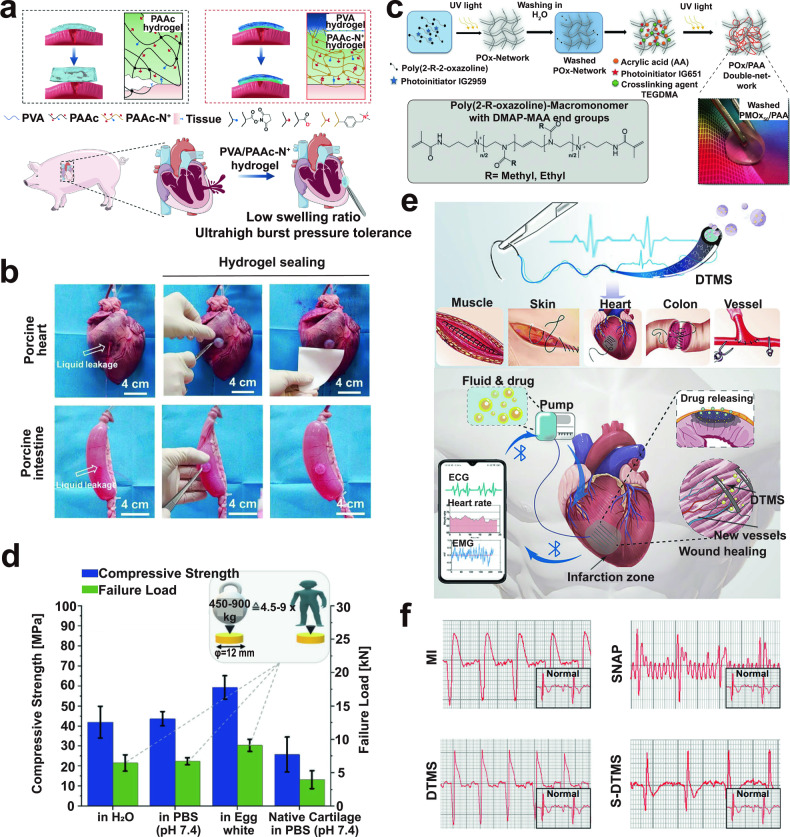
Representative example of hydrogel other non-cell therapy. Bioadhesives (**a**, **b**), artifical tissues (**c**, **d**) and biosensors (**e**, **f**). **a** Schematic representation of adhesive hydrogels. **b** Hydrogel achieve rapid and strong adhesion to ruptured heart and intestine of porcine. **a** and **b** reproduced with permission from ref. ^689^ Copyright 2023, John Wiley and Sons. **c** Steps for the synthesis of double-network hydrogels with cartilage-like mechanical properties. **d** The instantaneous recovery rate of hydrogel was higher than that of native cartilage. **c** and **d** reproduced from ref. ^704^ **e** Schematic diagram of monitoring, diagnosis, suturing, and treatment of different tissues with hydrogel sutures. **f** The effect of hydrogel sutures on monitoring abnormal electrocardiogram and treating acute myocardial infarction by preloading NO donor N-Acetyl-3-Nitroso-sulfanyl-Valine (SNAP). **e**, **f** reproduced from ref. ^727^ Adobe Illustrator was used to generate this figure

Compared to superficial skin tissue, bonding and repairing deep tissue presents more significant challenges, particularly due to the moist surface of internal wounds that demand superior adhesive strength.^683^ An effective strategy to overcome these challenges involves the development of wet adhesive hydrogels. By modifying specific groups within the hydrogel precursor polymers, these hydrogels acquire surface adhesion capabilities, addressing the unique needs of dynamic regions.^684–686^ On the basis of adhesion, the robust mechanical strength of hydrogels provides essential tension to counteract wound pressure.^687^ Phthalaldehyde (OPA) spontaneously couples with the amine group of the tissue to form a stable phthalimide bond, thus firmly bonding with the tissue.^688^ This bond’s energy dissipation under stress prevents adhesive failure, allowing for tight adhesion to various tissues in vitro, including porcine skin, liver, heart, and kidney. The development of another wet adhesive hydrogel incorporated an upper quaternary ammonium-functionalized poly(acrylic acid)-based wet-adhesive layer (PAAc-N^+^) and a ductile lower polyvinyl alcohol (PVA) layer.^689^ The PAAc-N^+^/PVA hydrogels adhered tightly to wet tissues, leveraging their rich carboxylic acid and N-hydroxysuccinimide content for mechanical interlocking capable of withstanding ultra-high burst pressures. A simple application of this hydrogel to a cardiac rupture wound for 30 s can resist high blood pressure and seal the wound effectively (Fig. 12a-b).^689^ Moreover, wet tissue adhesion hydrogels inspired by bionic mussels have been developed. Wang et al. created a biomimetic, dopamine-modified ε-polylysine-polyethylene glycol (PPD hydrogel) wound dressing, utilizing horseradish peroxidase for cross-linking.^690^ The PPD hydrogel’s excellent wet tissue adhesion properties resulted from the synergistic effect of biomimetic catechol and lysine residues. Additionally, the antibacterial nature of ε-polylysine enabled this hydrogel to serve in wound anti-infection treatments.

At the same time, internal wounds, particularly in the chest and abdomen, often result in postoperative adhesions due to contact between traumatized tissues and organs. To simultaneously repair wounds and prevent these adhesions. Wang et al. designed an asymmetric adhesive hydrogel. The hydrogel, composed of the copolymer 2-(methacryloyloxy)ethyl dimethyl-(3-sulfopropyl)ammonium hydroxide (DMAPS), facilitated electrostatic interactions within the gel network, ensuring cohesion and maintaining the gel’s internal strength.^479^ This innovative hydrogel precursor generated emulsion droplets of varying sizes based on the stirring speed during preparation, with larger droplets forming on the gel’s upper surface. This arrangement prevented the exposure of hydrophilic carboxyl groups, causing them to migrate towards the lower layer, resulting in a significant disparity in bond strength across the gel surfaces—a 20-fold difference. The bottom surface’s carboxylate groups, activated by EDC/NHS, created strong bonds with the tissue’s amino groups. Conversely, the upper surface was designed for easy detachment from tissue. This design not only ensured robust adhesion to internal wounds but also effectively mitigated adhesion on the opposite side. Additionally, the hydrogel exhibited remarkable toughness, withstanding numerous tensile and compression tests, and its pressure resistance far exceeded the maximum abdominal pressure, making it suitable for abdominal wound healing.

Beyond wound closure, hydrogels serve a crucial role in hemostasis. For example, in a case of a gelatin-polyethylene glycol (AG-PEG) hydrogel, rapid amidation of gelatin allowed the gel to quickly solidify upon administration, forming a durable barrier that halted bleeding.^691^ The positively charged AG can coagulate with negatively charged blood cells and proteins to accelerate blood coagulation. When applied to a porcine heart, the hydrogel effectively countered the heart’s dynamic changes during contraction and relaxation within 60 s, preventing cardiac perforation and extensive bleeding. Furthermore, hydrogels can function as carriers to enhance the coagulation process. Zhao et al. designed a multi-mechanism drug-loaded hemostatic hydrogel.^692^ This hydrogel utilized chitosan’s electrostatic interaction with blood cells, platelets, and plasma fibronectin to encourage aggregation, while hydrophobic segments inserted into cell membranes further assisted this process. The inclusion of a pyrogallol group increased the hydrogel’s affinity for blood cells, and gallic acid within the hydrogel speeded up the activation of blood cells and platelets. Moreover, Ca^2+^ presence facilitated the conversion of prothrombin to thrombin, quickening coagulation. Such hydrogels, characterized by their balanced swelling rate and mechanical robustness, demonstrate superior hemostatic capabilities. They encapsulate blood during the precursor expansion phase, forming a hydrogel/blood clot complex that ensures coagulation. In addition, hydrogels can be formulated as sprays for rapid, in situ gelation and hemostasis, for instance, triggered by a light-induced reaction between norbornene and four-arm PEG.^693^ The electron-rich alene group, along with a cascade amplification effect from free radicals, achieved a reaction rate surpassing traditional photopolymerization. The hydrogel’s thiol groups encouraged red blood cell aggregation by forming disulfide bonds with erythrocyte membrane sulfhydryl peptides. Moreover, the unique combination of positively charged methacrylate and borbornene groups facilitated electrostatic adsorption with negatively charged red blood cells, enhancing cell aggregation, and achieving hemostasis. Post-application, unreacted thiol, and norbornene groups can undergo further cross-linking upon light exposure, enabling the hydrogel’s self-healing properties.

##### Artificial tissues

Human tissue and organ regeneration capabilities are notably constrained, particularly following surgery, where tissue defects may result in delayed or non-healing wounds due to inadequate blood supply, as well as in the restoration and functional recovery of the central nervous system after injury.^694,695^ Consequently, developing therapies aimed at replacing or repairing these damaged tissues has emerged as a critical focus within regenerative medicine. Hydrogels, among various materials utilized in tissue engineering and regenerative medicine, have become essential for regenerating diverse tissues, including skin, bone, and nerves. This is attributed to their outstanding biocompatibility, tunable mechanical properties, and their resemblance in structure and function to natural tissues.^443,500^

The development of high-performance hydrogels that emulate human skin poses a significant challenge due to the skin’s inherent properties as a soft (Young’s modulus: 0.1–2 MPa), stretchable (140–180%), moist (cuticle moisture content of about 25%, the other parts close to 70%), and breathable biological organ.^696^ Presently, available soft materials, such as elastomers and hydrogels, only meet these complex requirements to a limited extent. Tian et al. inspired by the connective tissue structure that integrates elastic fibers with a hydrophilic matrix, introduced a straightforward and versatile method for creating elastomer-based hydrogels.^696^ This innovative approach involved using radiation to facilitate the gradual infiltration and grafting of hydrophilic monomers into an elastomer, producing a hybrid hydrogel in a singular step. The hydrogel thus obtained not only boasted high strength and excellent puncture resistance due to its crosslinked rubber network but also matched human skin in terms of friction coefficient, offering adjustable properties and ionic responsiveness. In addition, a hydrogel that structurally and functionally resembles skin (SFSH) employed double bond-decorated extracellular vesicles as crosslinkers.^697^ The incorporation of distearoylphosphatidylethanolamine-polyethylene glycol-acrylamide (DSPE-PEG-AM) into the outer membrane vesicles (OMV) derived from Nissle 1917 enhanced the hydrogel’s tensile strength, compressive strain, and toughness by increasing the vesicle’s double bond density. The swelling-induced release of bioactive substances from OMVs exhibited potent antibacterial effects and, through OMV deformation, triggered the release of endogenous antigens and immunogenic substances, activating immune responses in vivo. Beyond skin mimicry, biomimetic hydrogels can serve as artificial colon mucosa, forming a biomimetic barrier at intestinal injury sites. These hydrogels once encapsulated antibacterial agents, growth factors, and alanyl-glutamine (ALG), can offer protection against harmful bacteria, fostering intestinal epithelial regeneration, and combating inflammation in the treatment of ulcerative colitis.^698^

The dense structure, avascular nature, and the lack of self-repair capability in articular cartilage complicate the treatment of injuries through natural recovery processes. Currently, joint replacement surgery is the primary method for treating articular cartilage injuries. Yet, this approach faces challenges such as the potential failure of implant materials, necessitating frequent repairs and interventions. This not only escalates the physiological strain on patients but also imposes financial burdens.^699–701^ Against this backdrop, hydrogels emerge as promising restorative materials, offering distinct advantages. Their unique hardness and toughness effectively replicate the load-bearing function of cartilage, and their high biocompatibility ensures longevity in vivo without adverse effects.^702,703^ For instance, a novel double-network hydrogel (DNH), comprising poly(2-oxazoline)s (POx) and nonionized polyacrylic acid (PAA), exhibited mechanical properties superior to natural cartilage, making it a viable artificial cartilage alternative (Fig. 12c, d).^704^ This DNH gel’s compressive strength surpassed that of natural articular cartilage in simulated environments, and its hydrogen bonding interactions effectively dispersed mechanical stress, enhancing toughness. Furthermore, co-culturing DNH with hBMSCs demonstrated that DNH not only supported cell growth but also inhibited stem cell attachment and proliferation on its surface, marking it as an ideal cartilage-mimicking material. Similarly, a porous, double-crosslinked hydrogel made from sodium alginate (SA) and sericin (SS) displayed excellent mechanical properties and scaffold potential for chondrocyte adhesion and proliferation.^705^ By adjusting the SS content, the hydrogel’s degradation rate can be tailored to support cartilage regeneration, offering a novel approach to in situ cartilage repair.

Vascular disease significantly endangers human life and health, with conditions like coronary heart disease impeding the heart’s blood supply through coronary atherosclerosis, vascular stenosis, and occlusion. Although blood vessel transplantation offers a viable treatment, the challenges of high costs, limited availability, and potential inflammation at the harvest site limit its widespread use. These challenges have led to the exploration of synthetic materials, including biocompatible hydrogels, as alternatives for blood vessel replacement. Hydrogels, with their adjustable physicochemical properties and similarity to the extracellular matrix, stand out as excellent candidates for constructing artificial blood vessels.^706^ These hydrogels, derived from both natural and synthetic sources, facilitate the development of vascular scaffolds that encourage cell growth. For example, researchers have created bilayer hybrid scaffolds by coating decellularized vessels with a hydrogel blend of gelatin and sodium alginate, aiming to enhance mechanical stability and minimize local tissue damage while promoting cellular adhesion and proliferation within the scaffold.^707^ In addition to being used in combination with natural materials, hydrogels are also directly utilized in artificial blood vessel fabrication. Techniques like the “egg-box” model, utilizing the interaction between alginate and Ca^2+^, and electrodeposition allow for precise control over the hydrogel tube’s dimensions. Further stabilization is achieved with Ba^2+^ tertiary cross-linking, and the incorporation of type I collagen and/or silk fibroin markedly improves cellular adhesion and proliferation.^708^ By adjusting the crosslinking duration, parameters such as the artificial vessel’s diameter and wall thickness can be tailored to achieve optimal performance, demonstrating the hydrogel’s enhanced capability in supporting cell adhesion, proliferation, and survival.

##### Biosensors

Accurate and continuous measurement of key physiological parameters is crucial for health assessment, treatment planning, and postoperative monitoring. In this context, sensing technology is indispensable for real-time monitoring of physiological changes, playing a pivotal role in healthcare. Biosensors, based on their operational principles, are categorized into electrochemical, optical, and acoustic types. These sensors have broad applications across various domains, including environmental monitoring, disease diagnosis, and food safety.^709^ Despite significant advancements in biosensor technology, challenges persist in areas such as biomolecular detection speed and sensitivity, which are affected by multiple factors. The development of hydrogel sensors has emerged as a promising solution to address these limitations. Owing to their superior biocompatibility, hydrogel sensors substantially mitigate the risk of immune rejection when implanted.^710^ In particular, conductive composite hydrogels, through the addition of specific internal components or the use of unique mechanisms, exhibit enhanced sensitivity and potential for biomarker detection.^711–714^ These hydrogels can detect low concentrations of biomarkers, and visual monitoring is feasible by incorporating fluorescent substances into the hydrogel, thereby observing changes in fluorescence intensity.^715,716^ In addition, the amide groups in some hydrogels facilitate hydrogen bonding with polymers or functional groups, leading to a network structure that maintains mechanical strength under diverse conditions. This structural stability and adaptability broaden the applicability of hydrogels in biosensor technologies.^717–719^ Given their distinctive material properties and functional benefits, hydrogel sensors are poised to significantly improve biosensor performance, extend their application range, and enhance biocompatibility.

The exceptional flexibility, scalability, adhesion, and self-healing properties of hydrogels have rendered wearable hydrogel sensors highly promising for health monitoring applications.^720^ For example, a stretchable conductive hydrogel (SCH) integrated with conductive polymer nanoparticles, such as poly(3,4-ethylenedioxythiophene)-poly(styrene sulfonate) (PEDOT:PSS), have improved extensibility due to the reversible linear to curved conversion of polymer chains.^721^ This enhancement was further boosted by the inclusion of polyvinylpyrrolidone (PVP), a hydrogen bond acceptor, and polyacrylamide (PAAm), a hydrogen bond donor, increasing the hydrogel’s hydrogen bond cross-linking density. Such structural modification not only dispersed energy more efficiently but also significantly altered the hydrogel’s morphology during stretching, enhancing its extensibility. SCH exhibited remarkable durability, maintaining its original form even when stretched from 100% to 1000%, thereby offering a broad detection range that significantly broadens its monitoring capabilities and application spectrum. This feature has been applied in tracking the rehabilitation of facial paralysis by monitoring movements at the corners of the mouth to evaluate recovery, thus highlighting the practical value of hydrogel sensors in medical rehabilitation. Additionally, maskless photolithography enabled precise control over illumination localization on SCH, allowing for the integration of scalable QR codes within hydrogel strain sensors. Even when stretched, the embedded QR codes remained discernible, facilitating seamless access to stored monitoring data. This innovative blend of sensor design and information coding heralds a novel approach to data transmission and tracking in hydrogel sensor technology.

Furthermore, a polyacrylamide double-network hydrogel (AG-T-PAM DN) cross-linked with agarose and Ti_3_C_2_T_x_ nanosheets exhibited exceptional strain resistance and monitoring sensitivity due to its unique design.^722^ The robust hydrogen bonding between Ti_3_C_2_T_x_ nanosheets and the PAM chain, alongside the synergistic effects between agarose and PAM networks, resulted in an efficient energy dissipation mechanism. This enabled the hydrogel to stretch up to 4250% and withstand 1000 cycles of stretching while maintaining stable strain capacity. The sensitivity of the hydrogel increased with tensile strain, which was linked to the conductive network’s alterations, offering unparalleled sensitivity to strain. AG-T-PAM DN hydrogels’ unique properties allowed for monitoring subtle movements such as bending of fingers, wrists, neck, and facial expressions, demonstrating high sensitivity and response. Its strong adhesion enabled attachment to various material surfaces, enhancing its suitability for diverse applications in biosensing and health monitoring.

Unlike surface wearable sensors, which primarily focus on instant data acquisition, in vivo biosensors are more prone to achieve continuous monitoring of health conditions.^723^ These sensors are uniquely designed to directly transmit data to the target site, enabling the ongoing tracking of biomarker changes over time. This capability is vital for the precise evaluation of health status and the prompt commencement of necessary interventions. While traditional implantable sensors fulfill this role, they often impose challenges, including the potential need for surgical removal post-implantation. Hydrogels exhibit unique advantages through their excellent biocompatibility and adjustable biodegradability.^724,725^ By integrating specific biocompatible materials, hydrogels can minimize or prevent immune reactions, adapt seamlessly to in vivo environments, and ensure prolonged stability for in vivo usage. For instance, an innovative flexible hydrogel sensor demonstrated promising in vivo applicability by monitoring nitric oxide (NO) levels and exhibiting excellent compatibility with biological tissues, ultimately being fully bioabsorbed.^726^ This sensor comprised a copolymer of poly(L-lactic acid) and poly(trimethylene carbonate) (PLLA-PTMC), an ultrathin gold (Au) nanomembrane, and poly(eugenol). The Au nanomembrane electrode ensured consistent sensing performance, whereas poly(eugenol) degraded into non-toxic byproducts. An integrated wireless platform facilitated the continuous in vivo monitoring of NO levels, having successfully tracked NO concentrations in rabbit joint cavities over five days and achieving complete absorption after 8 weeks, eliminating the surgical removal requirement.

In addition, researchers have developed a magnetic hydrogel by embedding Neodymium-iron-boron (NdFeB) microparticles into polyvinyl alcohol (PVA).^503^ By designing the magnetic hydrogel’s dimensions, it can interact smoothly with external neodymium magnets, maintaining its position against intestinal movements or fluid flow. This magnetic hydrogel, featuring low mechanical hardness and high toughness, ensured both the structural integrity of the gel and the viability of encapsulated microorganisms, such as engineered *E. coli* Nissle 1917. This *E. coli* strain encapsulated within the hydrogel formed a bacterial biosensor that detects extracellular heme and bioluminescence, offering effective in vivo gastrointestinal bleeding detection and allowing for continuous digestive system monitoring for at least one week.

Hydrogels offer unparalleled capabilities for both immediate and continuous health monitoring and deliver targeted therapeutic interventions when necessary. A notable innovation is the diagnosis, treatment, and monitoring suture, crafted from a polyvinyl alcohol hydrogel integrated with conductive polypyrrole (Ppy), which can detect irregular electrocardiogram (ECG) signals and facilitate remote cardiac monitoring via mobile devices.^727^ This suture was designed not only to capture and wirelessly transmit motor or bioelectrical signals but also to administer drugs through its microchannel architecture. When utilized in cardiac suturing, the conductive fibers within the suture can transmit electrical signals, while an insulating outer layer can protect against electromagnetic interference from bodily tissues and external environments. Moreover, the hydrogel can be infused with the NO donor drug N-Acetyl-3-Nitroso-sulfanyl-Valine (SNAP), releasing it in response to the heart’s stretch-compression movements, more effective than simply applying SNAP (Fig. 12e, f),^727^ offering a potent treatment for myocardial infarction and significantly enhancing cardiac function. Another inventive application involves hydrogel dressings for diabetic wound care.^728^ These dressings, formed from a crosslinked hydrogel of europium- ethylenediaminetetraacetic acid (Eu-EDTA) and carboxymethyl cellulose (CMC), not only signaled the wound’s condition through fluorescent color changes but also accelerated wound healing. The mechanism involved the activation of the PI3K/Akt signaling pathway by Eu^3+^, stimulating NO production, which supported neovascularization and facilitated wound closure. These innovative applications demonstrate the great potential of hydrogels as monitoring and therapeutic tools in the medical field.

## Progress and challenges in current clinical applications of hydrogels

Despite the significant advancements in cell culture techniques, drug coupling, and scaffold design, the translation of these innovations into clinical applications faces notable challenges.^729,730^ The research progression is hindered by technical complexities, biological intricacies, and the challenges of interdisciplinary collaboration.^1,731^ Many studies on hydrogel-based cell therapies remain in the nascent stages, grappling with issues such as effective cell-scaffold integration, long-term stability, and functional maintenance. Clinical trials in tissue repair engineering and inflammation treatment are relatively rare and predominantly in preliminary phases, with a notable scarcity in tumor immunotherapy applications (Table 3). (The information presented in Tables 3 and 4 of this study was obtained from *ClinicalTrials.gov*. (https://clinicaltrials.gov/) and *ANZCTR* (https://www.anzctr.org.au/)).

**Table 3:** Representative clinical trials of cell therapy with hydrogels

Scaffold Type	Cell type	Registration number	Disease/Conditions	Phase
Fibrin hydrogel	AADSCs & MMSCs	NCT03113747	Burn wounds	II
Gelatin hydrogel	hCSCs	NCT00981006	Refractory heart failure with chronic ischemic cardiomyopathy	I
Biocomposite hydrogel	MSCs	NCT06028763	Ankle cartilage lesions	Not applicable
/	MSCs	NCT04497805	Diabetic foot ulcer	II
Collagen hydrogel	ASCs	NCT04503161	Skull base unclosure	Not applicable
Gelatin based hydrogel	SRCs	NCT02525263	Chronic kidney disease	II
/	hNTSCs & hNCs	NCT06051747	Trachea regeneration	II
Fibrin gel	hESCs	NCT02057900	Ischemic heart disease	I
Bovine collagen type I hydrogel	iPSC-CMs & stromal cells	NCT04396899	Heart failure	II
/	MSCs	NCT01879046	Osteoarthritis	Not applicable
/	MSCs	NCT02394873	Second-degree burn wounds	I
Collagen hydrogel	Chondrocytes	NCT04399239	Pinna reconstruction	II
Hyaluronic acid gel	MSCs	NCT01981330	Vocal cord scarring	I
/	BMMCs & MSCs	NCT05631444	Chronic limb-threatening ischemia	II
Collagen gel	BMMCs & MSCs	NCT02648386	Pelvic autonomic repair	II

The "/" indicates that the hydrogel type was not specified in the clinical trial

*MSCs* mesenchymal stem cells, *hCSCs* human cardiac-derived stem cells, *AADSCs* allogeneic adipose-derived stem cells, *MMSCs* multipotent mesenchymal stromal cells, *ASCs* adipose stem cells, SRCs selected renal cells, *hNTSCs* nasal cavity stem cells, *hNCs* nasal septum cartilage cells, *hESCs* human embryonic stem cells, *iPSC-CMs* induced pluripotent stem cell-derived cardiomyocytes, *BMMCs* bone marrow mononuclear cells

**Table 4:** Representative clinical trials of non-cell therapy with hydrogels

Therapy type		Scaffold	Contents	Registration number	Disease/Conditions	Phase
Drug delivery	Small molecule drugs	Pluronic ^®^ 407/188 hydrogel	Ciprofloxacin	NCT05442736	Recurrent endodontic infections	I
		Reverse thermal degradable gel	Mitomycin C	NCT01648010	Invasive carcinoma of urinary bladder	Not applicable
		NANODOX^®^ hydrogel	Doxycycline	NCT02910011	Atopic dermatitis	II
	Peptide drugs	/	RGD peptide	NCT05653245	Periodontitis	II
		/	Octreotide	NCT01295060	Acromegaly	III
	Protein drugs	/	Anti-interleukin-17A antibody	ACTRN12620000700932	Psoriatic skin	I
		Polyethylene and mineral oil gel	EPO	NCT06135259	Oral lichen planus	III
		Carbopol-based hydrogel	EPO	NCT02361931	Diabetic foot ulcer	II
		rhGM-CSF gel	rhGM-CSF	NCT01785784	Deep partial thickness burn	IV
	Gene drugs	-	-	-	-	-
Physical intervention therapy	PTT	Gelatin hydrogel	Palladium-coated gold nanorods	*	Dry eye	I
	PDT	/	Rose bengal disodium	NCT00555646	Plaque psoriasis	II
	SDT	-	-	-	-	-
	RT	hydrogel spacer	-	NCT05902390	Cervical Cancer	II
Others	Bioadhesives	10% FBR containing bioadhesive gel	FBR	NCT01192204	Squamous cell carcinoma of mouth	II
		10% lidocaine vaginal bioadhesive gel	-	NCT02465320	Acute-use anesthetic	II
		Cross-linked hyaluronan hydrogel	Cross-linked hyaluronan	NCT02166554	Postsurgical adhesions after laparoscopic gynecological surgery	II
	Artificial tissues	-	-	-	-	-
	Biosensors	-	-	-	-	-

The "/" indicates that the hydrogel type was not specified in the clinical trial. The "*" signifies the absence of a registration number but is derived from the literature.^818^

*RGD* peptide arginyl-glycyl-aspartic acid peptide, *EPO* erythropoietin, *rhGM-CSF* human granulocyte/macrophage colony-stimulating factor, *PTT* photothermal therapy, *PDT* photodynamic therapy, *SDT* sonodynamic therapy, *RT* radiation therapy, *FBR* freeze dried black raspberry

The advancement of hydrogels in drug delivery therapy is evident in the clinical trials of protein drugs, which offer higher safety and controllability, with some reaching phases III or IV. However, clinical trials involving hydrogels for gene delivery and physical intervention therapy such as SDT are rare (Table 4). Technical complexity, safety concerns, and funding limitations constrain the initiation of clinical trials for these novel technologies.^732^ Moreover, clinical translation is hampered by the complexity and safety requirements of practical applications. Before the adoption of new technologies in clinical treatment, extensive clinical trials are essential to validate their effectiveness and safety. The extrapolation of results from in vitro and animal models to humans is complicated by biological variability, necessitating a comprehensive safety assessment of hydrogels to prevent adverse effects on patients. This process demands significant time and resources to verify the feasibility and reliability of new methods for practical applications. Despite promising research progress, the journey from laboratory to clinical practice necessitates further exploration and effort. Below we outline the principal challenges encountered in the clinical translation of hydrogel-based therapies.

Injectable hydrogels, adept at encapsulating and releasing cells or drugs, and acting as effective tissue scaffolds for therapeutic responses and tissue replacement, are viewed as the most promising candidates for clinical translation. Ideally, a hydrogel’s degradation rate should match tissue regeneration rates. In drug delivery, the most effective hydrogels precisely modulate drug release rates and timing to synchronize with tissue physiological processes. However, challenges such as understanding biological degradation mechanisms, injection timing, and hydrogel repair parameters require further clarification. Additionally, controlling the degradation rate in vivo and in vitro due to various unpredictable factors remains a significant hurdle. In therapeutic contexts, ensuring targeted delivery without impacting surrounding tissues is crucial, possibly necessitating targeted strategies for enhanced treatment precision.

More critically, a significant challenge in the clinical application of hydrogel-based therapies is the absence of effective mechanisms to control or terminate the biological activity of these materials in the body, allowing for their safe deactivation or absorption when no longer needed. Furthermore, to improve delivery safety and efficacy, hydrogels are engineered with reversible cross-linking bonds or molecular recognition elements that adapt to both internal and external signals, enabling them to halt release upon certain conditions.^733^ Such innovations illustrate the potential of these delivery systems as advanced solutions for disease treatment, characterized by safe and manageable deactivation. Advancements in design are expected to enhance treatment precision and safety, potentially accelerating the clinical adoption of hydrogel delivery technologies.

Last but equally important, a thorough evaluation of the biosafety of injectable hydrogels is imperative. Questions such as the hydrogel’s ability to dissolve and be metabolized post-injection, the nature of any metabolites produced, and their potential impact on the body warrant careful investigation. Misinjections into critical areas, like major blood vessels, raising concerns for embolism, and the need for protocols to mitigate such risks, highlight the complexity of clinical applications. Additionally, the potential for hydrogels to exert undue pressure on surrounding tissues, risking injury, and the durability of their therapeutic effects over time, call for detailed studies to assess long-term outcomes. Despite advances in laboratory and animal studies, translating these findings to clinical practice is not straightforward. The human body’s intricate biological processes and interactions present a multifaceted challenge, particularly regarding hydrogels’ behavior, affected by immune responses, metabolism, and tissue compatibility. The task of mastering this complexity is daunting, emphasizing the necessity for comprehensive research to validate the effectiveness, stability, and manageability of hydrogel delivery systems in contexts mirroring clinical scenarios.

## Integration with advanced biomanufacturing technologies: frontier exploration and biomedical applications

**Fig. 13:**
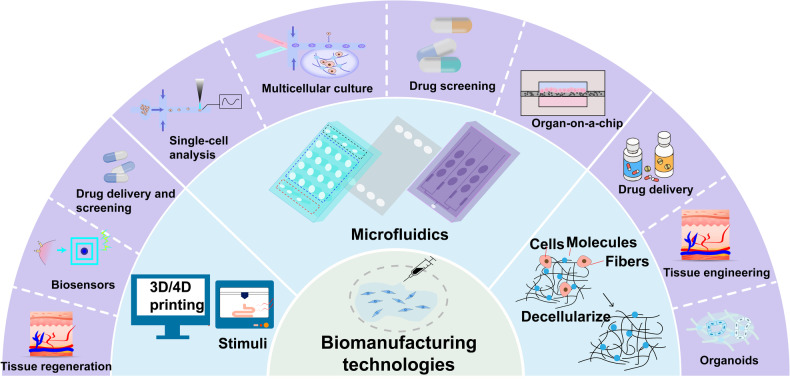
Schematic illustrates the integration of hydrogels with advanced biomanufacturing technologies, such as 3D/4D bioprinting, microfluidic technologies, and decellularization processes. It also summarizes a range of applications including biosensors, single-cell analysis, and organoids, among others. Information is collected from published work.^352,734,738,739,744,752,763,767,771^ Adobe Illustrator was used to generate this figure

With the rapid progression of biomanufacturing technologies, integrating hydrogel applications with cutting-edge techniques is essential. This integration includes the use of 3D/4D bioprinting, microfluidic chip technologies, and decellularization processes (Fig. 13). Such synergies are expected to propel the creation of more effective and precisely controllable cell therapies for a range of diseases, addressing the increasing clinical needs.

### 3D/4D bioprinting technology

3D bioprinting is a computer-assisted technology that achieves precise and controllable manipulation of biological materials through pre-designed schemes. Preprocessing and post-processing parameters must maintain the material’s biocompatibility, printing fidelity, shear dilution, mechanical strength, and other characteristics.^734^ Hydrogels emerge as an ideal material for 3D printing, offering printability, stability, and biomimicry, vital for cell, factor, and drug delivery.^735,736^

Hydrogel bioink, a key component in 3D bioprinting, is a specialized form of hydrogel that includes cells or bioactive substances. It facilitates the creation of complex biological structures, where the printability, rheological properties, swelling, surface tension, and gel dynamics of hydrogels play a pivotal role.^735,737^ Optimizing these parameters is fundamental to enhancing bioink performance. Owing to its unique properties, hydrogel bioink shows broad potential in fields like tissue engineering, drug delivery, and cell delivery, making it an ideal material for reconstructing tissue structures, achieving precise drug release, and facilitating efficient cell delivery.

In tissue engineering, hydrogels act as scaffolds to support cell growth and tissue regeneration. The integration of 3D bioprinting technology has revolutionized the fabrication of 3D functional tissues, enabling the precise placement of cells, biological materials, and growth factors within hydrogels. This process allows for layer-by-layer assembly, aiding in the development of tissues that replicate the appearance and function of natural tissues, including bone/cartilage,^738,739^ cardiovascular structure,^740,741^ skin,^742,743^ among others. Furthermore, microstructure-based biosensors, such as a biomimetic liver lobule microtissue biosensor utilizing methacrylated hyaluronic acid hydrogel, HepG2 cells, and carbon nanotubes, have shown promise in detecting substances like aflatoxin B1, exhibiting high stability and reproducibility.^744^

In the field of drug delivery, achieving precise control over the release of multiple factors within hydrogel scaffolds presents a complex challenge.^745^ The integration of drug delivery systems within 3D bioprinted scaffolds represents a promising trend.^746^ Hydrogel bioink can construct complex biological tissue structures, enabling relatively accurate drug delivery by modulating the release rate. This precision allows for the targeted release of factors at specific sites or concentrations within the 3D structure, mimicking complex cell interactions and signaling processes within the body.^746^ Additionally, the synergistic effect of combining cell therapy and drug delivery in 3D printed hydrogels has been demonstrated to promote cartilage formation in vivo.^747–749^

Moreover, the application of 3D bioprinting cell migration platforms combined with hydrogel systems spans quantitative cell and cancer biology, as well as drug screening.^750^ Developing in vitro 3D tumor models that mimic physiological cell-cell and cell-ECM interactions is crucial for high-throughput drug screening, with 3D bioprinted hydrogels playing a key role in constructing in vitro human tissue structures for potential toxicology testing and basic cell biology research.^751^

Four-dimensional (4D) bioprinting technology, building on 3D bioprinting, introduces the dimension of time, allowing printed structures to change shape, function, or performance over time in response to external stimuli such as heat, magnetism, light, humidity, and pH.^752^ The development of responsive materials, especially those responsive to temperature, is vital for 4D printing. These materials can undergo phase transitions with temperature changes, while materials responsive to magnetic fields, ROS, and pH can swell or shrink under specific conditions.^753–755^ The creation of multi-responsive hydrogels is a current research focus, with 4D printing offering more intelligent solutions that provide environmental responsiveness to artificial tissues, giving life to 3D-printed products.

### Microfluidic technology

Microfluidic technology, utilizing micrometer-scale channels and chambers, precisely manipulates small liquid volumes, excelling in fluid control, including flow, mixing, and separation of microdroplets through microchannels and microvalves.^352^ This technology contrasts with 3D bioprinting by focusing on fluid dynamics at a microscale, finding applications in cell research and drug screening. Hydrogels, expanding when hydrated, integrate seamlessly into droplet-based microfluidic systems.^756^

The microfluidic chip-driven hydrogel cell culture system can replicate the in vivo microenvironments, offering a versatile experimental platform for exploring molecular interactions at the single-cell level and the dynamics of multicellular systems.^757^ This technology allows for accurate manipulation and efficient analysis of both individuals and groups of cells, facilitating gene expression studies, protein assays, and metabolic profiling.^757^ Employing detection methods with high sensitivity and resolution enables comprehensive examinations of cell traits, enhancing our comprehension of cellular diversity, underlying disease mechanisms, and the biological underpinnings of treatment outcome.^758^ Moreover, microfluidic technology tailors microenvironmental conditions conducive to cell proliferation and differentiation,^759,760^ proving essential for cell therapy applications. For example, this approach has been reported to facilitate the precise delivery of high concentrations of ADSCs into target sites, preserving their viability and promoting muscle cell recruitment, differentiation, and growth through the sustained release of fibroblast growth factor 19.^761^ Microfluidic control over hydrogel architecture enables the creation of microfibers, and hollow or porous structures, offering consistency and efficiency in production. Notably, the porosity of hydrogel microspheres has been proven to enhance BMMSCs’ proliferation and aggregation, improving oxygen and nutrient exchange with the ECM and augmenting their paracrine functions. The uniformity and small size of these microspheres, produced via microfluidic techniques, allow for their effective dispersion in joint cavities, facilitating interaction with damaged cartilage.^762^ Additionally, leveraging microfluidic cell cultures, organ-on-a-chip (OoC) platforms that incorporate hydrogels strive to replicate human organ structures and functions. Over the last decade, the OoC field has expanded significantly, offering models that simulate virtually all human organs and physiological systems. These platforms are transforming in vitro research, with the potential to drastically minimize dependency on animal testing.^763^

Cellular response observations during drug screening improve insights into drug-induced effects and cellular variability. Microfluidic platforms, capable of conducting numerous simultaneous experiments, expedite the comprehensive screening of multiple compounds,^764^ proving instrumental in drug development stages from predictive modeling to optimization. Additionally, microfluidic technology manipulates fluid dynamics during microsphere formation by adjusting the shear force ratio between the fluid and carrier, along with the size of dimensionless droplets.^765^ This technique enables the creation of hydrogel microspheres with customizable sizes and shapes, facilitating the targeted release of medications at specific rates and times. Such precision is vital for managing chronic conditions that depend on consistent drug levels for effectiveness. For example, microfluidic methods have been used to produce uniform hydrogel microspheres measuring 265 ± 36 μm in diameter and 72 ± 5% porosity, achieving a regulated and prolonged release of Mg^2+^ over 18 days. This sustained release is crucial for the proliferation, adhesion, and mineralization of osteoblasts, which require steady concentrations of Mg^2+^.^766^ In summary, the microfluidic fabrication of hydrogel microspheres presents several benefits, including the ability to engineer complex structures, rapid production, and exact size specification, thus enhancing the versatility and efficiency of cell and drug delivery systems.^765^

### Decellularization technology

Decellularized Extracellular Matrix (dECM) technology, utilized in crafting tissue engineering scaffolds, involves removing cells from natural tissues to preserve a 3D ECM scaffold with low immunogenicity.^767^ The resulting material is then converted into hydrogels that are both mechanically tailored and chemically defined, offering improved biocompatibility and processability.^768^ These dECM hydrogels maintain the complex 3D structure and biological functions of the original tissue, making them highly compatible with tissue engineering needs for grafts. Their suitability as carriers for cell therapy and drug delivery significantly increases the success rates of cell transplants and underscores their substantial application potential. Discussions related to cell and drug delivery have been previously covered and are not repeated here.

Current research on dECM hydrogels largely centers on their use in organoid generation. Organoid culture typically involves growing precursor cells on an ECM-like substrate, creating a biomimetic microenvironment rich in natural biological, biophysical, and biomechanical signals. This setting facilitates organoid self-assembly, proliferation, differentiation, and in vitro maturation.^769^ Traditional organoid cultivation methods, however, are hampered by using biologically undefined materials and ambiguous chemical and physical properties, challenging the consistency of organoid growth conditions.^770^ dECM hydrogels introduce a novel approach to these challenges, providing clear molecular cues through their engineered mechanical and chemical properties. These cues support organoid self-assembly and maturation,^769^ positioning dECM hydrogels as an optimal biological substrate for organoid development and highlighting new avenues for research and application in organoid engineering.

Firstly, the integration of dECM technology with hydrogel’s benefits creates ECM scaffolds that maintain the complex 3D structure and biological functions post-decellularization, closely mimicking the original tissue. This generates a dynamic, biomimetic microenvironment ideal for simulating physiological tissue environments in depth. Additionally, adjusting hydrogels’ mechanical properties to align with organoid maturation kinetics and physiology improves organoid physiological simulations. Given the intricate structure of tissues and organs in vivo, tailored hydrogels are essential for designing and constructing organoids that replicate internal structures, facilitating cell self-assembly.^771^ In creating uniform organoids like pancreatic islets, dECM hydrogels’ tissue-specific signals, such as type V collagen, enhance the differentiation of iPSCs into pancreatic islet-like organoids featuring all primary endocrine cell types of the pancreas: α, β, δ, and pancreatic polypeptide cells.^772,773^ For non-uniform biomimetic structures, dECM hydrogels enable control over the organoids’ diverse structures, phenotypes, and cellular compositions, supporting the formation of specialized structures like gastrointestinal lumens and kidneys.^774–776^ This approach advances the simulation of physiological tissue environments. Therefore, the fusion of dECM technology and organoid cultivation enriches hydrogels with added functionality, incorporating biological complexity and physiological realism into the hydrogel framework. This enhancement broadens their application in tissue engineering, regenerative medicine, and drug delivery system design and research.

## Conclusion and perspective

The extensive application of hydrogels in both cell-based and non-cell-based therapies underscores their significant potential in the medical area, particularly within critical domains such as tissue engineering and cancer therapy. This review methodically examines the current design paradigms of responsive hydrogels and delves into the modulation of cell behavior by hydrogels and their utility in cell therapy. It further emphasizes the advantages and applications of hydrogels in drug delivery, as well as in various non-cell therapeutic areas, including adhesives, artificial tissues, and biosensors.

Despite notable advancements in hydrogel technology across clinical and preclinical studies, obstacles persist in their broad clinical deployment. These hurdles encompass, but are not limited to, the biocompatibility, stability, and degradation kinetics of hydrogels, which must align with tissue regeneration prerequisites.^361,777–779^ Presently, hydrogels have yet to satisfy the stringent criteria for clinical application regarding biocompatibility (e.g., biocompatibility, biodegradability) and stability (e.g., physical, chemical, and stability within biological environments).^780,781^ Such limitations impede the further evolution and application of hydrogels. Ideally, hydrogels functioning as therapeutic tissue scaffolds should demonstrate degradation rates congruent with tissue regeneration needs. However, challenges such as clarifying biodegradation mechanisms remain unaddressed. Additionally, the precise modulation of degradation rates is significantly challenged by the complexity and unpredictability of internal and external environments, further restricting their clinical utility.^781,782^ In cell therapy, current hydrogels exhibit limited efficacy in augmenting cell survival and proliferation, including immune and stem cells.^97,102,107,783,784^ Precise control over stem cell differentiation is vital for stem cell therapy, yet the regulatory capabilities of existing hydrogel systems in this regard are inadequate.^785,786^ Moreover, the accurate conveyance of therapeutic agents—including cells, small molecule drugs, and bioactive drugs—to the target site, while sparing adjacent healthy tissues, is crucial for therapeutic success.^787–789^ This necessitates the formulation of more precise and targeted therapeutic approaches to improve treatment accuracy and efficiency. In conclusion, comprehensive biocompatibility evaluation of hydrogels and precise drug delivery are imperative. In-depth research into the effects of hydrogel metabolites, long-term impacts, and controlled delivery is essential. Although in vitro and animal model studies are promising, translating these achievements to clinical practice requires navigating the complexities of human biological processes and interactions. The clinical adoption of hydrogels mandates validation in larger-scale studies to ensure efficacy, stability, and controllability.

Future research in hydrogel applications will prioritize (1) the safety and stability of hydrogel materials as central to their research and development efforts, underpinning the successful clinical translation of hydrogels as carriers of therapeutic agents. Safety is paramount to ensure hydrogels’ compatibility with the human body, mitigating immune responses or prolonged inflammation, whereas stability ensures the hydrogels’ durability and functionality within a biological environment. Furthermore, researchers should pay more attention to strategies that improve the efficacy of cell and non-cell therapy through the optimization of hydrogels’ physical characteristics, including elasticity, pore architecture, and mechanical robustness. Such optimization can mitigate the risk of adverse reactions while preserving the biocompatibility of the materials, thus providing a safer and more efficacious therapeutic approach. Precise adjustment of the hydrogels’ physical milieu not only facilitates the emulation of the natural microenvironment conducive to cell growth—enhancing cell adhesion, proliferation, and differentiation—but also allows for meticulous control of the drug release mechanism, significantly improving treatment specificity and effectiveness.

(2) The development of multifunctional and multi-responsive hydrogel platforms for cells or non-cell therapy is another critical research avenue. These platforms, which respond to various external stimuli like temperature, pH, light, and electromagnetic fields, enable more precise and targeted therapeutic agent delivery in clinical settings. Their multifunctionality, encompassing adhesion, self-healing, and antibacterial capabilities, broadens their application in adhesives, artificial tissues, and biosensors, catering to diverse medical requirements from diagnosis and therapy. The multi-responsiveness of these platforms not only facilitates specific environmental condition-dependent drug release at disease sites but also dynamically adapts their physical properties to support tissue development and maturation, offering substantial support for personalized therapies. These characteristics underscore the significant potential for clinical application of multifunctional and multi-responsive hydrogel platforms in various domains, including cancer therapy, tissue regeneration, and inflammation control.

(3) Enhancing the advantages of interdisciplinary collaboration through the integration of knowledge and technologies from materials science, cell biology, medical engineering, and clinical medicine will foster the creation of more sophisticated and effective therapeutic strategies. For example, the *application of 3D/4D printing technologies* for designing and fabricating patient-specific hydrogel scaffolds that support cell growth and tissue development. Moreover, 4D printing technology introduces the capability for printed structures to adapt to external stimuli over time, mirroring the dynamic nature of biological tissues and opening new avenues in tissue engineering and regenerative medicine. Additionally, 3D/4D printing technologies enable precise manipulation of drugs or cells within hydrogels, facilitating localized or targeted therapies to optimize therapeutic outcomes while minimizing effects on adjacent healthy tissues. *Combined with microfluidic technology*, it becomes possible to mimic the ECM at the microscale, accurately controlling drug release, cell interactions, and cellular responses to environmental changes, thus offering valuable tools for disease modeling, drug testing, and mechanistic investigations. The *incorporation of decellularized extracellular matrix technology* can produce hydrogel carriers that closely mimic natural tissue properties, creating an optimal environment for cell growth and differentiation, and showing promising potential in organoid research and applications. In conclusion, future research should continue to explore and refine safe, adaptable, and controllable therapeutic platforms based on hydrogel technology to extend their application in both cell-based and non-cell-based therapies.

## Data Availability

All data included in this study are available upon request by contact with the corresponding author.
